# Supplementary material for: Design, Synthesis, In Silico and In Vitro Studies for New Nitric Oxide-Releasing Indomethacin Derivatives with 1,3,4-Oxadiazole-2-thiol Scaffold
Source: Int J Mol Sci. 2021 Jun 30;22(13):7079. doi: 10.3390/ijms22137079 (PMC8267937; doi:10.3390/ijms22137079)
Supplement: Supplementary file 1 [file ijms-22-07079-s001.zip › ijms-1254281-supplementary.pdf]

Spectrométrie de Masse

Demande d'analyse HRMS

High-Resolution Mass Spectrometry analysis request form

Demandeur (Nom+Prénom) : Sava Alexandru<br>(*Submitter's name*) : AS	Tél :<br>(*Phone*)	Mail :<br>(*E-mail*)
Nom de l'échantillon : AS 250<br>(*Sample name*) **3a**	Confirmation :<br>☑ Structure connue<br>(Known structure)	Identification :<br>☐ Structure inconnue<br>(Unknown structure)

Formule brute : $C_9H_{11}BrO_2$<br>(*Molecular formula*)	Structure :
Masse moléculaire : 231,09<br>(*Molecular weight*)	
Solvant conseillé : CH_3OH<br>(*Recommended solvent*)	
Concentration ou masse :<br>(*Concentration or weight*)<br>1 mg / 1mL	(*Chemical structure*

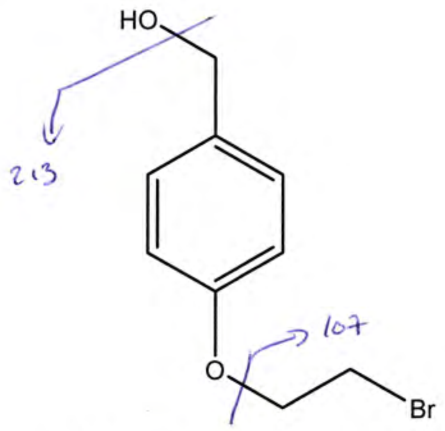

Chemical Formula: $C_9H_{11}BrO_2$
Molecular Weight: 231.09

Remarques (conservation, solvants déconseillés, toxicité…) :
(*Notes: storage, sample handling precautions, toxicity …*)

Responsable : Sylvain Routier<br>(*Supervisor*)	Date : 06.03.2018<br>(*Date*)	Bon pour accord :<br>(*Signed as agreed*)

Partie réservée au service
(*For MS facility use only*)

Position sur le rack :<br>(*Position on the rack*) GD4	Nom du fichier :<br>(*File name*) X048788CYC	Date :<br>(*Date*) 13/03/2019

Remarques :
(*Comments and suggestions*)

Cyril Colas – Plate-forme HRMS
Institut de Chimie Organique et Analytique
Université d'Orléans – UFR Sciences
Rue de Chartres – BP 6759 – F-45067 ORLEANS Cedex 2
cyril.colas@univ-orleans.fr – +33(0)2 38 49 46 61

Plate-forme de Spectrométrie de Masse Haute Résolution – Fédération de Recherche Physique et Chimie du Vivant (FR2708 : CBM/ICOA)

Analysis Info

Sample Name	**AS250**	Acquisition Date	13/03/2019 20:27:42
Analysis Name	X048788CYC_Na.d	Instrument / Ser#	maXis 255552.00086
		Method	positif-6.m

Acquisition Parameter

Source Type	ESI	Ion Polarity	Positive	Set Nebulizer	0.6 Bar
Scan Begin	50 m/z	Set Capillary	4500 V	Set Dry Heater	200 °C
Scan End	2500 m/z	Set Collision Cell RF	1800.0 Vpp	Set Dry Gas	7.0 l/min

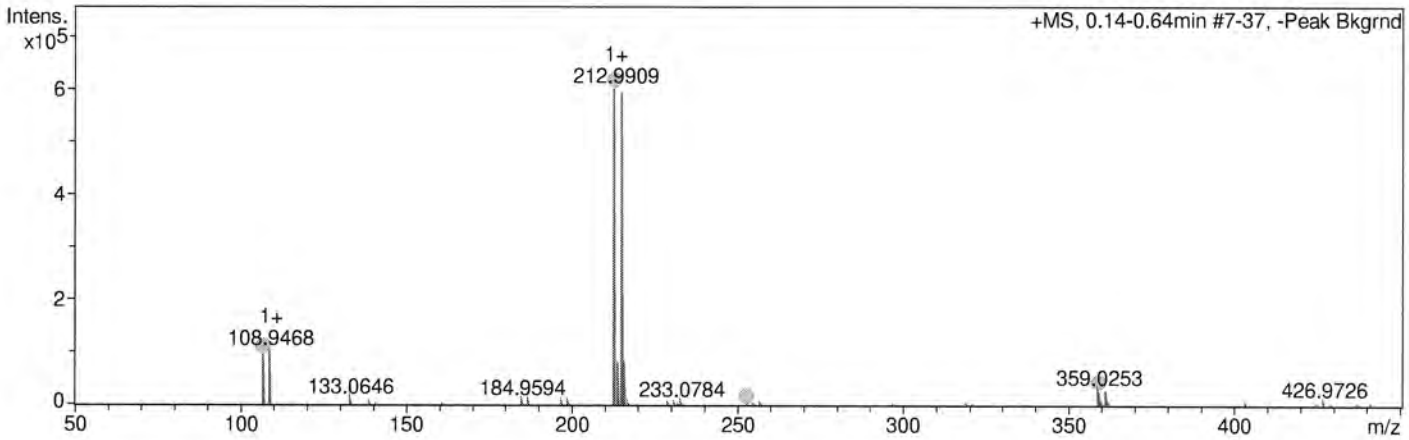

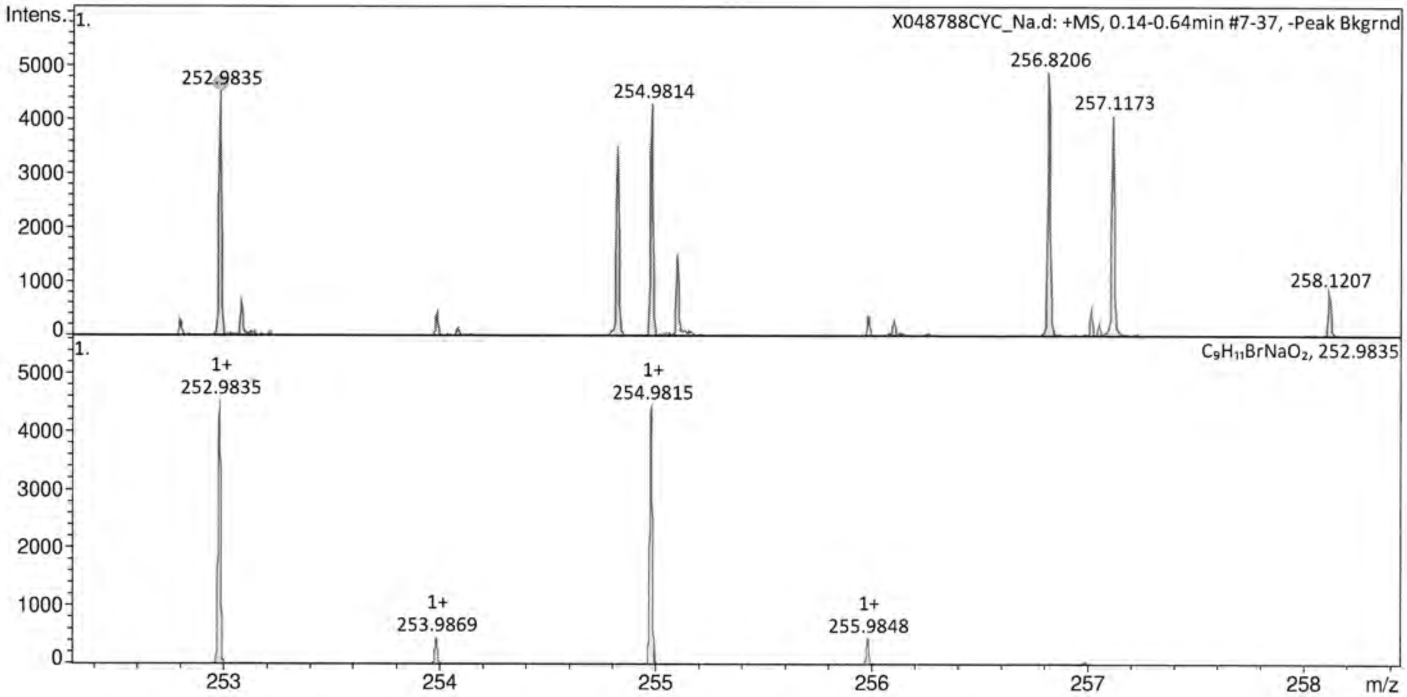

Meas. m/z	z	#	Ion Formula	m/z	err [ppm]	mSigma	rdb	e⁻ Conf
106.948832	1+	1	C2H4Br	106.949089	2.4	29.1	1.0	even
212.990920	1+	1	C9H10BrO	212.990953	0.2	30.7	5.0	even
252.983492	1+	1	C9H11BrNaO2	252.983462	-0.1	71.6	4.0	even
359.025295	1+	1	C16H17BrNaO3	359.025327	0.1	7.1	8.0	even

Spectrométrie de Masse

Demande d'analyse HRMS

High-Resolution Mass Spectrometry analysis request form

Demandeur (Nom+Prénom) : Sava Alexandru<br>(*Submitter's name*) : AS	Tél :<br>(*Phone*)	Mail :<br>(*E-mail*)

Nom de l'échantillon : AS 378<br>(*Sample name*) **3b**	Confirmation : ☑ Structure connue (Known structure)<br>Identification : ☐ Structure inconnue (Unknown structure)
Formule brute : $C_9H_{10}BrFO_2$<br>(*Molecular formula*)	Structure :(*Chemical structure*)
Masse moléculaire : 249,08<br>(*Molecular weight*)	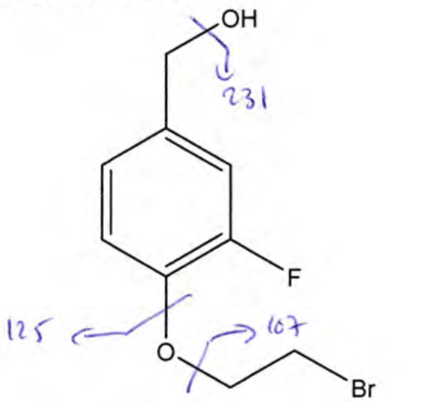
Solvant conseillé : CH_3OH<br>(*Recommended solvent*)	Chemical Formula: $C_9H_{10}BrFO_2$<br>Molecular Weight: 249.08
Concentration ou masse :<br>(*Concentration or weight*)<br>1 mg / 1mL	

Remarques (conservation, solvants déconseillés, toxicité…) :
(*Notes: storage, sample handling precautions, toxicity …*)

Responsable : Sylvain Routier<br>(*Supervisor*)	Date : 10.12.2019<br>(*Date*)	Bon pour accord :<br>(*Signed as agreed*)

Partie réservée au service
(*For MS facility use only*)

Position sur le rack :<br>(*Position on the rack*) GB11	Nom du fichier :<br>(*File name*) X053942CYC	Date :<br>(*Date*) 10/12/2019

Remarques :
(*Comments and suggestions*)

Cyril Colas – Plate-forme HRMS
Institut de Chimie Organique et Analytique
Université d'Orléans – UFR Sciences
Rue de Chartres – BP 6759 – F-45067 ORLEANS Cedex 2
cyril.colas@univ-orleans.fr – +33(0)2 38 49 46 61

Plate-forme de Spectrométrie de Masse Haute Résolution – Fédération de Recherche Physique et Chimie du Vivant (FR2708 : CBM/ICOA)

Fédération de Recherche Physique et Chimie du Vivant (FR2708 : CBM/ICOA)
Plate-forme de Spectrométrie de Masse Haute Résolution

HRAM

Analysis Info

Sample Name	**AS378**	Acquisition Date	11/12/2019 20:25:07
Analysis Name	X053942CYC_Na.d	Instrument / Ser#	maXis 255552.00086
		Method	Positif.m

Acquisition Parameter

Source Type	ESI	Ion Polarity	Positive	Set Nebulizer	0.6 Bar
Scan Begin	50 m/z	Set Capillary	4500 V	Set Dry Heater	200 °C
Scan End	2500 m/z	Set Collision Cell RF	1800.0 Vpp	Set Dry Gas	7.0 l/min

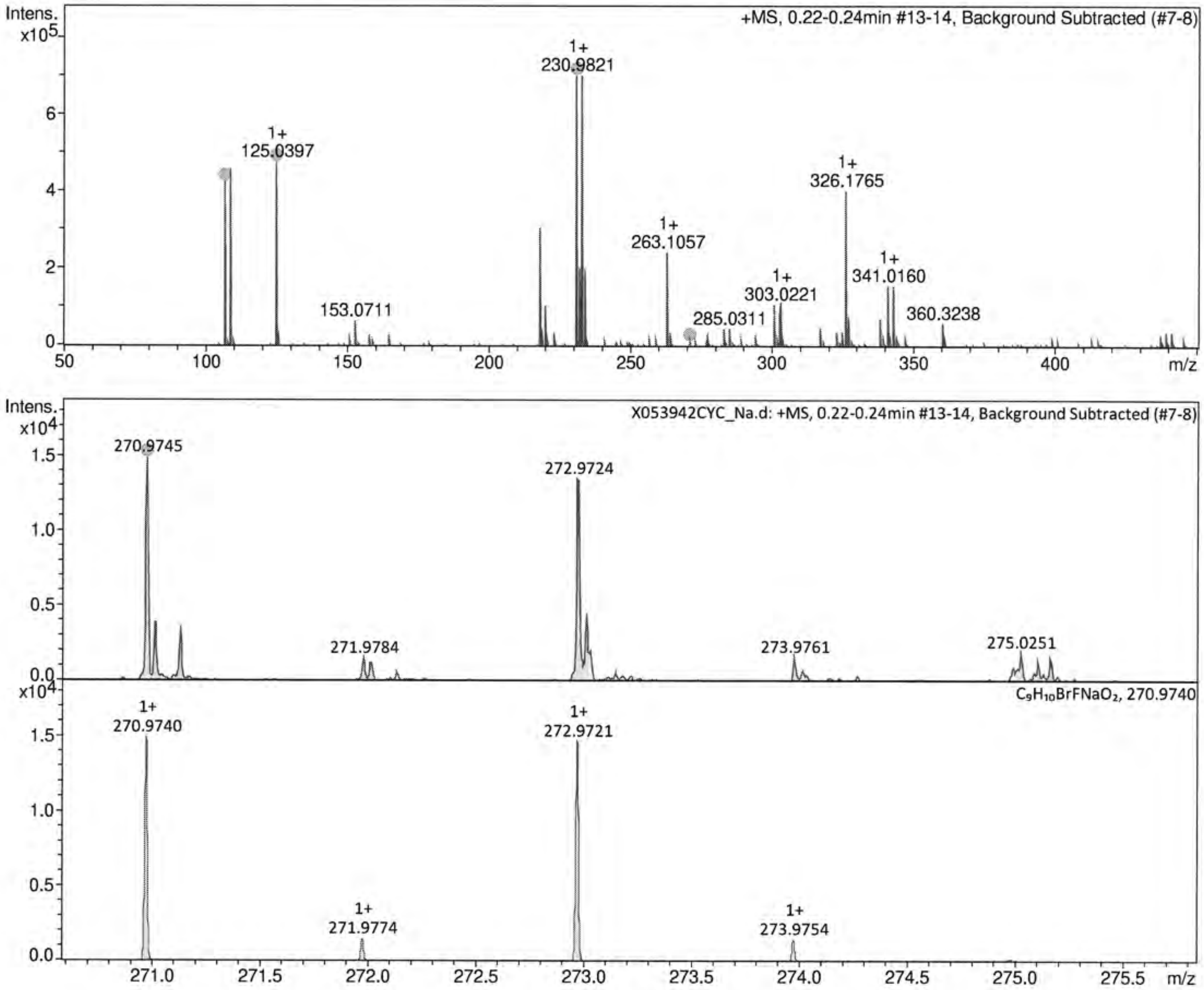

Meas. m/z	z	#	Ion Formula	m/z	err [ppm]	mSigma	rdb	e⁻ Conf
106.948928	1+	1	C2H4Br	106.949089	1.5	39.3	1.0	even
125.039736	1+	1	C7H6FO	125.039719	-0.1	2.6	5.0	even
230.982066	1+	1	C9H9BrFO	230.981532	-2.3	134.2	5.0	even
270.974533	1+	1	C9H10BrFNaO2	270.974041	-1.8	39.2	4.0	even

Institut de Chimie Organique et Analytique
UMR 7311 - Université d'Orléans - CNRS
BP 6759 - rue de Chartres - F-45067 ORLEANS Cedex 2
cyril.colas@univ-orleans.fr
+33 (0)2 38 49 46 61

Demande d'analyse HRMS

High-Resolution Mass Spectrometry analysis request form

Demandeur (Nom+Prénom) : Sava Alexandru<br>(*Submitter's name*) : AS	Tél :<br>(*Phone*)	Mail :<br>(*E-mail*)
Nom de l'échantillon : AS 363<br>(*Sample name*) **3c**	Confirmation :<br>☑ Structure connue<br>(Known structure)	Identification :<br>☐ Structure inconnue<br>(Unknown structure)

Formule brute : $C_9H_{10}BrClO_2$<br>(*Molecular formula*)	Structure :(*Chemical structure*)
Masse moléculaire : 265,53<br>(*Molecular weight*)	
Solvant conseillé : CH_3OH<br>(*Recommended solvent*)	
Concentration ou masse :<br>(*Concentration or weight*)<br>1 mg / 1mL	

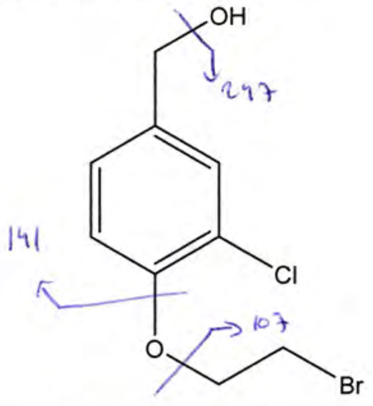

Chemical Formula: $C_9H_{10}BrClO_2$
Molecular Weight: 265.53

Remarques (conservation, solvants déconseillés, toxicité…) :
(*Notes: storage, sample handling precautions, toxicity …*)

Responsable : Sylvain Routier<br>(*Supervisor*)	Date : 10.12.2019<br>(*Date*)	Bon pour accord :<br>(*Signed as agreed*)

Partie réservée au service
(For MS facility use only)

Position sur le rack :<br>(*Position on the rack*) GA8	Nom du fichier :<br>(*File name*) X053927CYC	Date :<br>(*Date*) 10/12/2019

Remarques :
(*Comments and suggestions*)
m/z (338, 360) : contaminant
expected compound = "small" 286,9 m/z

Cyril Colas – Plate-forme HRMS
Institut de Chimie Organique et Analytique
Université d'Orléans – UFR Sciences
Rue de Chartres – BP 6759 – F-45067 ORLEANS Cedex 2
cyril.colas@univ-orleans.fr – +33(0)2 38 49 46 61

Plate-forme de Spectrométrie de Masse Haute Résolution – Fédération de Recherche Physique et Chimie du Vivant (FR2708 : CBM/ICOA)

Fédération de Recherche Physique et Chimie du Vivant (FR2708 : CBM/ICOA)
Plate-forme de Spectrométrie de Masse Haute Résolution

HRAM

Analysis Info

Sample Name	**AS363**	Acquisition Date	11/12/2019 20:20:29
Analysis Name	X053927CYC_Na.d	Instrument / Ser#	maXis 255552.00086
		Method	Positif.m

Acquisition Parameter

Source Type	ESI	Ion Polarity	Positive	Set Nebulizer	0.6 Bar
Scan Begin	50 m/z	Set Capillary	4500 V	Set Dry Heater	200 °C
Scan End	2500 m/z	Set Collision Cell RF	1800.0 Vpp	Set Dry Gas	7.0 l/min

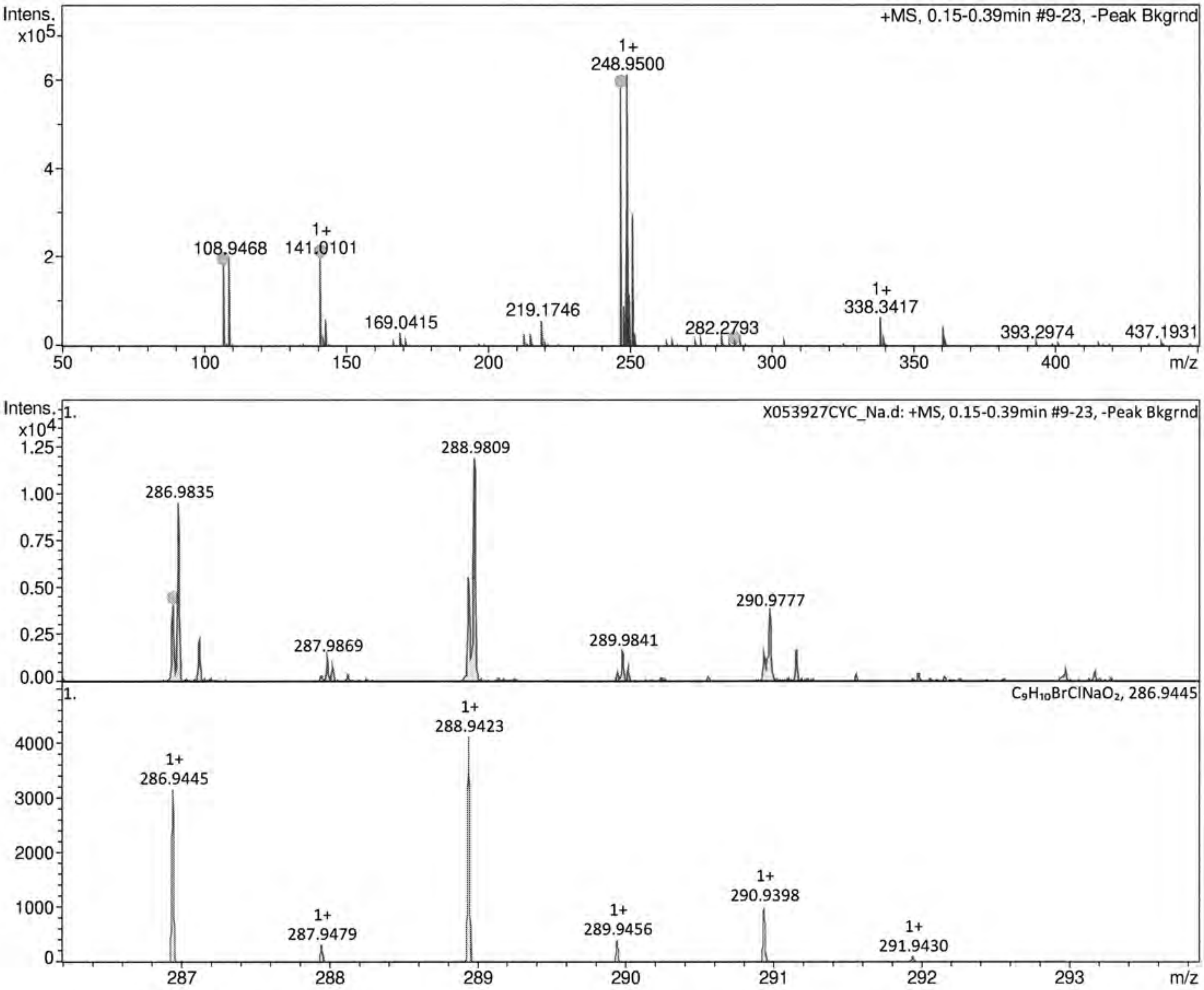

Meas. m/z	z	#	Ion Formula	m/z	err [ppm]	mSigma	rdb	e⁻ Conf
106.948856	1+	1	C2H4Br	106.949089	2.2	41.3	1.0	even
141.010140	1+	1	C7H6ClO	141.010169	0.2	9.4	5.0	even
246.952235	1+	1	C9H9BrClO	246.951981	-1.0	131.0	5.0	even
286.944829	1+	1	C9H10BrClNaO2	286.944490	-1.2	54.1	4.0	even

Institut de Chimie Organique et Analytique
UMR 7311 - Université d'Orléans - CNRS
BP 6759 - rue de Chartres - F-45067 ORLEANS Cedex 2
cyril.colas@univ-orleans.fr
+33 (0)2 38 49 46 61

printed: 12/12/2019 16:51:58

Spectrométrie de Masse

Demande d'analyse HRMS

High-Resolution Mass Spectrometry analysis request form

Demandeur (Nom+Prénom) : Sava Alexandru (*Submitter's name*) : AS	Tél : (*Phone*)	Mail : (*E-mail*)
Nom de l'échantillon : AS 301 (*Sample name*) **3d**	Confirmation : ☑ Structure connue (Known structure)	Identification : ☐ Structure inconnue (Unknown structure)

Formule brute : $C_9H_9BrCl_2O_2$ (*Molecular formula*)	Structure :
Masse moléculaire : 299,97 (*Molecular weight*)	
Solvant conseillé : CH_3OH (*Recommended solvent*)	
Concentration ou masse : (*Concentration or weight*) 1 mg / 1mL	(*Chemical structure*)

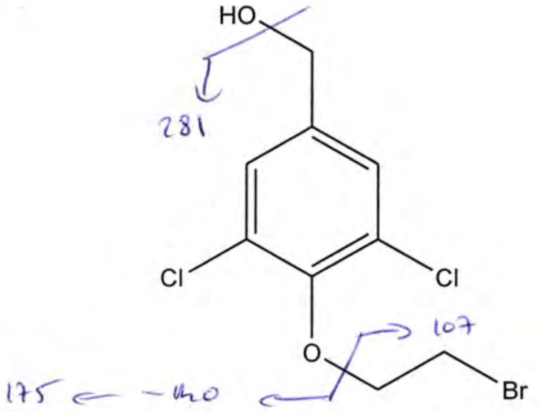

Chemical Formula: $C_9H_9BrCl_2O_2$
Molecular Weight: 299.97

Remarques (conservation, solvants déconseillés, toxicité…) :
(*Notes: storage, sample handling precautions, toxicity …*)

Responsable : Sylvain Routier (*Supervisor*)	Date : 06.03.2019 (*Date*)	Bon pour accord : (*Signed as agreed*)

Partie réservée au service
(*For MS facility use only*)

Position sur le rack : (*Position on the rack*) GE6	Nom du fichier : (*File name*) X048802CYC	Date : (*Date*) 13/03/2019

Remarques :
(*Comments and suggestions*)

Plate-forme de Spectrométrie de Masse Haute Résolution – Fédération de Recherche Physique et Chimie du Vivant (FR2708 : CBM/ICOA)

Cyril Colas – Plate-forme HRMS
Institut de Chimie Organique et Analytique
Université d'Orléans – UFR Sciences
Rue de Chartres – BP 6759 – F-45067 ORLEANS Cedex 2
cyril.colas@univ-orleans.fr – +33(0)2 38 49 46 61

Fédération de Recherche Physique et Chimie du Vivant (FR2708 : CBM/ICOA)
Plate-forme de Spectrométrie de Masse Haute Résolution

HRAM

Analysis Info

Sample Name	**AS301**	Acquisition Date	13/03/2019 21:31:39
Analysis Name	X048802CYC_Na.d	Instrument / Ser#	maXis 255552.00086
		Method	positif-6.m

Acquisition Parameter

Source Type	ESI	Ion Polarity	Positive	Set Nebulizer	0.6 Bar
Scan Begin	50 m/z	Set Capillary	4500 V	Set Dry Heater	200 °C
Scan End	2500 m/z	Set Collision Cell RF	1800.0 Vpp	Set Dry Gas	7.0 l/min

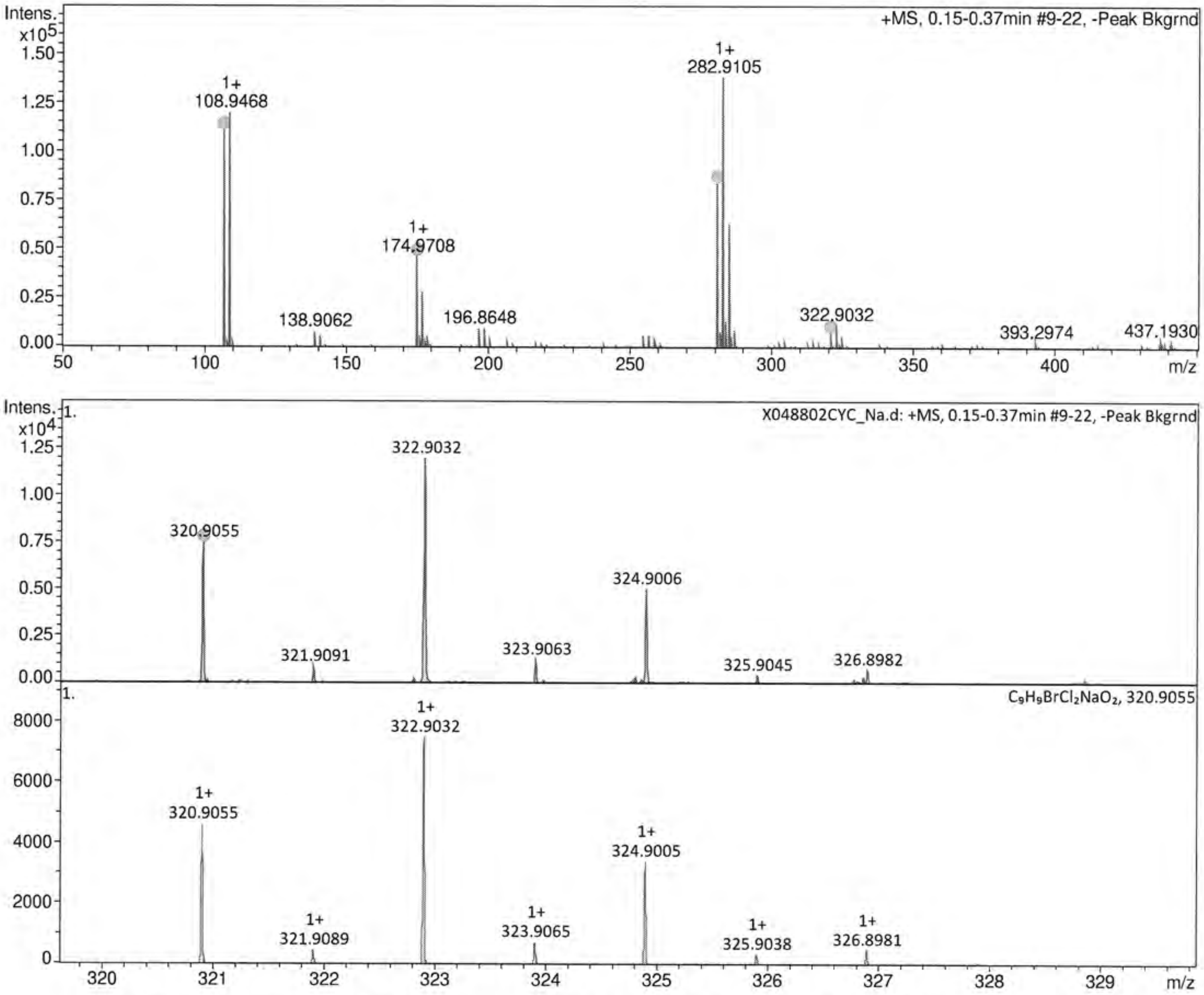

Meas. m/z	z	#	Ion Formula	m/z	err [ppm]	mSigma	rdb	e⁻ Conf
106.948765	1+	1	C2H4Br	106.949089	3.0	39.7	1.0	even
174.970843	1+	1	C7H5Cl2O	174.971197	2.0	17.4	5.0	even
280.912926	1+	1	C9H8BrCl2O	280.913009	0.3	3.8	5.0	even
320.905520	1+	1	C9H9BrCl2NaO2	320.905518	-0.0	15.2	4.0	even

Institut de Chimie Organique et Analytique
UMR 7311 - Université d'Orléans - CNRS
BP 6759 - rue de Chartres - F-45067 ORLEANS Cedex 2
cyril.colas@univ-orleans.fr
+33 (0)2 38 49 46 61

Spectrométrie de Masse

Demande d'analyse HRMS

High-Resolution Mass Spectrometry analysis request form

Demandeur (Nom+Prénom) : Sava Alexandru (*Submitter's name*) : AS	Tél : (*Phone*)	Mail : (*E-mail*)
Nom de l'échantillon : AS 362 (*Sample name*) **3e**	Confirmation : ☑ Structure connue (Known structure)	Identification : ☐ Structure inconnue (Unknown structure)

Formule brute : $C_9H_{10}BrNO_4$ (*Molecular formula*)	Structure :(*Chemical structure*)
Masse moléculaire : 276,09 (*Molecular weight*)	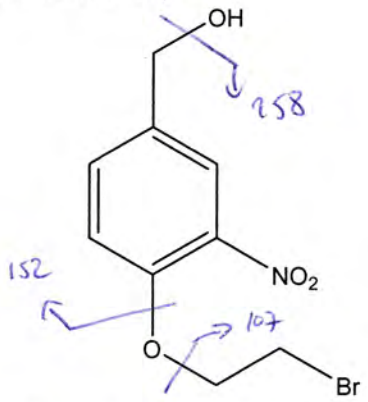
Solvant conseillé : CH_3OH (*Recommended solvent*)	Chemical Formula: $C_9H_{10}BrNO_4$
Concentration ou masse : (*Concentration or weight*) 1 mg / 1mL	Molecular Weight: 276.09

Remarques (conservation, solvants déconseillés, toxicité…) :
(*Notes: storage, sample handling precautions, toxicity …*)

Responsable : Sylvain Routier (*Supervisor*)	Date : 10.12.2019 (*Date*)	Bon pour accord : (*Signed as agreed*)

Partie réservée au service
(*For MS facility use only*)

Position sur le rack : (*Position on the rack*) GA7	Nom du fichier : (*File name*) X053926CYC	Date : (*Date*) 10/12/2019

Remarques :
(*Comments and suggestions*)

Cyril Colas – Plate-forme HRMS
Institut de Chimie Organique et Analytique
Université d'Orléans – UFR Sciences
Rue de Chartres – BP 6759 – F-45067 ORLEANS Cedex 2
cyril.colas@univ-orleans.fr – +33(0)2 38 49 46 61

Plate-forme de Spectrométrie de Masse Haute Résolution – Fédération de Recherche Physique et Chimie du Vivant (FR2708 : CBM/ICOA)

Analysis Info

Sample Name	**AS362**	Acquisition Date	11/12/2019 17:20:59
Analysis Name	X053926CYC.d	Instrument / Ser#	maXis 255552.00086
		Method	Positif.m

Acquisition Parameter

Source Type	ESI	Ion Polarity	Positive	Set Nebulizer	0.6 Bar
Scan Begin	50 m/z	Set Capillary	4500 V	Set Dry Heater	200 °C
Scan End	2500 m/z	Set Collision Cell RF	1800.0 Vpp	Set Dry Gas	7.0 l/min

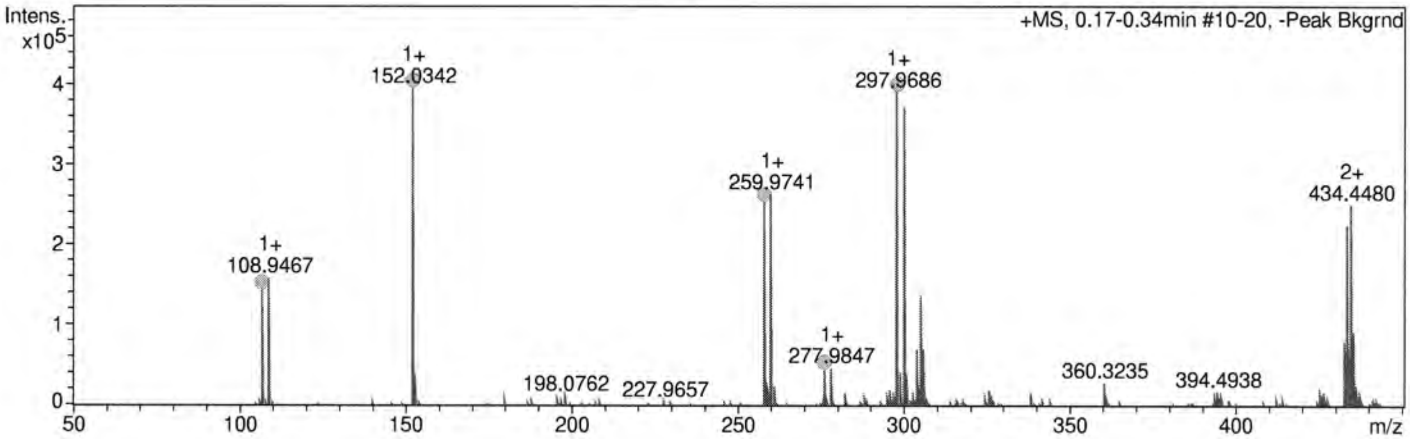

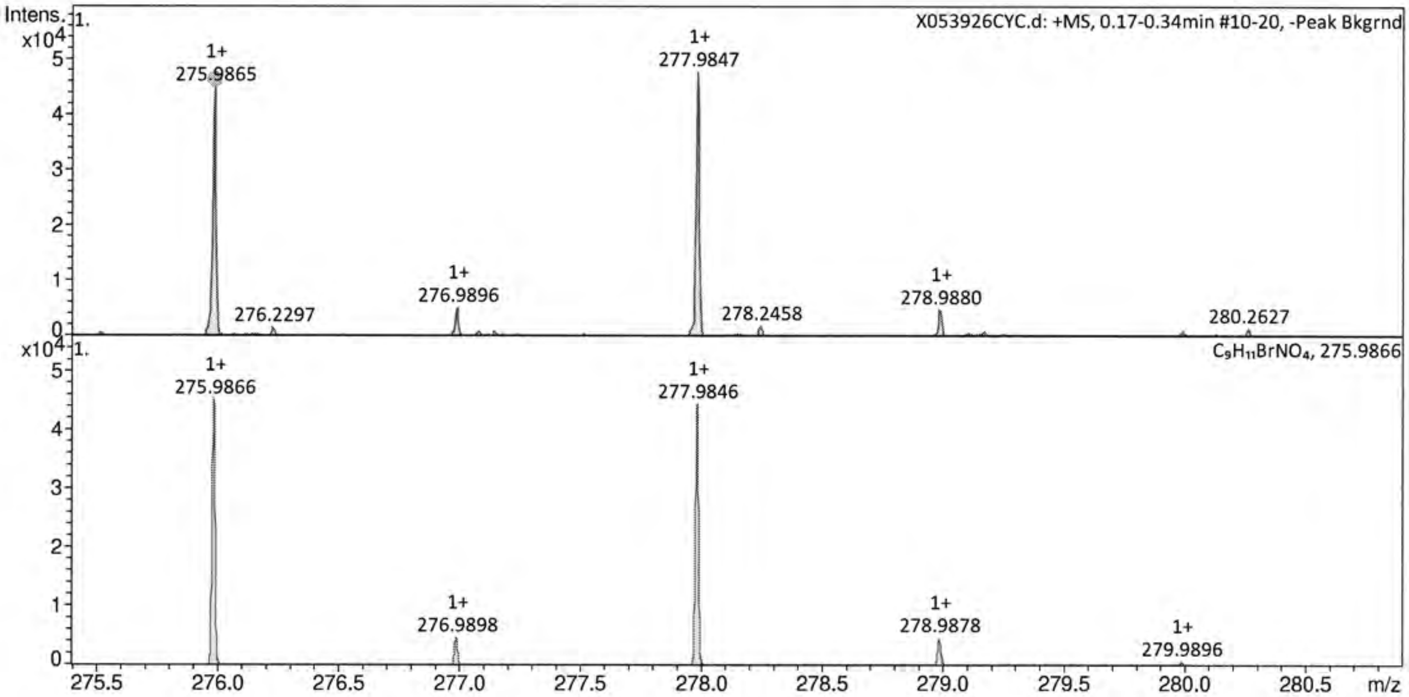

Meas. m/z	z	#	Ion Formula	m/z	err [ppm]	mSigma	rdb	e⁻ Conf
106.948745	1+	1	C2H4Br	106.949089	3.2	48.9	1.0	even
152.034223	1+	1	C7H6NO3	152.034219	-0.0	9.7	6.0	even
257.976004	1+	1	C9H9BrNO3	257.976032	0.1	19.1	6.0	even
275.986498	1+	1	C9H11BrNO4	275.986596	0.4	27.3	5.0	even
297.968602	1+	1	C9H10BrNNaO4	297.968541	-0.2	15.1	5.0	even

Spectrométrie de Masse

Demande d'analyse HRMS

High-Resolution Mass Spectrometry analysis request form

Demandeur (Nom+Prénom) : Sava Alexandru<br>(*Submitter's name*) : AS	Tél :<br>(*Phone*)	Mail :<br>(*E-mail*)
Nom de l'échantillon : AS 297<br>(*Sample name*) **3g**	Confirmation : ☑ Structure connue (Known structure)	Identification : ☐ Structure inconnue (Unknown structure)

Formule brute : $C_{11}H_{15}BrO_3$
(*Molecular formula*)

Masse moléculaire : 275,14
(*Molecular weight*)

Solvant conseillé : CH_3OH
(*Recommended solvent*)

Concentration ou masse :
(*Concentration or weight*)
1 mg / 1mL

Structure :

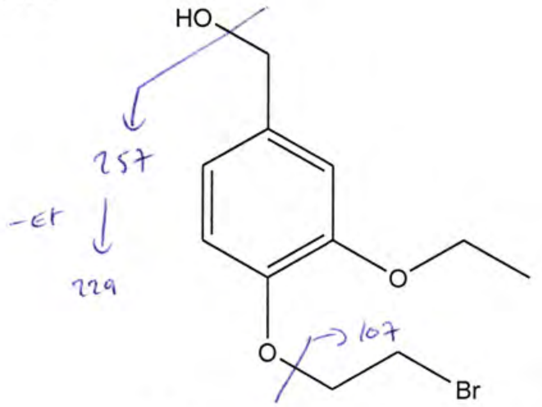

Chemical Formula: $C_{11}H_{15}BrO_3$
Molecular Weight: 275.14

(*Chemical structure*)

Remarques (conservation, solvants déconseillés, toxicité…) :
(*Notes: storage, sample handling precautions, toxicity …*)

Responsable : Sylvain Routier<br>(*Supervisor*)	Date : 06.03.2019<br>(*Date*)	Bon pour accord :<br>(*Signed as agreed*)

Partie réservée au service
(For MS facility use only)

Position sur le rack :<br>(*Position on the rack*) GE5	Nom du fichier :<br>(*File name*) X048801CYC	Date :<br>(*Date*) 13/03/2019

Remarques :
(*Comments and suggestions*)

Plate-forme de Spectrométrie de Masse Haute Résolution – Fédération de Recherche Physique et Chimie du Vivant (FR2708 : CBM/ICOA)

Cyril Colas – Plate-forme HRMS
Institut de Chimie Organique et Analytique
Université d'Orléans – UFR Sciences
Rue de Chartres – BP 6759 – F-45067 ORLEANS Cedex 2
cyril.colas@univ-orleans.fr – +33(0)2 38 49 46 61

Fédération de Recherche Physique et Chimie du Vivant (FR2708 : CBM/ICOA)
Plate-forme de Spectrométrie de Masse Haute Résolution

HRAM

Analysis Info

Sample Name	**AS297**	Acquisition Date	13/03/2019 19:38:44
Analysis Name	X048801CYC.d	Instrument / Ser#	maXis 255552.00086
		Method	positif-6.m

Acquisition Parameter

Source Type	ESI	Ion Polarity	Positive	Set Nebulizer	0.6 Bar
Scan Begin	50 m/z	Set Capillary	4500 V	Set Dry Heater	200 °C
Scan End	2500 m/z	Set Collision Cell RF	1800.0 Vpp	Set Dry Gas	7.0 l/min

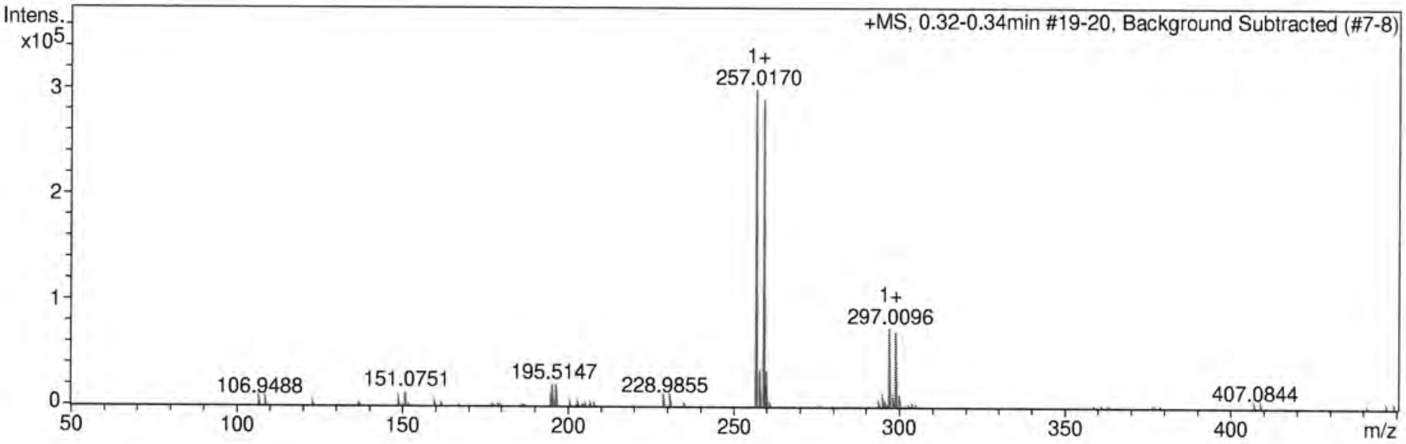

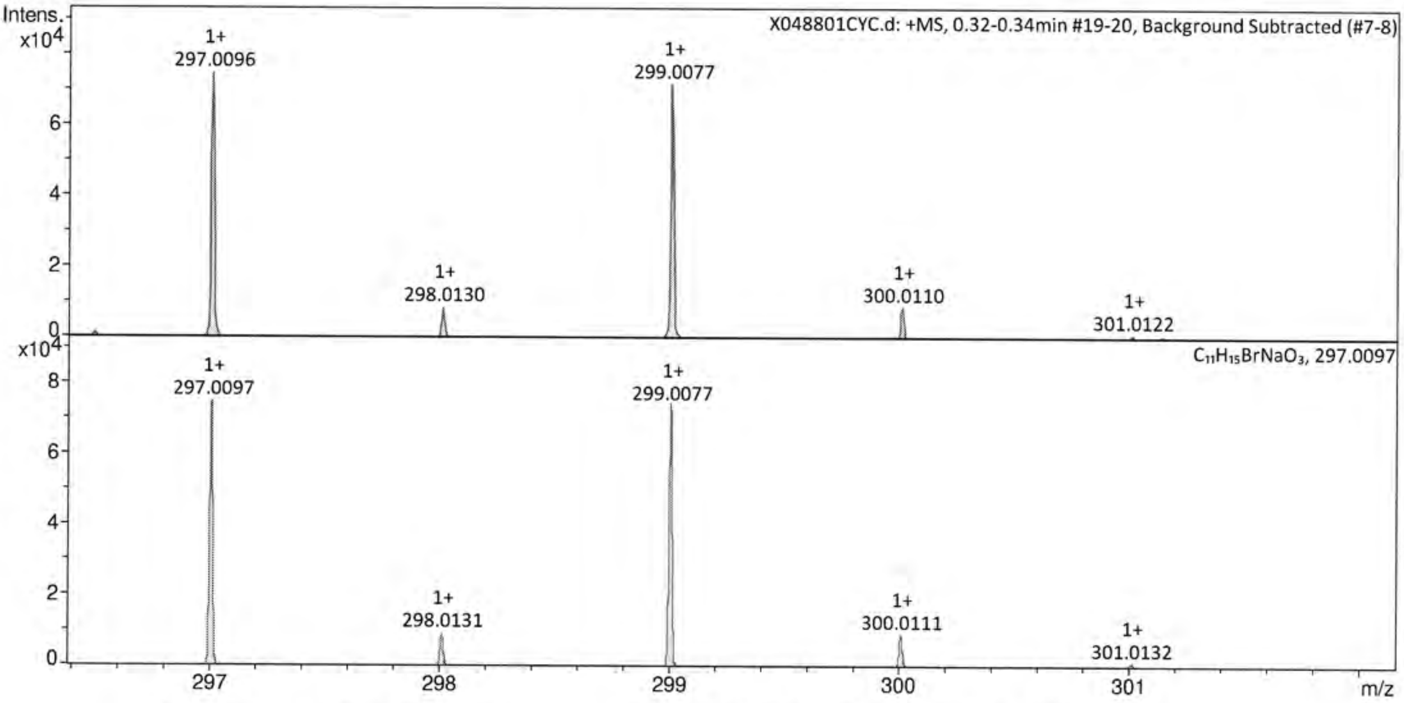

Meas. m/z	z	#	Ion Formula	m/z	err [ppm]	mSigma	rdb	e⁻ Conf
106.948763	1+	1	C2H4Br	106.949089	3.0	16.4	1.0	even
228.985531	1+	1	C9H10BrO2	228.985868	1.5	15.6	5.0	even
257.017002	1+	1	C11H14BrO2	257.017168	0.6	8.3	5.0	even
297.009633	1+	1	C11H15BrNaO3	297.009677	0.1	11.9	4.0	even

Institut de Chimie Organique et Analytique
UMR 7311 - Université d'Orléans - CNRS
BP 6759 - rue de Chartres - F-45067 ORLEANS Cedex 2
cyril.colas@univ-orleans.fr
+33 (0)2 38 49 46 61

Spectrométrie de Masse

Demande d'analyse HRMS

High-Resolution Mass Spectrometry analysis request form

Demandeur (Nom+Prénom) : Sava Alexandru<br>(*Submitter's name*) : AS	Tél :<br>(*Phone*)	Mail :<br>(*E-mail*)
Nom de l'échantillon : AS 319<br>(*Sample name*) **3h**	Confirmation :<br>☑ Structure connue<br>(Known structure)	Identification :<br>☐ Structure inconnue<br>(Unknown structure)

Formule brute : $C_{11}H_{15}BrO_4$<br>(*Molecular formula*)	Structure :
Masse moléculaire : 291,14<br>(*Molecular weight*)	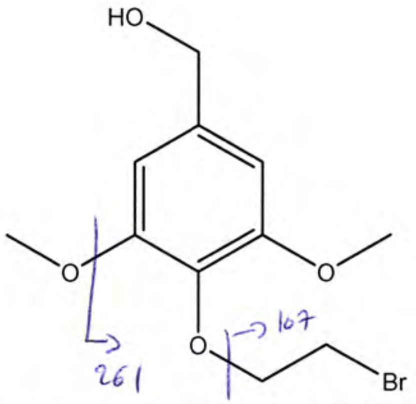
Solvant conseillé : CH_3OH<br>(*Recommended solvent*)	
Concentration ou masse :<br>(*Concentration or weight*)<br>1 mg / 1mL	(*Chemical structure*) Chemical Formula: $C_{11}H_{15}BrO_4$<br>Molecular Weight: 291.14

Remarques (conservation, solvants déconseillés, toxicité…) :
(*Notes: storage, sample handling precautions, toxicity …*)

Responsable : Sylvain Routier<br>(*Supervisor*)	Date : 12.03.2019<br>(*Date*)	Bon pour accord :<br>(*Signed as agreed*)

Partie réservée au service
(*For MS facility use only*)

Position sur le rack :<br>(*Position on the rack*) GD3	Nom du fichier :<br>(*File name*) X048787CYC	Date :<br>(*Date*) 13/03/2019

Remarques :
(*Comments and suggestions*)

Plate-forme de Spectrométrie de Masse Haute Résolution – Fédération de Recherche Physique et Chimie du Vivant (FR2708 : CBM/ICOA)

Cyril Colas – Plate-forme HRMS
Institut de Chimie Organique et Analytique
Université d'Orléans – UFR Sciences
Rue de Chartres – BP 6759 – F-45067 ORLEANS Cedex 2
cyril.colas@univ-orleans.fr – +33(0)2 38 49 46 61

Fédération de Recherche Physique et Chimie du Vivant (FR2708 : CBM/ICOA)
Plate-forme de Spectrométrie de Masse Haute Résolution

HRAM

Analysis Info

Sample Name	**AS319**	Acquisition Date	13/03/2019 17:00:52
Analysis Name	X048787CYC.d	Instrument / Ser#	maXis 255552.00086
		Method	positif-6.m

Acquisition Parameter

Source Type	ESI	Ion Polarity	Positive	Set Nebulizer	0.6 Bar
Scan Begin	50 m/z	Set Capillary	4500 V	Set Dry Heater	200 °C
Scan End	2500 m/z	Set Collision Cell RF	1800.0 Vpp	Set Dry Gas	7.0 l/min

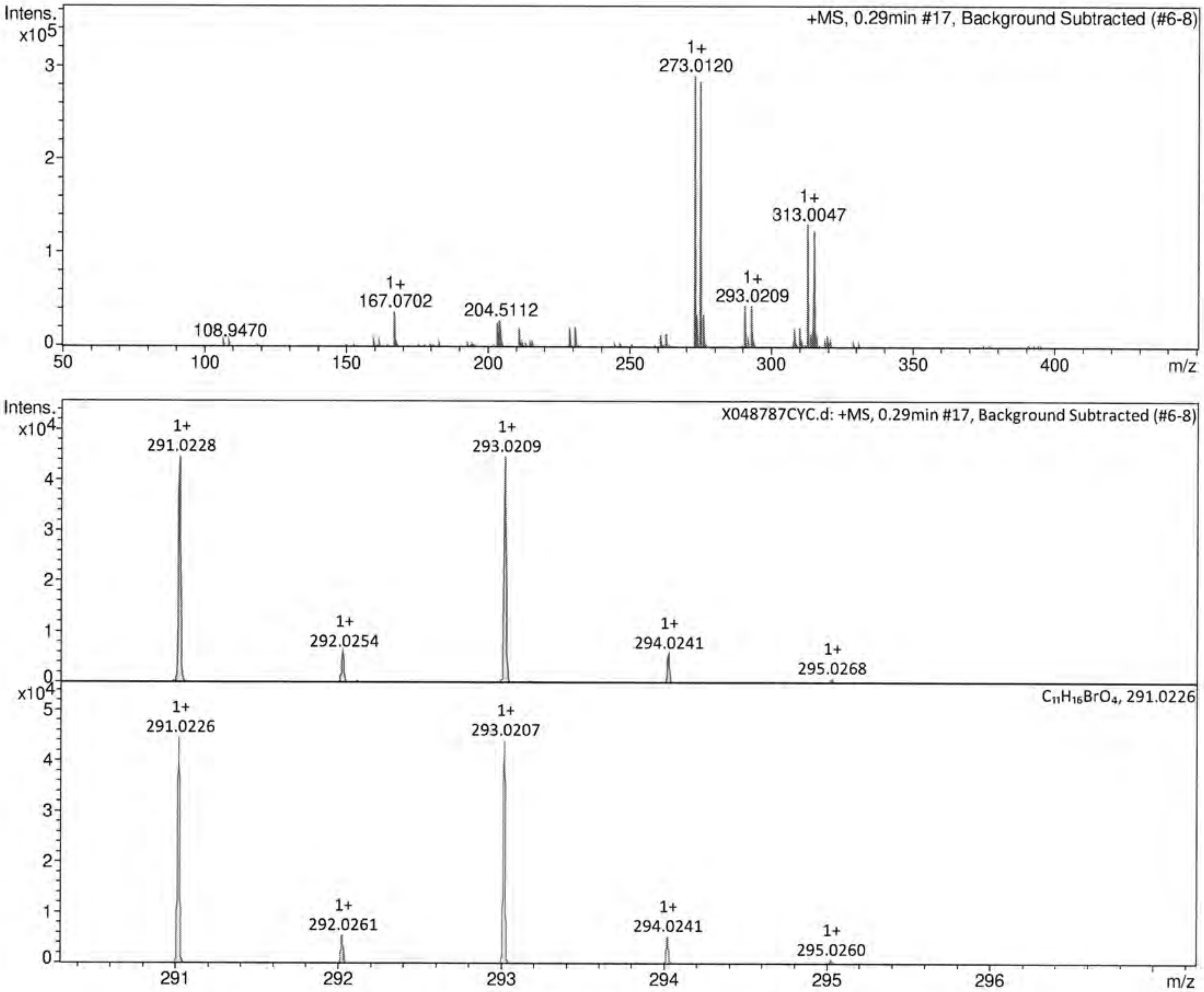

Meas. m/z	z	#	Ion Formula	m/z	err [ppm]	mSigma	rdb	e⁻ Conf
106.948896	1+	1	C2H4Br	106.949089	1.8	67.6	1.0	even
167.070172	1+	1	C9H11O3	167.070271	0.6	8.0	5.0	even
228.985811	1+	1	C9H10BrO2	228.985868	0.2	23.4	5.0	even
261.011579	1+	1	C10H14BrO3	261.012083	1.9	21.2	4.0	even
291.022764	1+	1	C11H16BrO4	291.022648	-0.4	8.9	4.0	even
308.049282	1+	1	C11H19BrNO4	308.049197	-0.3	18.4	3.0	even
313.004664	1+	1	C11H15BrNaO4	313.004592	-0.2	16.7	4.0	even

Institut de Chimie Organique et Analytique
UMR 7311 - Université d'Orléans - CNRS
BP 6759 - rue de Chartres - F-45067 ORLEANS Cedex 2
cyril.colas@univ-orleans.fr
+33 (0)2 38 49 46 61

Spectrométrie de Masse

Demande d'analyse HRMS

High-Resolution Mass Spectrometry analysis request form

Demandeur (Nom+Prénom) : Sava Alexandru<br>(*Submitter's name*) : AS	Tél :<br>(*Phone*)	Mail :<br>(*E-mail*)
Nom de l'échantillon : AS 284<br>(*Sample name*) **4a**	Confirmation :<br>☑ Structure connue<br>(Known structure)	Identification :<br>☐ Structure inconnue<br>(Unknown structure)

Formule brute : $C_9H_{11}NO_5$<br>(*Molecular formula*)	Structure :
Masse moléculaire : 213,19<br>(*Molecular weight*)	
Solvant conseillé : CH_3OH<br>(*Recommended solvent*)	
Concentration ou masse :<br>(*Concentration or weight*)<br>1 mg / 1mL	(*Chemical structure*

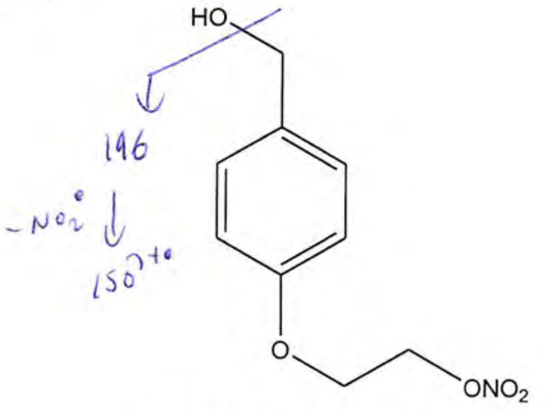

Chemical Formula: $C_9H_{11}NO_5$
Molecular Weight: 213.19

Remarques (conservation, solvants déconseillés, toxicité…) :
(*Notes: storage, sample handling precautions, toxicity …*)

Responsable : Sylvain Routier<br>(*Supervisor*)	Date : 06.03.2018<br>(*Date*)	Bon pour accord :<br>(*Signed as agreed*)

Partie réservée au service
(*For MS facility use only*)

Position sur le rack :<br>(*Position on the rack*) GH3	Nom du fichier :<br>(*File name*) X048620CYC	Date :<br>(*Date*) 06/03/2019

Remarques :
(*Comments and suggestions*)

Cyril Colas – Plate-forme HRMS
Institut de Chimie Organique et Analytique
Université d'Orléans – UFR Sciences
Rue de Chartres – BP 6759 – F-45067 ORLEANS Cedex 2
cyril.colas@univ-orleans.fr – +33(0)2 38 49 46 61

Analysis Info

Sample Name	**AS284**	Acquisition Date	06/03/2019 18:53:58
Analysis Name	X048620CYC_Na.d	Instrument / Ser#	maXis 255552.00086
		Method	Positif.m

Acquisition Parameter

Source Type	ESI	Ion Polarity	Positive	Set Nebulizer	0.6 Bar
Scan Begin	50 m/z	Set Capillary	4500 V	Set Dry Heater	200 °C
Scan End	2500 m/z	Set Collision Cell RF	1800.0 Vpp	Set Dry Gas	7.0 l/min

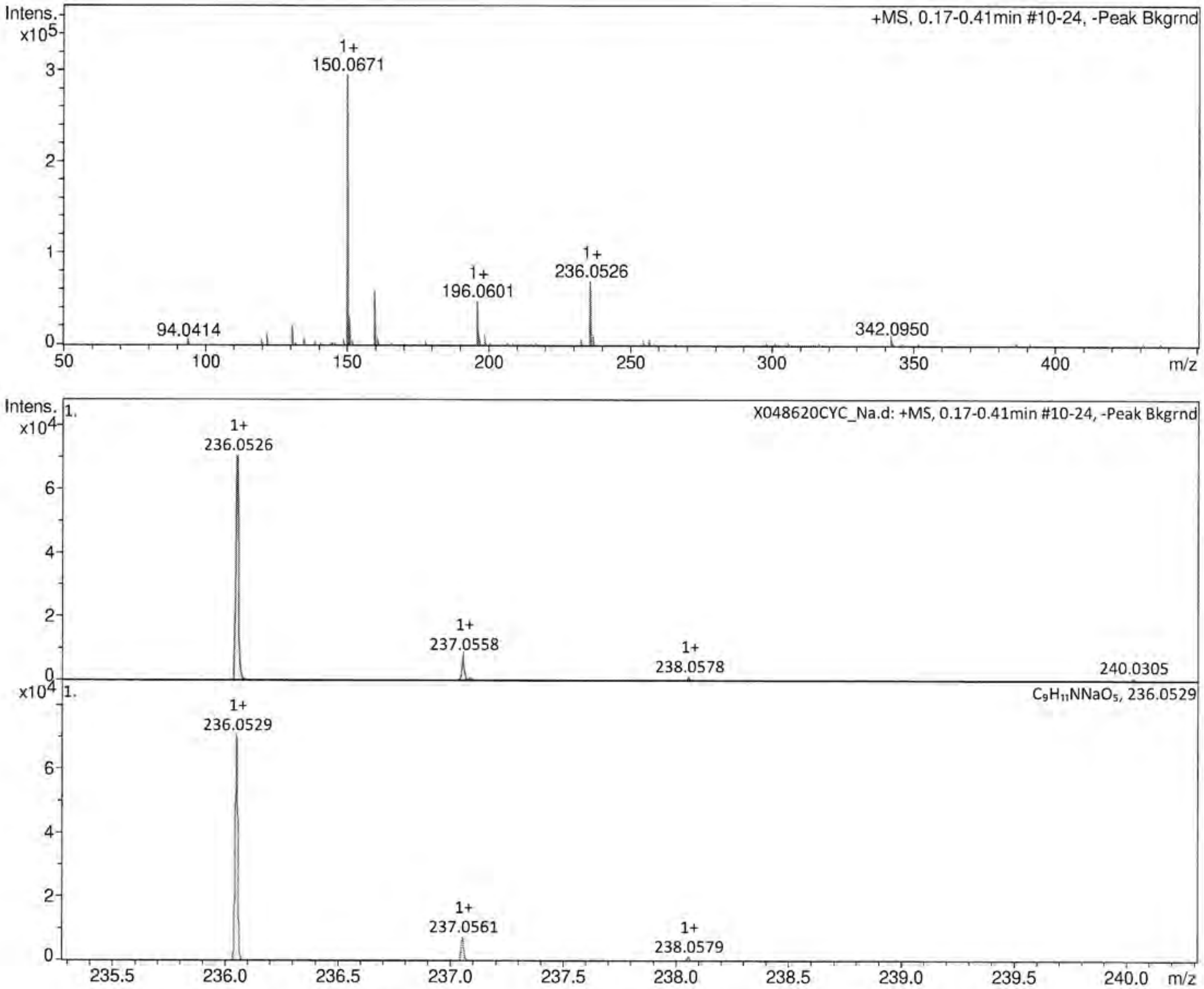

Meas. m/z	z	#	Ion Formula	m/z	err [ppm]	mSigma	rdb	e⁻ Conf
131.046529	1+	1	C7H8NaO	131.046736	1.6	2.8	4.0	even
150.067126	1+	1	C9H10O2	150.067531	2.7	6.1	5.5	odd
160.049138	1+	1	C8H9NaO2	160.049475	2.1	3.4	4.5	odd
196.060093	1+	1	C9H10NO4	196.060434	1.7	2.5	6.0	even
236.052571	1+	1	C9H11NNaO5	236.052943	1.6	5.0	5.0	even

Institut de Chimie Organique et Analytique
UMR 7311 - Université d'Orléans - CNRS
BP 6759 - rue de Chartres - F-45067 ORLEANS Cedex 2
cyril.colas@univ-orleans.fr
+33 (0)2 38 49 46 61

Spectrométrie de Masse

Demande d'analyse HRMS

High-Resolution Mass Spectrometry analysis request form

Demandeur (Nom+Prénom) : Sava Alexandru<br>(*Submitter's name*) : AS	Tél :<br>(*Phone*)	Mail :<br>(*E-mail*)

Nom de l'échantillon : AS 385
(*Sample name*)

4b

Confirmation :
☑ Structure connue
(Known structure)

Identification :
☐ Structure inconnue
(Unknown structure)

Formule brute : $C_9H_{10}FNO_5$
(*Molecular formula*)

Masse moléculaire : 231,18
(*Molecular weight*)

Solvant conseillé : CH_3OH
(*Recommended solvent*)

Concentration ou masse :
(*Concentration or weight*)

1 mg / 1mL

Structure :(*Chemical structure*)

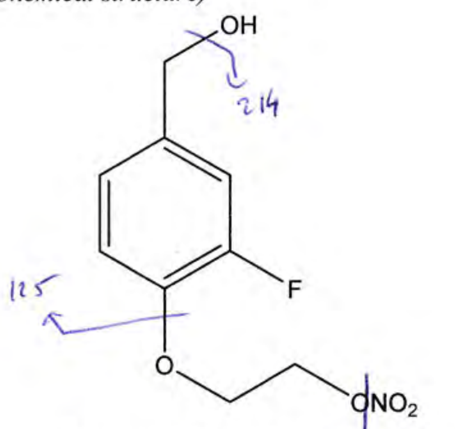

Chemical Formula: $C_9H_{10}FNO_5$
Molecular Weight: 231.18

Remarques (conservation, solvants déconseillés, toxicité…) :
(*Notes: storage, sample handling precautions, toxicity …*)

168 ← $-H_2O$ ←

Responsable : Sylvain Routier<br>(*Supervisor*)	Date : 10.12.2019<br>(*Date*)	Bon pour accord :<br>(*Signed as agreed*)

Partie réservée au service
(*For MS facility use only*)

Position sur le rack :<br>(*Position on the rack*) GC5	Nom du fichier :<br>(*File name*) X053948CYC	Date :<br>(*Date*) 10/12/2019

Remarques :
(*Comments and suggestions*)

Plate-forme de Spectrométrie de Masse Haute Résolution – Fédération de Recherche Physique et Chimie du Vivant (FR2708 : CBM/ICOA)

Cyril Colas – Plate-forme HRMS
Institut de Chimie Organique et Analytique
Université d'Orléans – UFR Sciences
Rue de Chartres – BP 6759 – F-45067 ORLEANS Cedex 2
cyril.colas@univ-orleans.fr – +33(0)2 38 49 46 61

Analysis Info

Sample Name	**AS385**	Acquisition Date	11/12/2019 20:28:06
Analysis Name	X053948CYC_Na.d	Instrument / Ser#	maXis 255552.00086
		Method	Positif.m

Acquisition Parameter

Source Type	ESI	Ion Polarity	Positive	Set Nebulizer	0.6 Bar
Scan Begin	50 m/z	Set Capillary	4500 V	Set Dry Heater	200 °C
Scan End	2500 m/z	Set Collision Cell RF	1800.0 Vpp	Set Dry Gas	7.0 l/min

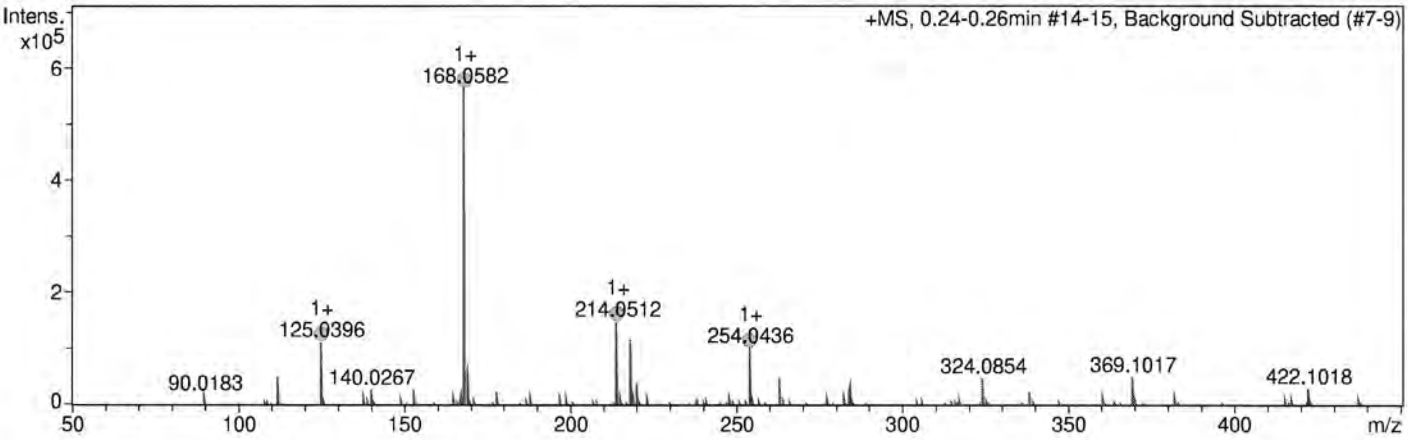

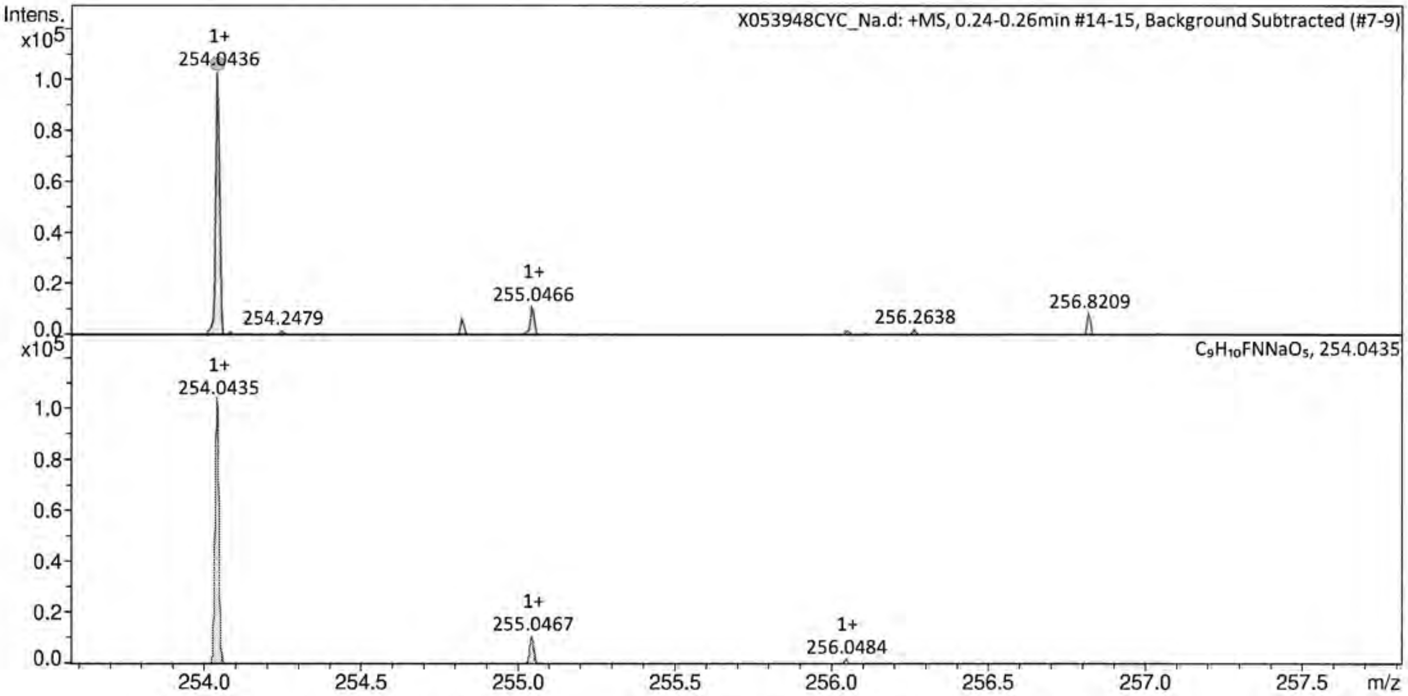

Meas. m/z	z	#	Ion Formula	m/z	err [ppm]	mSigma	rdb	e⁻ Conf
125.039593	1+	1	C7H6FO	125.039719	1.0	3.1	5.0	even
168.058180	1+	1	C9H9FO2	168.058109	-0.4	17.1	5.5	odd
214.051205	1+	1	C9H9FNO4	214.051012	-0.9	6.6	6.0	even
254.043562	1+	1	C9H10FNNaO5	254.043521	-0.2	1.4	5.0	even

Plate-forme de Spectrométrie de Masse Haute Résolution – Fédération de Recherche Physique et Chimie du Vivant (FR2708 : CBM/ICOA)

Spectrométrie de Masse

Demande d'analyse HRMS

High-Resolution Mass Spectrometry analysis request form

Demandeur (Nom+Prénom) : Sava Alexandru (*Submitter's name*) : AS	Tél : (*Phone*)	Mail : (*E-mail*)
Nom de l'échantillon : AS 375 (*Sample name*) **4c**	Confirmation : ☑ Structure connue (Known structure)	Identification : ☐ Structure inconnue (Unknown structure)

Formule brute : $C_9H_{10}ClNO_5$
(*Molecular formula*)

Masse moléculaire : 247,63
(*Molecular weight*)

Solvant conseillé : CH_3OH
(*Recommended solvent*)

Concentration ou masse :
(*Concentration or weight*)
1 mg / 1mL

Structure :(*Chemical structure*)

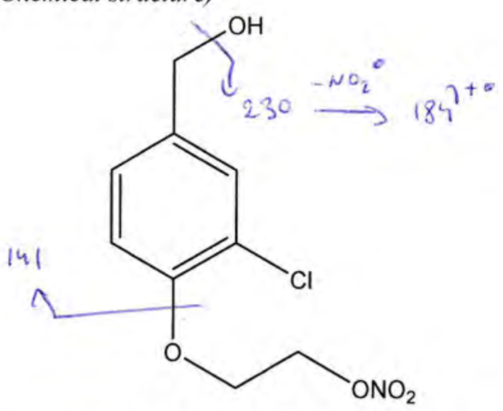

Chemical Formula: $C_9H_{10}ClNO_5$
Molecular Weight: 247.63

Remarques (conservation, solvants déconseillés, toxicité…) :
(*Notes: storage, sample handling precautions, toxicity …*)

Responsable : Sylvain Routier (*Supervisor*)	Date : 10.12.2019 (*Date*)	Bon pour accord : (*Signed as agreed*)

Partie réservée au service
(*For MS facility use only*)

Position sur le rack : (*Position on the rack*) GB8	Nom du fichier : (*File name*) X053939CYC	Date : (*Date*) 10/12/2019

Remarques :
(*Comments and suggestions*)

Cyril Colas – Plate-forme HRMS
Institut de Chimie Organique et Analytique
Université d'Orléans – UFR Sciences
Rue de Chartres – BP 6759 – F-45067 ORLEANS Cedex 2
cyril.colas@univ-orleans.fr – +33(0)2 38 49 46 61

Fédération de Recherche Physique et Chimie du Vivant (FR2708 : CBM/ICOA)
Plate-forme de Spectrométrie de Masse Haute Résolution

HRAM

Analysis Info

Sample Name	**AS375**	Acquisition Date	11/12/2019 20:22:09
Analysis Name	X053939CYC_Na.d	Instrument / Ser#	maXis 255552.00086
		Method	Positif.m

Acquisition Parameter

Source Type	ESI	Ion Polarity	Positive	Set Nebulizer	0.6 Bar
Scan Begin	50 m/z	Set Capillary	4500 V	Set Dry Heater	200 °C
Scan End	2500 m/z	Set Collision Cell RF	1800.0 Vpp	Set Dry Gas	7.0 l/min

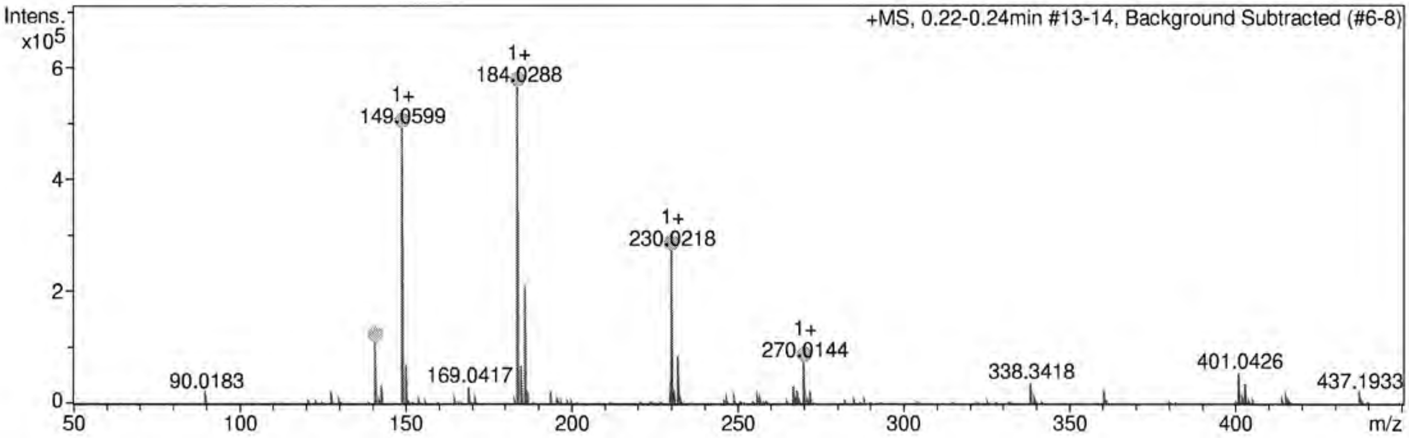

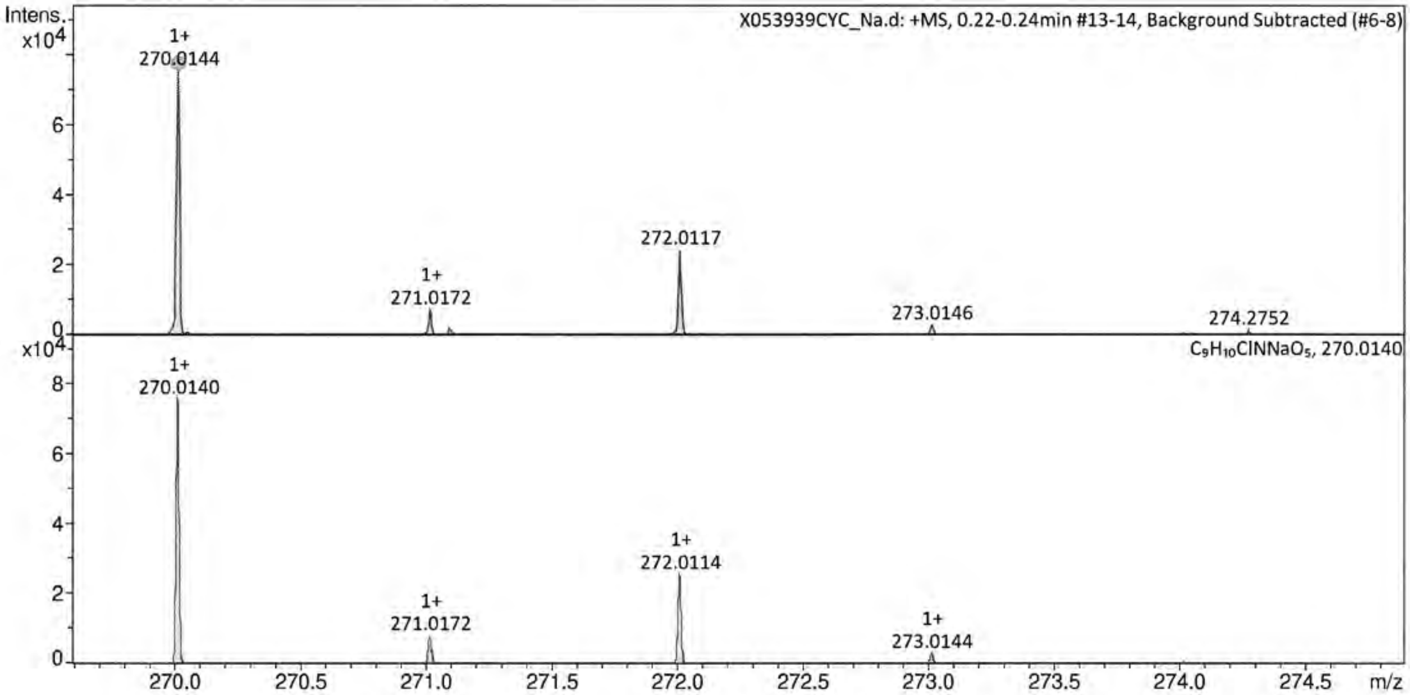

Meas. m/z	z	#	Ion Formula	m/z	err [ppm]	mSigma	rdb	e⁻ Conf
141.010303	1+	1	C7H6ClO	141.010169	-1.0	5.1	5.0	even
149.059909	1+	1	C9H9O2	149.059706	-1.4	27.1	6.0	even
184.028838	1+	1	C9H9ClO2	184.028559	-1.5	25.4	5.5	odd
230.021837	1+	1	C9H9ClNO4	230.021462	-1.6	6.6	6.0	even
270.014356	1+	1	C9H10ClNNaO5	270.013971	-1.4	9.4	5.0	even

Institut de Chimie Organique et Analytique
UMR 7311 - Université d'Orléans - CNRS
BP 6759 - rue de Chartres - F-45067 ORLEANS Cedex 2
cyril.colas@univ-orleans.fr
+33 (0)2 38 49 46 61

Spectrométrie de Masse

Demande d'analyse HRMS

High-Resolution Mass Spectrometry analysis request form

Demandeur (Nom+Prénom) : Sava Alexandru (*Submitter's name*) : AS	Tél : (*Phone*)	Mail : (*E-mail*)
Nom de l'échantillon : AS 306 (*Sample name*) **4d**	Confirmation : ☑ Structure connue (Known structure)	Identification : ☐ Structure inconnue (Unknown structure)
Formule brute : $C_9H_9Cl_2NO_5$ (*Molecular formula*)	Structure : 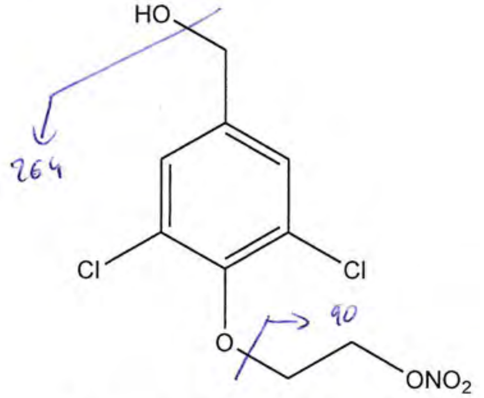 Chemical Formula: $C_9H_9Cl_2NO_5$ Molecular Weight: 282.07 (*Chemical structure*)	
Masse moléculaire : 282,07 (*Molecular weight*)		
Solvant conseillé : CH_3OH (*Recommended solvent*)		
Concentration ou masse : (*Concentration or weight*) 1 mg / 1mL		
Remarques (conservation, solvants déconseillés, toxicité…) : (*Notes: storage, sample handling precautions, toxicity …*)		
Responsable : Sylvain Routier (*Supervisor*)	Date : 06.03.2019 (*Date*)	Bon pour accord : (*Signed as agreed*)

Partie réservée au service (*For MS facility use only*)		
Position sur le rack : (*Position on the rack*) GE1	Nom du fichier : (*File name*) X048797CYC	Date : (*Date*) 13/03/2019
Remarques : (*Comments and suggestions*)		

Plate-forme de Spectrométrie de Masse Haute Résolution – Fédération de Recherche Physique et Chimie du Vivant (FR2708 : CBM/ICOA)

Cyril Colas – Plate-forme HRMS
Institut de Chimie Organique et Analytique
Université d'Orléans – UFR Sciences
Rue de Chartres – BP 6759 – F-45067 ORLEANS Cedex 2
cyril.colas@univ-orleans.fr – +33(0)2 38 49 46 61

Fédération de Recherche Physique et Chimie du Vivant (FR2708 : CBM/ICOA)
Plate-forme de Spectrométrie de Masse Haute Résolution

HRAM

Analysis Info

Sample Name	**AS306**	Acquisition Date	13/03/2019 21:26:44
Analysis Name	X048797CYC_Na.d	Instrument / Ser#	maXis 255552.00086
		Method	positif-6.m

Acquisition Parameter

Source Type	ESI	Ion Polarity	Positive	Set Nebulizer	0.6 Bar
Scan Begin	50 m/z	Set Capillary	4500 V	Set Dry Heater	200 °C
Scan End	2500 m/z	Set Collision Cell RF	1800.0 Vpp	Set Dry Gas	7.0 l/min

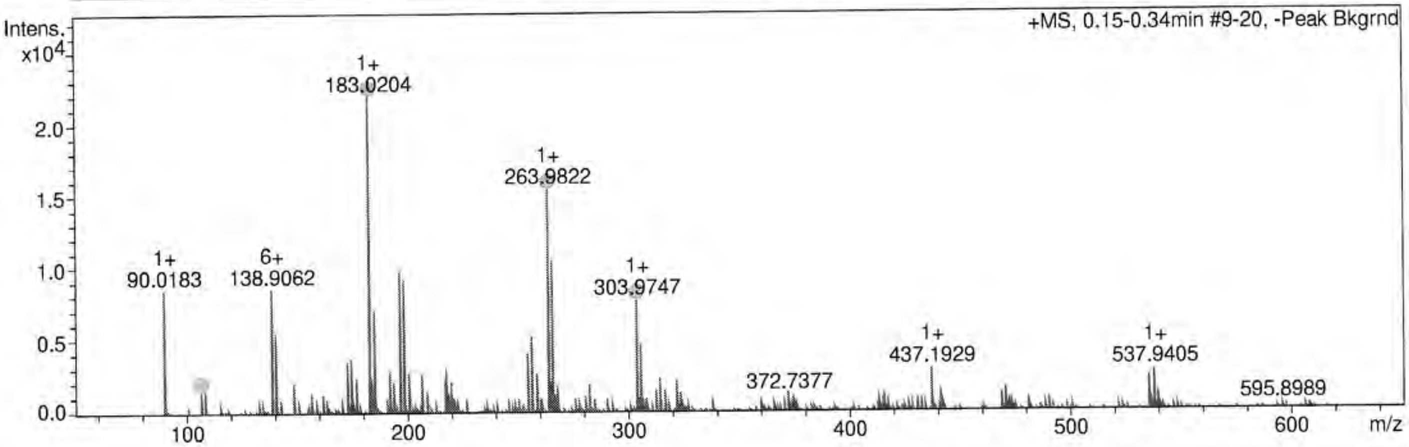

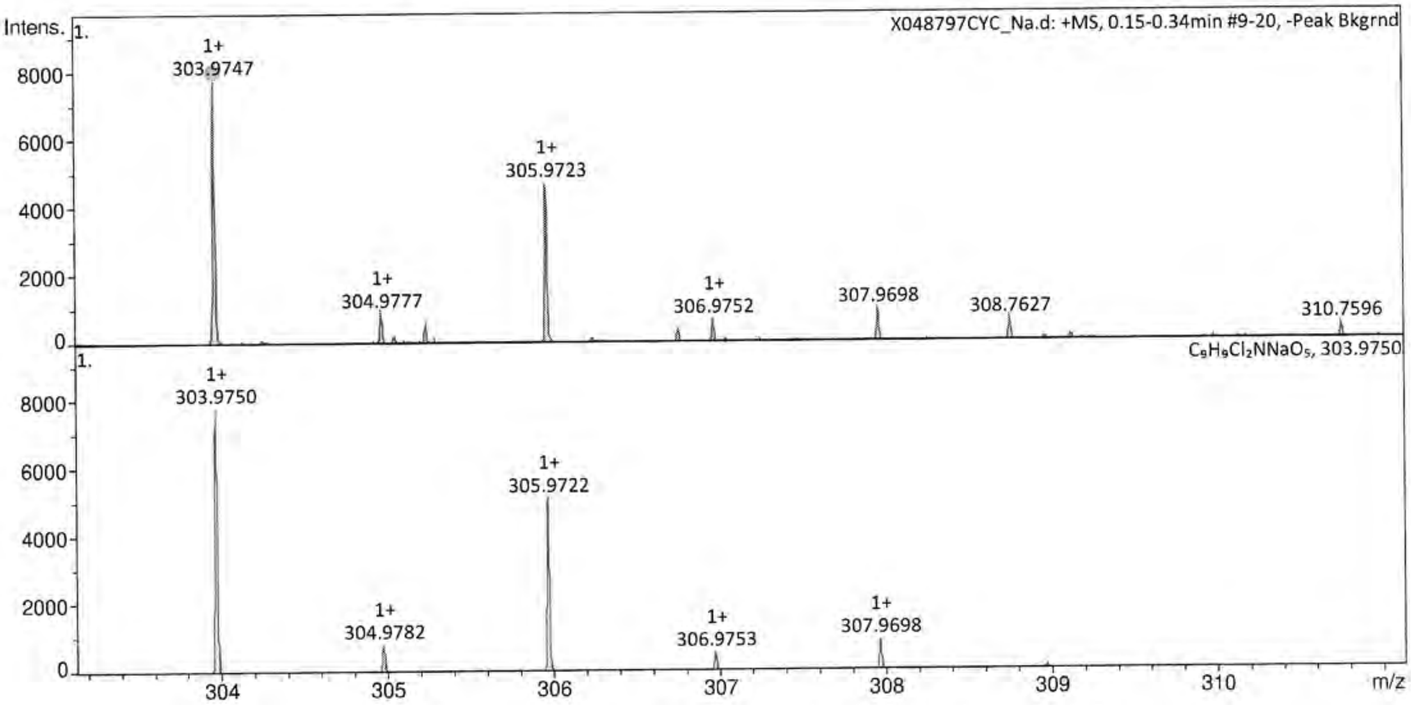

Meas. m/z	z	#	Ion Formula	m/z	err [ppm]	mSigma	rdb	e⁻ Conf
90.018341	1+	1	C2H4NO3	90.018569	2.5	7.0	2.0	even
106.948781	1+	1	C2H4Br	106.949089	2.9	21.9	1.0	even
183.020352	1+	1	C9H8ClO2	183.020734	2.1	5.2	6.0	even
263.982218	1+	1	C9H8Cl2NO4	263.982490	1.0	19.7	6.0	even
303.974711	1+	1	C9H9Cl2NNaO5	303.974998	0.9	22.4	5.0	even

Institut de Chimie Organique et Analytique
UMR 7311 - Université d'Orléans - CNRS
BP 6759 - rue de Chartres - F-45067 ORLEANS Cedex 2
cyril.colas@univ-orleans.fr
+33 (0)2 38 49 46 61

Spectrométrie de Masse

Demande d'analyse HRMS

High-Resolution Mass Spectrometry analysis request form

Demandeur (Nom+Prénom) : Sava Alexandru<br>(*Submitter's name*) : AS	Tél :<br>(*Phone*)	Mail :<br>(*E-mail*)

Nom de l'échantillon : AS 373
(*Sample name*)

4e

Confirmation : ☑ Structure connue (Known structure)

Identification : ☐ Structure inconnue (Unknown structure)

Formule brute : $C_9H_{10}N_2O_7$
(*Molecular formula*)

Masse moléculaire : 258,19
(*Molecular weight*)

Solvant conseillé : CH_3OH
(*Recommended solvent*)

Concentration ou masse :
(*Concentration or weight*)
1 mg / 1mL

Structure :(*Chemical structure)*

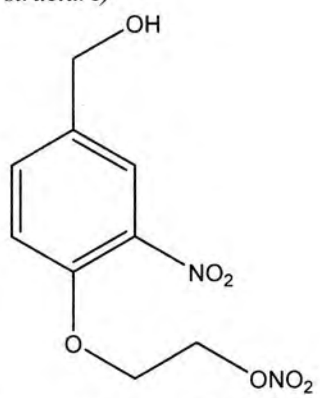

Chemical Formula: $C_9H_{10}N_2O_7$
Molecular Weight: 258.19

Remarques (conservation, solvants déconseillés, toxicité…) :
(*Notes: storage, sample handling precautions, toxicity …*)

Responsable : Sylvain Routier<br>(*Supervisor*)	Date : 10.12.2019<br>(*Date*)	Bon pour accord :<br>(*Signed as agreed*)

Partie réservée au service
(For MS facility use only)

Position sur le rack :<br>(*Position on the rack*) GB6	Nom du fichier :<br>(*File name*) X053937CYC	Date :<br>(*Date*) 10/12/2019

Remarques :
(*Comments and suggestions*)

Cyril Colas – Plate-forme HRMS
Institut de Chimie Organique et Analytique
Université d'Orléans – UFR Sciences
Rue de Chartres – BP 6759 – F-45067 ORLEANS Cedex 2
cyril.colas@univ-orleans.fr – +33(0)2 38 49 46 61

Plate-forme de Spectrométrie de Masse Haute Résolution – Fédération de Recherche Physique et Chimie du Vivant (FR2708 : CBM/ICOA)

Fédération de Recherche Physique et Chimie du Vivant (FR2708 : CBM/ICOA)
Plate-forme de Spectrométrie de Masse Haute Résolution

HRAM

Analysis Info

Sample Name	**AS373**	Acquisition Date	11/12/2019 17:37:42
Analysis Name	X053937CYC.d	Instrument / Ser#	maXis 255552.00086
		Method	Positif.m

Acquisition Parameter

Source Type	ESI	Ion Polarity	Positive	Set Nebulizer	0.6 Bar
Scan Begin	50 m/z	Set Capillary	4500 V	Set Dry Heater	200 °C
Scan End	2500 m/z	Set Collision Cell RF	1800.0 Vpp	Set Dry Gas	7.0 l/min

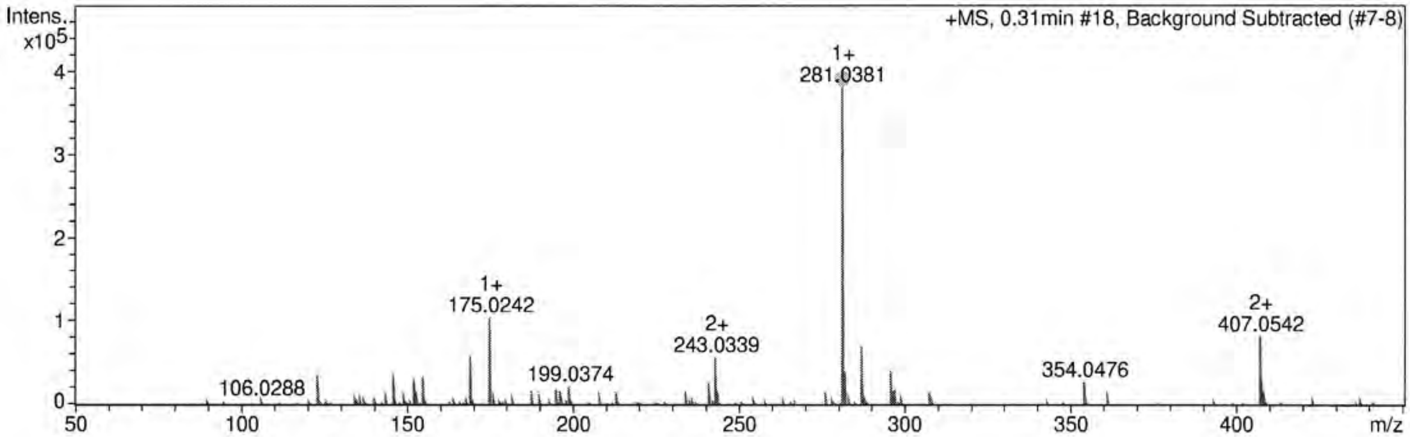

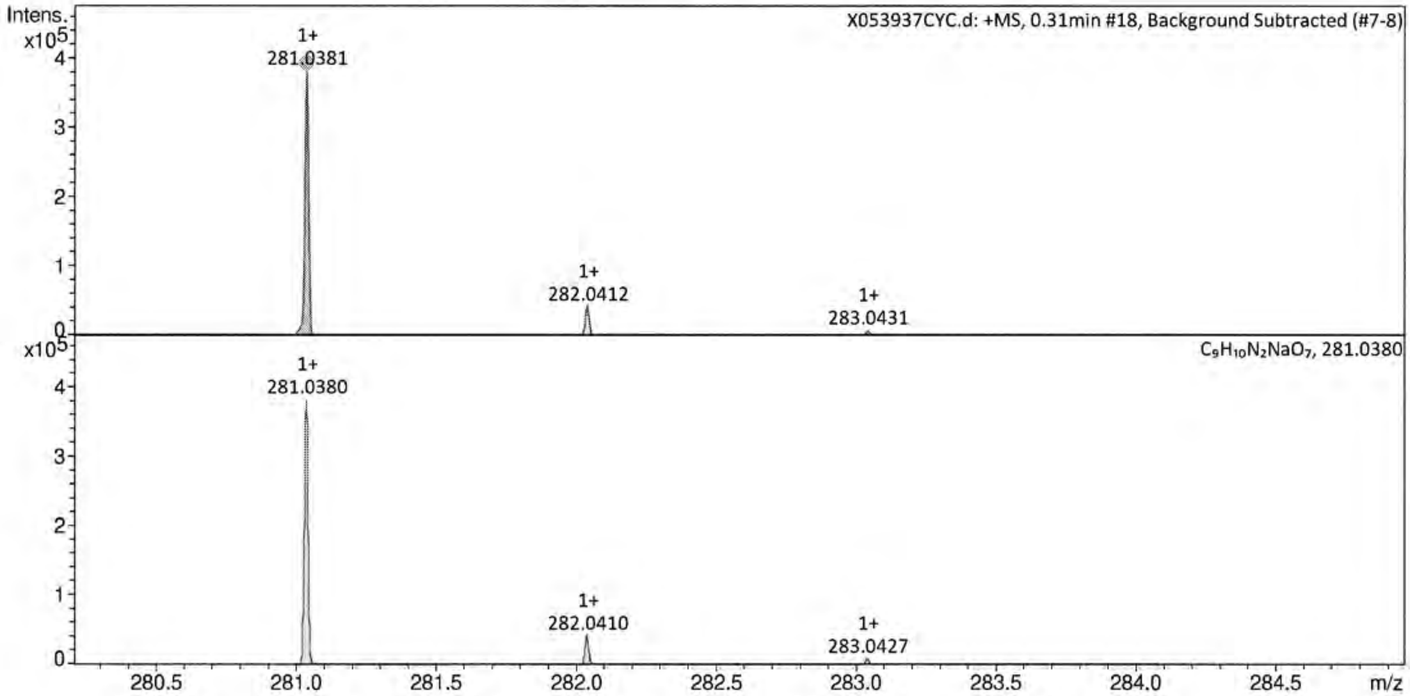

Meas. m/z	z	#	Ion Formula	m/z	err [ppm]	mSigma	rdb	e⁻ Conf
281.038115	1+	1	C9H10N2NaO7	281.038021	-0.3	1.1	6.0	even

Spectrométrie de Masse

Demande d'analyse HRMS

High-Resolution Mass Spectrometry analysis request form

Demandeur (Nom+Prénom) : Sava Alexandru
(*Submitter's name*) : AS

Tél :
(*Phone*)

Mail :
(*E-mail*)

Nom de l'échantillon : AS 311
(*Sample name*) **4f**

Confirmation :
☑ Structure connue
(Known structure)

Identification :
☐ Structure inconnue
(Unknown structure)

Formule brute : $C_{10}H_{13}NO_6$
(*Molecular formula*)

Masse moléculaire : 243,22
(*Molecular weight*)

Solvant conseillé : CH_3OH
(*Recommended solvent*)

Concentration ou masse :
(*Concentration or weight*)
1 mg / 1mL

Structure :

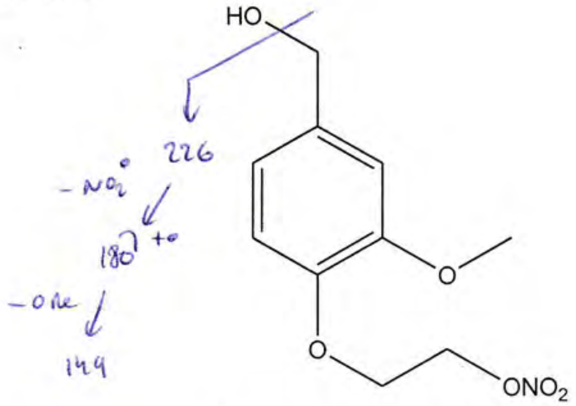

(*Chemical structure*)

Chemical Formula: $C_{10}H_{13}NO_6$
Molecular Weight: 243.22

Remarques (conservation, solvants déconseillés, toxicité…) :
(*Notes: storage, sample handling precautions, toxicity …*)

Responsable : Sylvain Routier
(*Supervisor*)

Date : 06.03.2019
(*Date*)

Bon pour accord :
(*Signed as agreed*)

Partie réservée au service
(For MS facility use only)

Position sur le rack : (*Position on the rack*)	Nom du fichier : (*File name*)	Date : (*Date*)
BB12	X048617CYC	06/03/2019

Remarques :
(*Comments and suggestions*)

Plate-forme de Spectrométrie de Masse Haute Résolution – Fédération de Recherche Physique et Chimie du Vivant (FR2708 : CBM/ICOA)

Cyril Colas – Plate-forme HRMS
Institut de Chimie Organique et Analytique
Université d'Orléans – UFR Sciences
Rue de Chartres – BP 6759 – F-45067 ORLEANS Cedex 2
cyril.colas@univ-orleans.fr – +33(0)2 38 49 46 61

Fédération de Recherche Physique et Chimie du Vivant (FR2708 : CBM/ICOA)
Plate-forme de Spectrométrie de Masse Haute Résolution

HRAM

Analysis Info

Sample Name	**AS311**	Acquisition Date	06/03/2019 18:28:41
Analysis Name	X048617CYC.d	Instrument / Ser#	maXis 255552.00086
		Method	Positif.m

Acquisition Parameter

Source Type	ESI	Ion Polarity	Positive	Set Nebulizer	0.6 Bar
Scan Begin	50 m/z	Set Capillary	4500 V	Set Dry Heater	200 °C
Scan End	2500 m/z	Set Collision Cell RF	1800.0 Vpp	Set Dry Gas	7.0 l/min

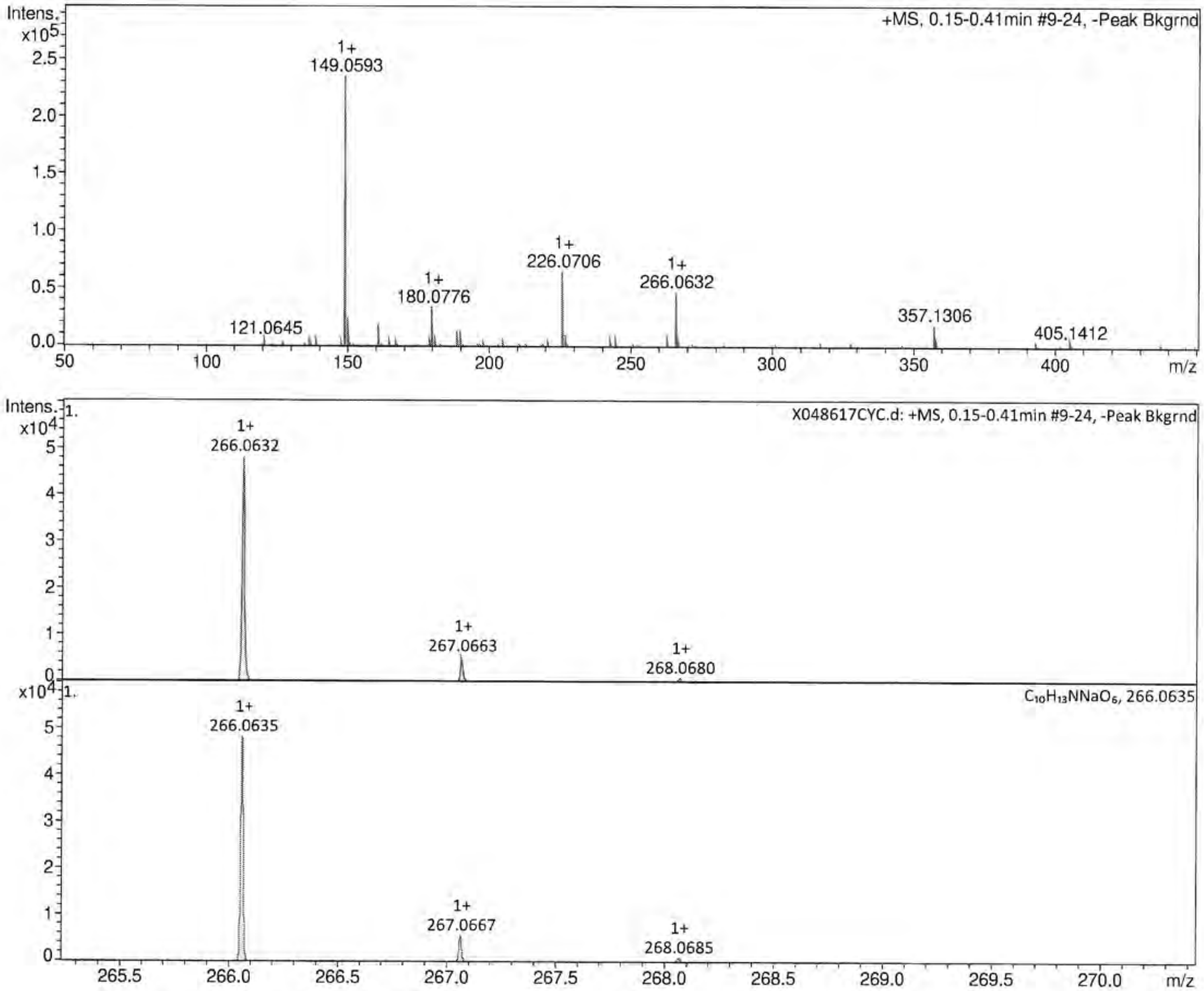

Meas. m/z	z	#	Ion Formula	m/z	err [ppm]	mSigma	rdb	e⁻ Conf
149.059260	1+	1	C9H9O2	149.059706	3.0	4.8	6.0	even
180.077642	1+	1	C10H12O3	180.078096	2.5	36.8	5.5	odd
226.070622	1+	1	C10H12NO5	226.070999	1.7	6.8	6.0	even
266.063250	1+	1	C10H13NNaO6	266.063508	1.0	3.7	5.0	even

Institut de Chimie Organique et Analytique
UMR 7311 - Université d'Orléans - CNRS
BP 6759 - rue de Chartres - F-45067 ORLEANS Cedex 2
cyril.colas@univ-orleans.fr
+33 (0)2 38 49 46 61

printed: 06/03/2019 20:22:48

Spectrométrie de Masse

Demande d'analyse HRMS

High-Resolution Mass Spectrometry analysis request form

Demandeur (Nom+Prénom) : Sava Alexandru<br>(*Submitter's name*) : AS	Tél :<br>(*Phone*)	Mail :<br>(*E-mail*)
Nom de l'échantillon : AS 389<br>(*Sample name*) **5b**	Confirmation :<br>☑ Structure connue<br>(Known structure)	Identification :<br>☐ Structure inconnue<br>(Unknown structure)

Formule brute : $C_9H_9ClFNO_4$<br>(*Molecular formula*)	Structure :(*Chemical structure*)
Masse moléculaire : 249,62<br>(*Molecular weight*)	
Solvant conseillé : CH_3OH<br>(*Recommended solvent*)	
Concentration ou masse :<br>(*Concentration or weight*)<br>1 mg / 1mL	

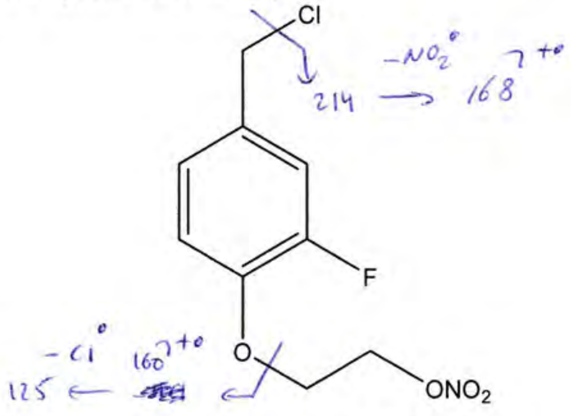

Chemical Formula: $C_9H_9ClFNO_4$
Molecular Weight: 249.62

Remarques (conservation, solvants déconseillés, toxicité…) :
(*Notes: storage, sample handling precautions, toxicity …*)

Responsable : Sylvain Routier<br>(*Supervisor*)	Date : 10.12.2019<br>(*Date*)	Bon pour accord :<br>(*Signed as agreed*)

Partie réservée au service
(For MS facility use only)

Position sur le rack :<br>(*Position on the rack*) GC9	Nom du fichier :<br>(*File name*) X053952CYC	Date :<br>(*Date*) 10/12/2019

Remarques :
(*Comments and suggestions*)

Cyril Colas – Plate-forme HRMS
Institut de Chimie Organique et Analytique
Université d'Orléans – UFR Sciences
Rue de Chartres – BP 6759 – F-45067 ORLEANS Cedex 2
cyril.colas@univ-orleans.fr – +33(0)2 38 49 46 61

Plate-forme de Spectrométrie de Masse Haute Résolution – Fédération de Recherche Physique et Chimie du Vivant (FR2708 : CBM/ICOA)

Fédération de Recherche Physique et Chimie du Vivant (FR2708 : CBM/ICOA)
Plate-forme de Spectrométrie de Masse Haute Résolution

HRAM

Analysis Info

Sample Name	**AS389**	Acquisition Date	11/12/2019 20:32:43
Analysis Name	X053952CYC_Na.d	Instrument / Ser#	maXis 255552.00086
		Method	Positif.m

Acquisition Parameter

Source Type	ESI	Ion Polarity	Positive	Set Nebulizer	0.6 Bar
Scan Begin	50 m/z	Set Capillary	4500 V	Set Dry Heater	200 °C
Scan End	2500 m/z	Set Collision Cell RF	1800.0 Vpp	Set Dry Gas	7.0 l/min

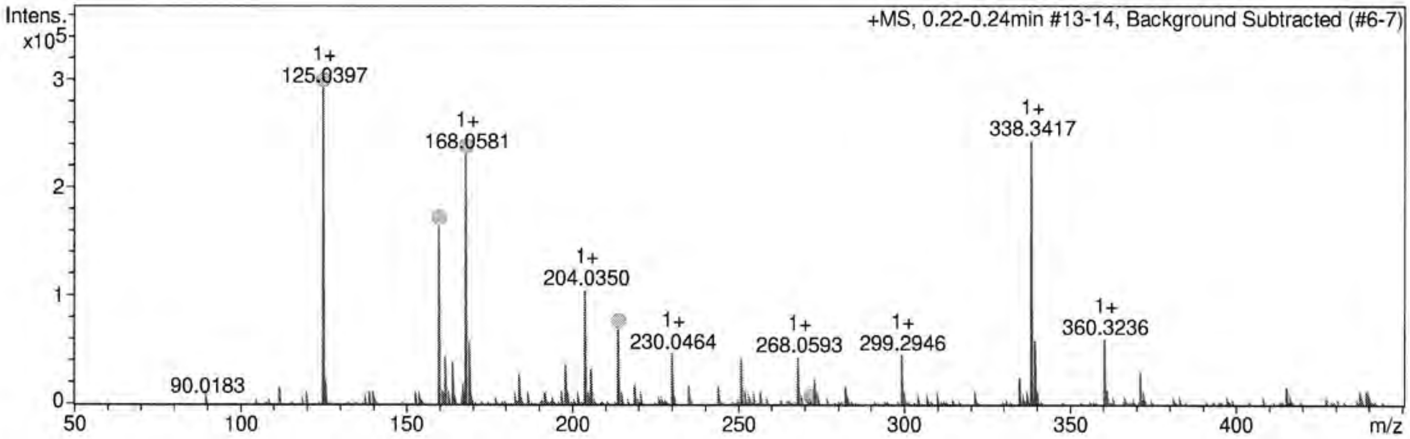

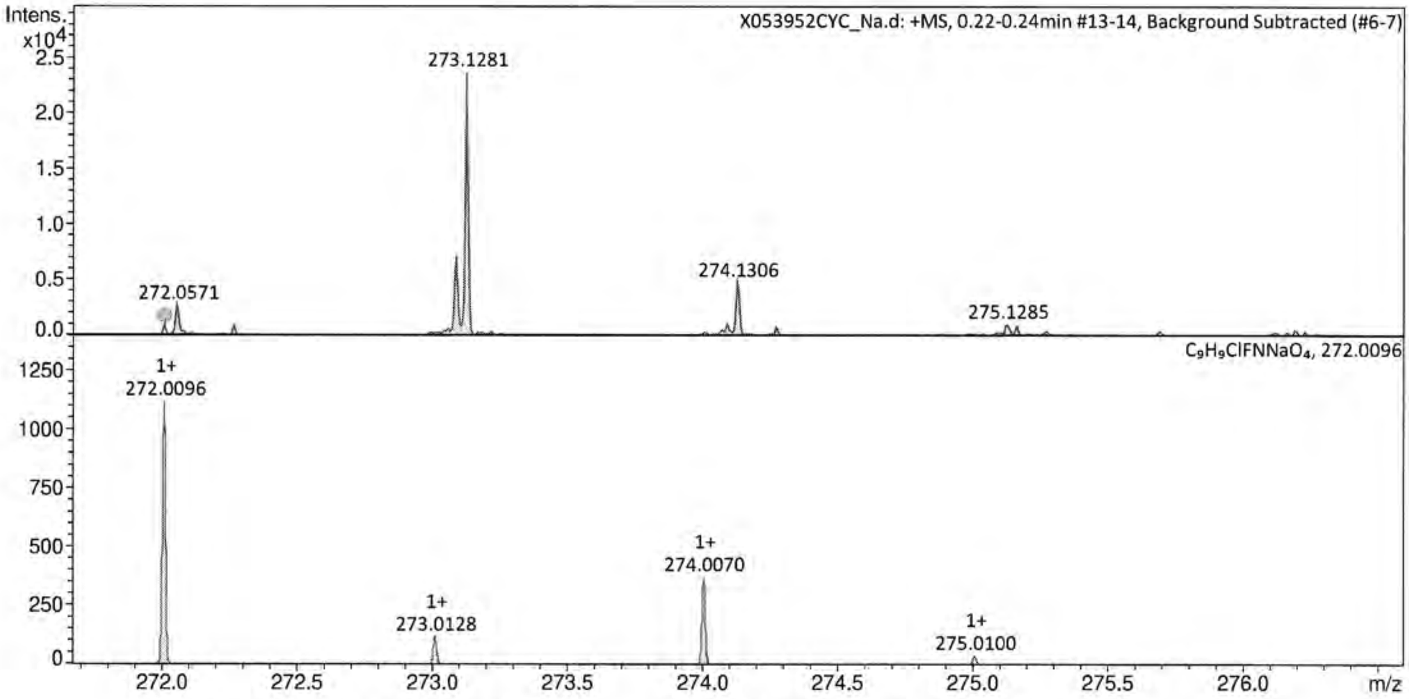

Meas. m/z	z	#	Ion Formula	m/z	err [ppm]	mSigma	rdb	e⁻ Conf
125.039695	1+	1	C7H6FO	125.039719	0.2	0.8	5.0	even
160.008665	1+	1	C7H6ClFO	160.008572	-0.6	23.9	4.5	odd
168.058121	1+	1	C9H9FO2	168.058109	-0.1	88.6	5.5	odd
214.051281	1+	1	C9H9FNO4	214.051012	-1.3	3.6	6.0	even
272.009483	1+	1	C9H9ClFNNaO4	272.009634	0.6	n.a.	5.0	even

Institut de Chimie Organique et Analytique
UMR 7311 - Université d'Orléans - CNRS
BP 6759 - rue de Chartres - F-45067 ORLEANS Cedex 2
cyril.colas@univ-orleans.fr
+33 (0)2 38 49 46 61

Spectrométrie de Masse

Demande d'analyse HRMS

High-Resolution Mass Spectrometry analysis request form

Demandeur (Nom+Prénom) : Sava Alexandru<br>(*Submitter's name*) : AS	Tél :<br>(*Phone*)	Mail :<br>(*E-mail*)
Nom de l'échantillon : AS 380<br>(*Sample name*) **5e**	Confirmation :<br>☑ Structure connue<br>(Known structure)	Identification :<br>☐ Structure inconnue<br>(Unknown structure)

Formule brute : $C_9H_9ClN_2O_6$
(*Molecular formula*)

Masse moléculaire : 276,63
(*Molecular weight*)

Solvant conseillé : CH_3OH
(*Recommended solvent*)

Concentration ou masse :
(*Concentration or weight*)
1 mg / 1mL

Structure :(*Chemical structure*)

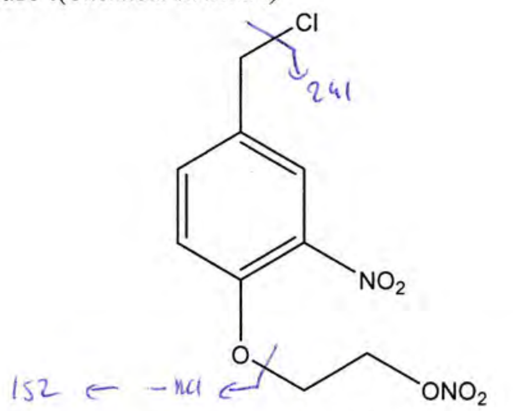

Chemical Formula: $C_9H_9ClN_2O_6$
Molecular Weight: 276.63

Remarques (conservation, solvants déconseillés, toxicité…) :
(*Notes: storage, sample handling precautions, toxicity …*)

Responsable : Sylvain Routier<br>(*Supervisor*)	Date : 10.12.2019<br>(*Date*)	Bon pour accord :<br>(*Signed as agreed*)

Partie réservée au service
(For MS facility use only)

Position sur le rack :<br>(*Position on the rack*) GB12	Nom du fichier :<br>(*File name*) X053943CYC	Date :<br>(*Date*) 10/12/2019

Remarques :
(*Comments and suggestions*)

Cyril Colas – Plate-forme HRMS
Institut de Chimie Organique et Analytique
Université d'Orléans – UFR Sciences
Rue de Chartres – BP 6759 – F-45067 ORLEANS Cedex 2
cyril.colas@univ-orleans.fr – +33(0)2 38 49 46 61

Plate-forme de Spectrométrie de Masse Haute Résolution – Fédération de Recherche Physique et Chimie du Vivant (FR2708 : CBM/ICOA)

Fédération de Recherche Physique et Chimie du Vivant (FR2708 : CBM/ICOA)
Plate-forme de Spectrométrie de Masse Haute Résolution

HRAM

Analysis Info

Sample Name	**AS380**	Acquisition Date	11/12/2019 17:46:43
Analysis Name	X053943CYC.d	Instrument / Ser#	maXis 255552.00086
		Method	Positif.m

Acquisition Parameter

Source Type	ESI	Ion Polarity	Positive	Set Nebulizer	0.6 Bar
Scan Begin	50 m/z	Set Capillary	4500 V	Set Dry Heater	200 °C
Scan End	2500 m/z	Set Collision Cell RF	1800.0 Vpp	Set Dry Gas	7.0 l/min

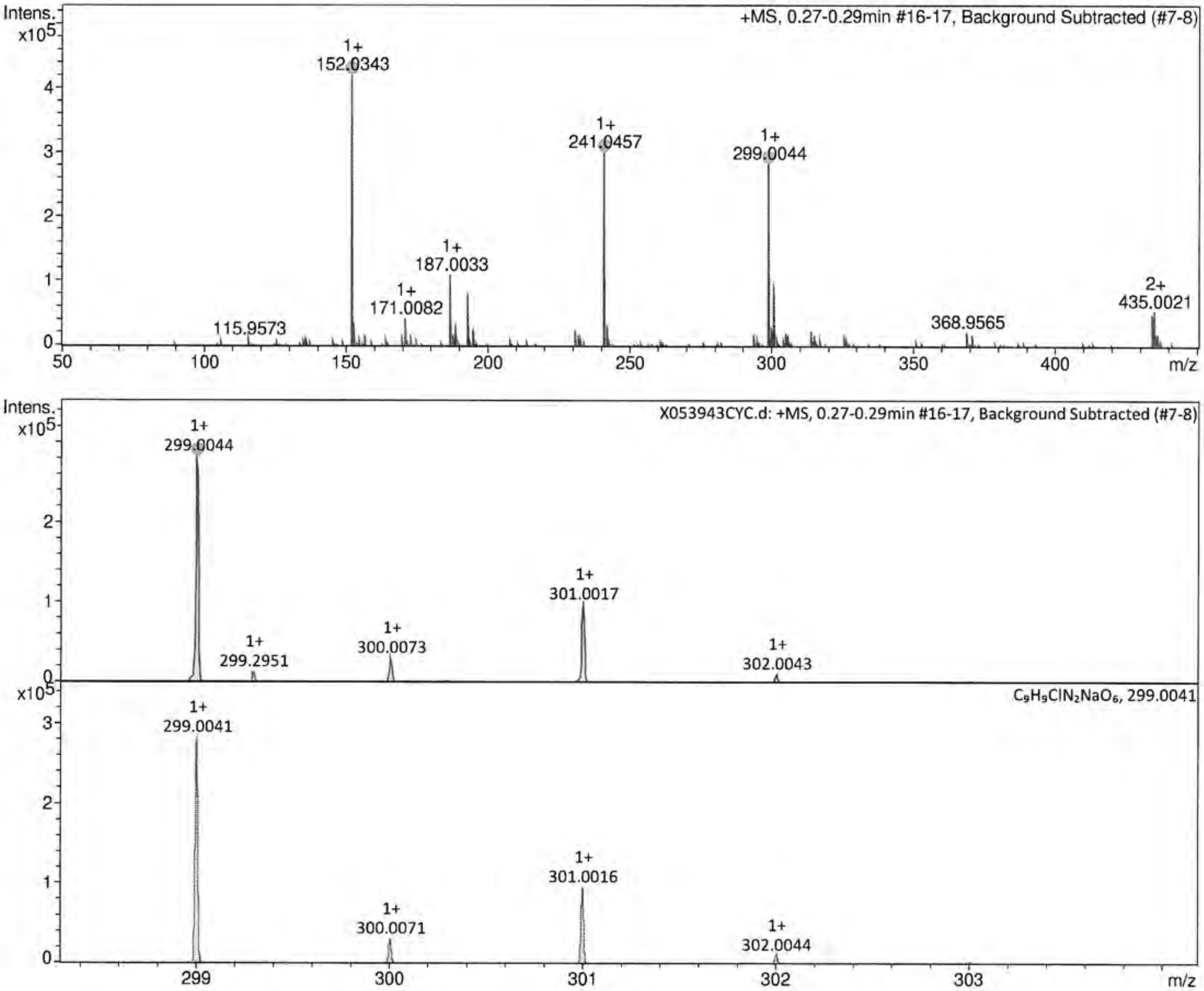

Meas. m/z	z	#	Ion Formula	m/z	err [ppm]	mSigma	rdb	e⁻ Conf
152.034253	1+	1	C7H6NO3	152.034219	-0.2	4.2	6.0	even
241.045718	1+	1	C9H9N2O6	241.045512	-0.9	0.5	7.0	even
299.004402	1+	1	C9H9ClN2NaO6	299.004134	-0.9	4.2	6.0	even

Institut de Chimie Organique et Analytique
UMR 7311 - Université d'Orléans - CNRS
BP 6759 - rue de Chartres - F-45067 ORLEANS Cedex 2
cyril.colas@univ-orleans.fr
+33 (0)2 38 49 46 61

Spectrométrie de Masse

Demande d'analyse HRMS

High-Resolution Mass Spectrometry analysis request form

Demandeur (Nom+Prénom) : Sava Alexandru<br>(*Submitter's name*) : AS	Tél :<br>(*Phone*)	Mail :<br>(*E-mail*)
Nom de l'échantillon : AS 335<br>(*Sample name*) **5h**	Confirmation :<br>☑ Structure connue<br>(Known structure)	Identification :<br>☐ Structure inconnue<br>(Unknown structure)

Formule brute : $C_{11}H_{14}ClNO_6$<br>(*Molecular formula*)	Structure :
Masse moléculaire : 291,68<br>(*Molecular weight*)	
Solvant conseillé : CH_3OH<br>(*Recommended solvent*)	
Concentration ou masse :<br>(*Concentration or weight*)<br>1 mg / 1mL	(*Chemical structure*)

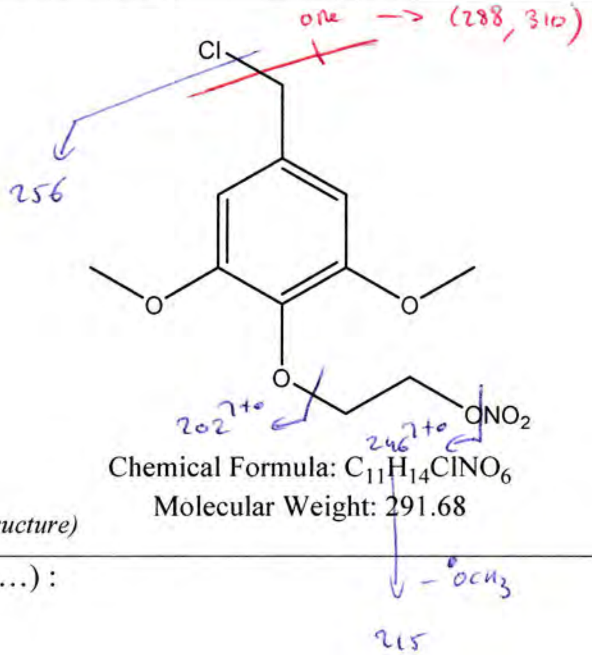

Chemical Formula: $C_{11}H_{14}ClNO_6$
Molecular Weight: 291.68

Remarques (conservation, solvants déconseillés, toxicité…) :
(*Notes: storage, sample handling precautions, toxicity …*)

Responsable : Sylvain Routier<br>(*Supervisor*)	Date : 12.03.2019<br>(*Date*)	Bon pour accord :<br>(*Signed as agreed*)

Partie réservée au service
(For MS facility use only)

Position sur le rack :<br>(*Position on the rack*) GD11	Nom du fichier :<br>(*File name*) X048795CYC	Date :<br>(*Date*) 13/03/2019

Remarques :
(*Comments and suggestions*)

Cyril Colas – Plate-forme HRMS
Institut de Chimie Organique et Analytique
Université d'Orléans – UFR Sciences
Rue de Chartres – BP 6759 – F-45067 ORLEANS Cedex 2
cyril.colas@univ-orleans.fr – +33(0)2 38 49 46 61

Plate-forme de Spectrométrie de Masse Haute Résolution – Fédération de Recherche Physique et Chimie du Vivant (FR2708 : CBM/ICOA)

Analysis Info

Sample Name	**AS335**	Acquisition Date	13/03/2019 19:29:02
Analysis Name	X048795CYC.d	Instrument / Ser#	maXis 255552.00086
		Method	positif-6.m

Acquisition Parameter

Source Type	ESI	Ion Polarity	Positive	Set Nebulizer	0.6 Bar
Scan Begin	50 m/z	Set Capillary	4500 V	Set Dry Heater	200 °C
Scan End	2500 m/z	Set Collision Cell RF	1800.0 Vpp	Set Dry Gas	7.0 l/min

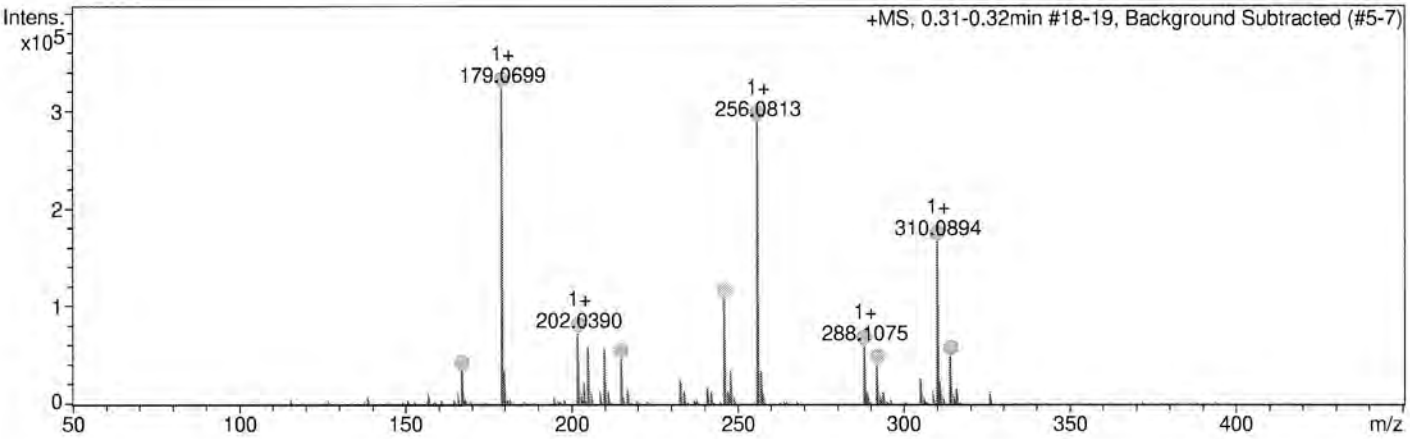

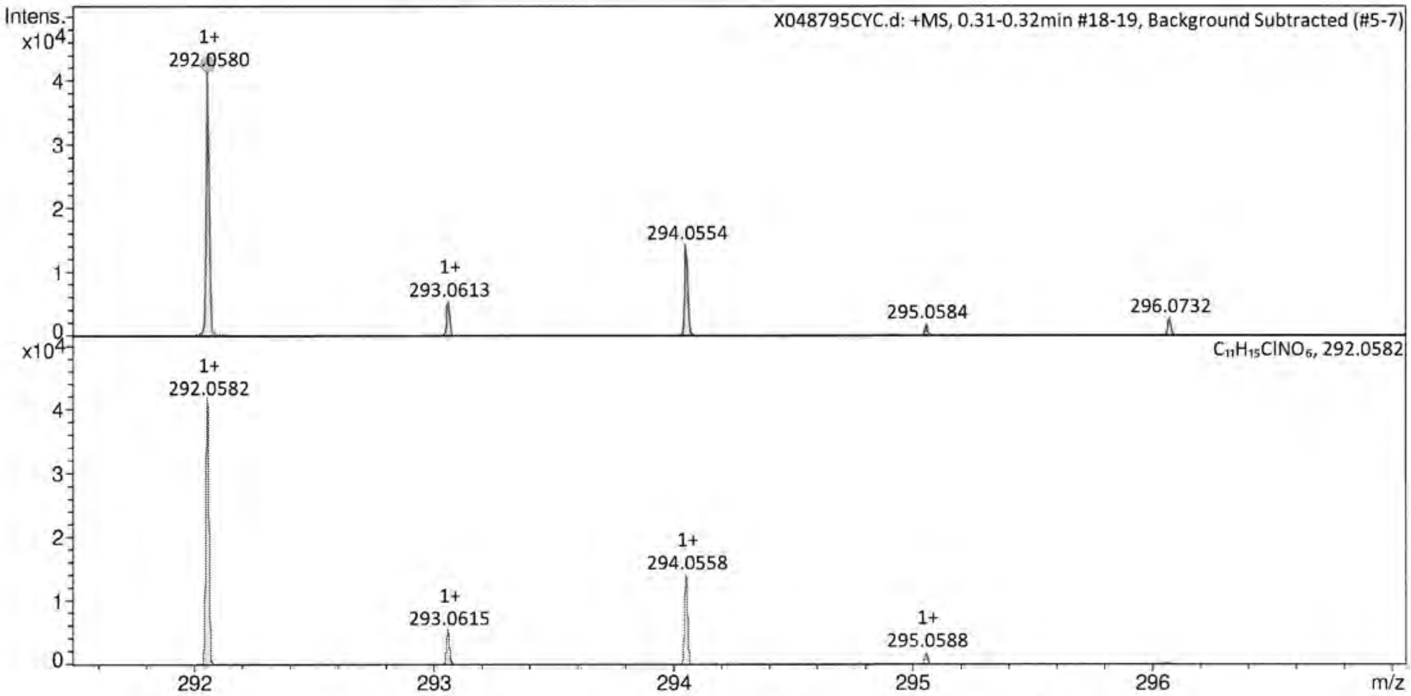

Meas. m/z	z	#	Ion Formula	m/z	err [ppm]	mSigma	rdb	e⁻ Conf
167.069848	1+	1	C9H11O3	167.070271	2.5	4.4	5.0	even
179.069931	1+	1	C10H11O3	179.070271	1.9	1.2	6.0	even
202.038986	1+	1	C9H11ClO3	202.039123	0.7	11.0	4.5	odd
215.046623	1+	1	C10H12ClO3	215.046948	1.5	4.0	5.0	even
246.065056	1+	1	C11H15ClO4	246.065338	1.1	7.6	4.5	odd
256.081307	1+	1	C11H14NO6	256.081564	1.0	3.6	6.0	even
288.107472	1+	1	C12H18NO7	288.107778	1.1	9.5	5.0	even
292.057966	1+	1	C11H15ClNO6	292.058241	0.9	2.0	5.0	even

Meas. m/z	z	#	Ion Formula	m/z	err [ppm]	mSigma	rdb	e⁻ Conf
310.089446	1+	1	$C_{12}H_{17}NNaO_7$	310.089723	0.9	2.1	5.0	even
314.040125	1+	1	$C_{11}H_{14}ClNNaO_6$	314.040186	0.2	14.0	5.0	even

Spectrométrie de Masse

Demande d'analyse HRMS

High-Resolution Mass Spectrometry analysis request form

Demandeur (Nom+Prénom) : Sava Alexandru<br>(*Submitter's name*) : AS	Tél :<br>(*Phone*)	Mail :<br>(*E-mail*)
Nom de l'échantillon : AS 382<br>(*Sample name*)<br>**5k**	Confirmation :<br>☑ Structure connue<br>(Known structure)	Identification :<br>☐ Structure inconnue<br>(Unknown structure)

Formule brute : $C_9H_9ClN_2O_6$<br>(*Molecular formula*)	Structure :(*Chemical structure*)
Masse moléculaire : 276,63<br>(*Molecular weight*)	
Solvant conseillé : CH_3OH<br>(*Recommended solvent*)	
Concentration ou masse :<br>(*Concentration or weight*)<br>1 mg / 1mL	

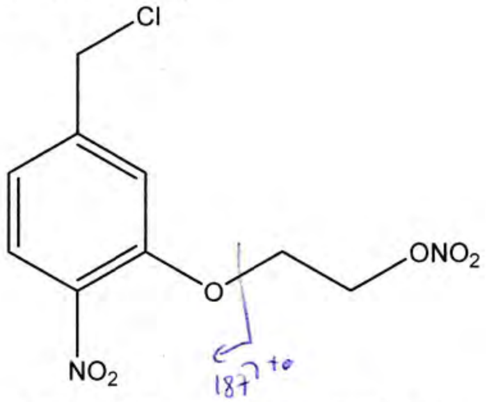

Chemical Formula: $C_9H_9ClN_2O_6$
Molecular Weight: 276.63

Remarques (conservation, solvants déconseillés, toxicité…) :
(*Notes: storage, sample handling precautions, toxicity …*)

Responsable : Sylvain Routier<br>(*Supervisor*)	Date : 10.12.2019<br>(*Date*)	Bon pour accord :<br>(*Signed as agreed*)

Partie réservée au service
(*For MS facility use only*)

Position sur le rack :<br>(*Position on the rack*) GC2	Nom du fichier :<br>(*File name*) X053945CYC	Date :<br>(*Date*) 10/12/2019

Remarques :
(*Comments and suggestions*)

Cyril Colas – Plate-forme HRMS
Institut de Chimie Organique et Analytique
Université d'Orléans – UFR Sciences
Rue de Chartres – BP 6759 – F-45067 ORLEANS Cedex 2
cyril.colas@univ-orleans.fr – +33(0)2 38 49 46 61

Plate-forme de Spectrométrie de Masse Haute Résolution – Fédération de Recherche Physique et Chimie du Vivant (FR2708 : CBM/ICOA)

Fédération de Recherche Physique et Chimie du Vivant (FR2708 : CBM/ICOA)
Plate-forme de Spectrométrie de Masse Haute Résolution

HRAM

Analysis Info

Sample Name	**AS382**	Acquisition Date	11/12/2019 17:49:39
Analysis Name	X053945CYC.d	Instrument / Ser#	maXis 255552.00086
		Method	Positif.m

Acquisition Parameter

Source Type	ESI	Ion Polarity	Positive	Set Nebulizer	0.6 Bar
Scan Begin	50 m/z	Set Capillary	4500 V	Set Dry Heater	200 °C
Scan End	2500 m/z	Set Collision Cell RF	1800.0 Vpp	Set Dry Gas	7.0 l/min

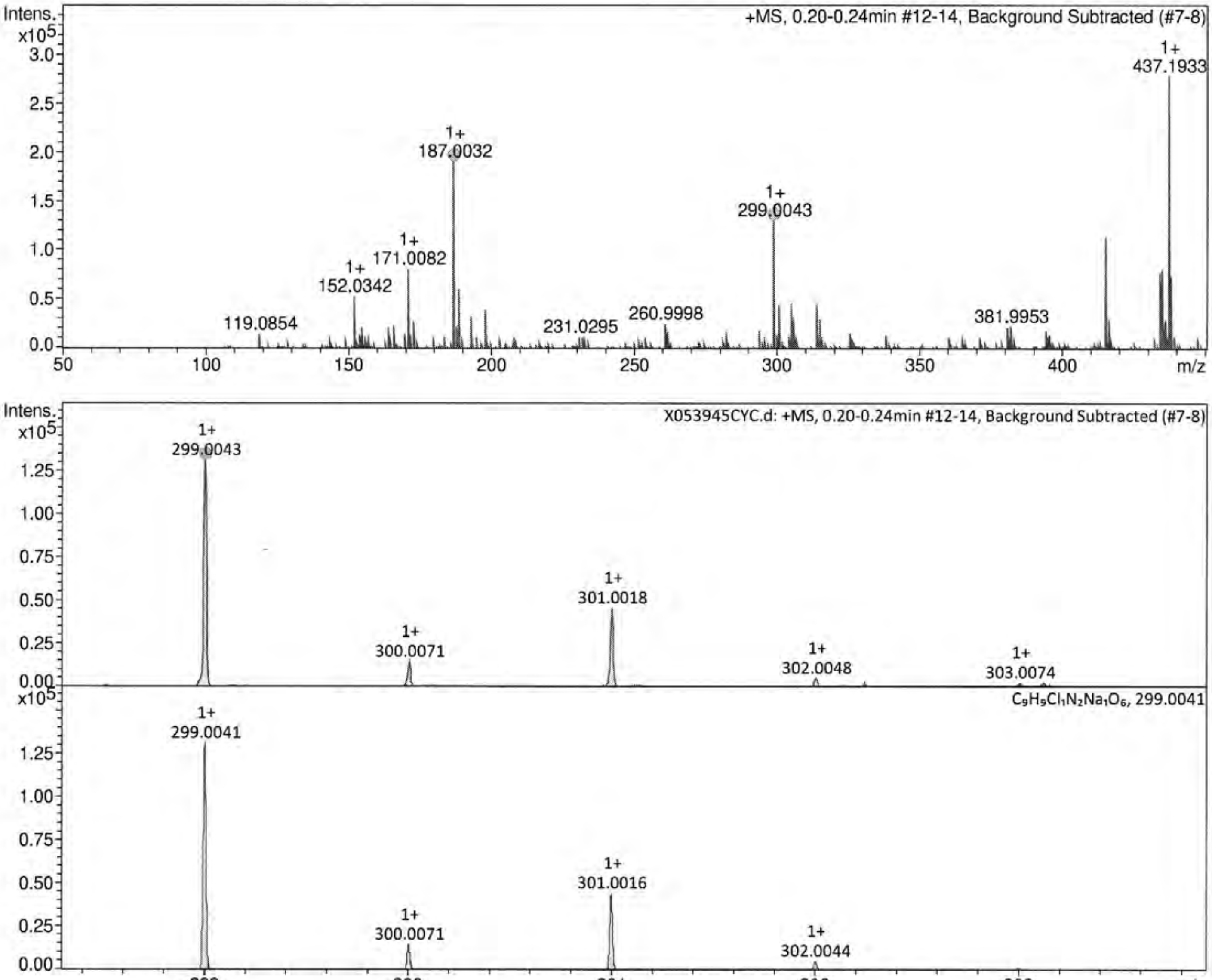

Meas. m/z	z	#	Ion Formula	m/z	err [ppm]	mSigma	rdb	e⁻ Conf
187.003209	1+	1	C7H6ClNO3	187.003072	-0.7	20.5	5.5	odd
299.004346	1+	1	C9H9ClN2NaO6	299.004134	-0.7	5.0	6.0	even

Institut de Chimie Organique et Analytique
UMR 7311 - Université d'Orléans - CNRS
BP 6759 - rue de Chartres - F-45067 ORLEANS Cedex 2
cyril.colas@univ-orleans.fr
+33 (0)2 38 49 46 61

Spectrométrie de Masse

Demande d'analyse HRMS

High-Resolution Mass Spectrometry analysis request form

Demandeur (Nom+Prénom) : Sava Alexandru<br>(*Submitter's name*) : AS	Tél :<br>(*Phone*)	Mail :<br>(*E-mail*)
Nom de l'échantillon : AS 386<br>(*Sample name*)<br>**5l**	Confirmation :<br>☑ Structure connue<br>(Known structure)	Identification :<br>☐ Structure inconnue<br>(Unknown structure)

Formule brute : $C_{10}H_{11}Cl_2NO_5$
(*Molecular formula*)

Masse moléculaire : 296,10
(*Molecular weight*)

Solvant conseillé : CH_3OH
(*Recommended solvent*)

Concentration ou masse :
(*Concentration or weight*)
1 mg / 1mL

Structure :(*Chemical structure*)

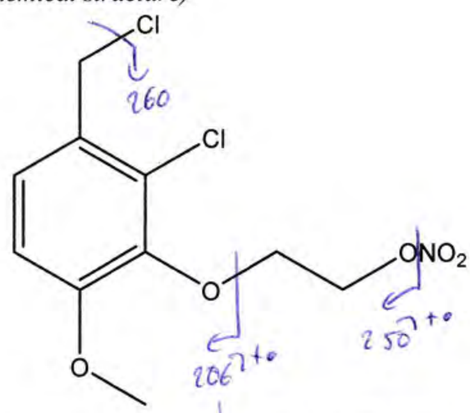

Chemical Formula: $C_{10}H_{11}Cl_2NO_5$
Molecular Weight: 296.10

−Cl°
171

Remarques (conservation, solvants déconseillés, toxicité…) :
(*Notes: storage, sample handling precautions, toxicity …*)

Responsable : Sylvain Routier<br>(*Supervisor*)	Date : 10.12.2019<br>(*Date*)	Bon pour accord :<br>(*Signed as agreed*)

Partie réservée au service
(For MS facility use only)

Position sur le rack :<br>(*Position on the rack*) GC6	Nom du fichier :<br>(*File name*) X053949CYC	Date :<br>(*Date*) 10/12/2019

Remarques :
(*Comments and suggestions*)

Plate-forme de Spectrométrie de Masse Haute Résolution – Fédération de Recherche Physique et Chimie du Vivant (FR2708 : CBM/ICOA)

Cyril Colas – Plate-forme HRMS
Institut de Chimie Organique et Analytique
Université d'Orléans – UFR Sciences
Rue de Chartres – BP 6759 – F-45067 ORLEANS Cedex 2
cyril.colas@univ-orleans.fr – +33(0)2 38 49 46 61

Analysis Info

Sample Name	**AS386**	Acquisition Date	11/12/2019 17:55:38
Analysis Name	X053949CYC.d	Instrument / Ser#	maXis 255552.00086
		Method	Positif.m

Acquisition Parameter

Source Type	ESI	Ion Polarity	Positive	Set Nebulizer	0.6 Bar
Scan Begin	50 m/z	Set Capillary	4500 V	Set Dry Heater	200 °C
Scan End	2500 m/z	Set Collision Cell RF	1800.0 Vpp	Set Dry Gas	7.0 l/min

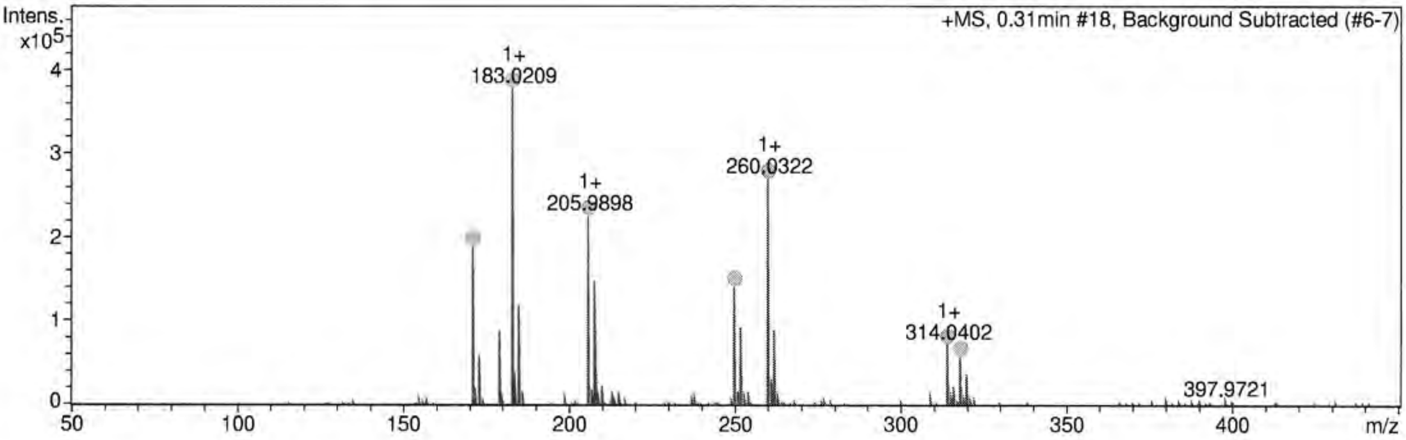

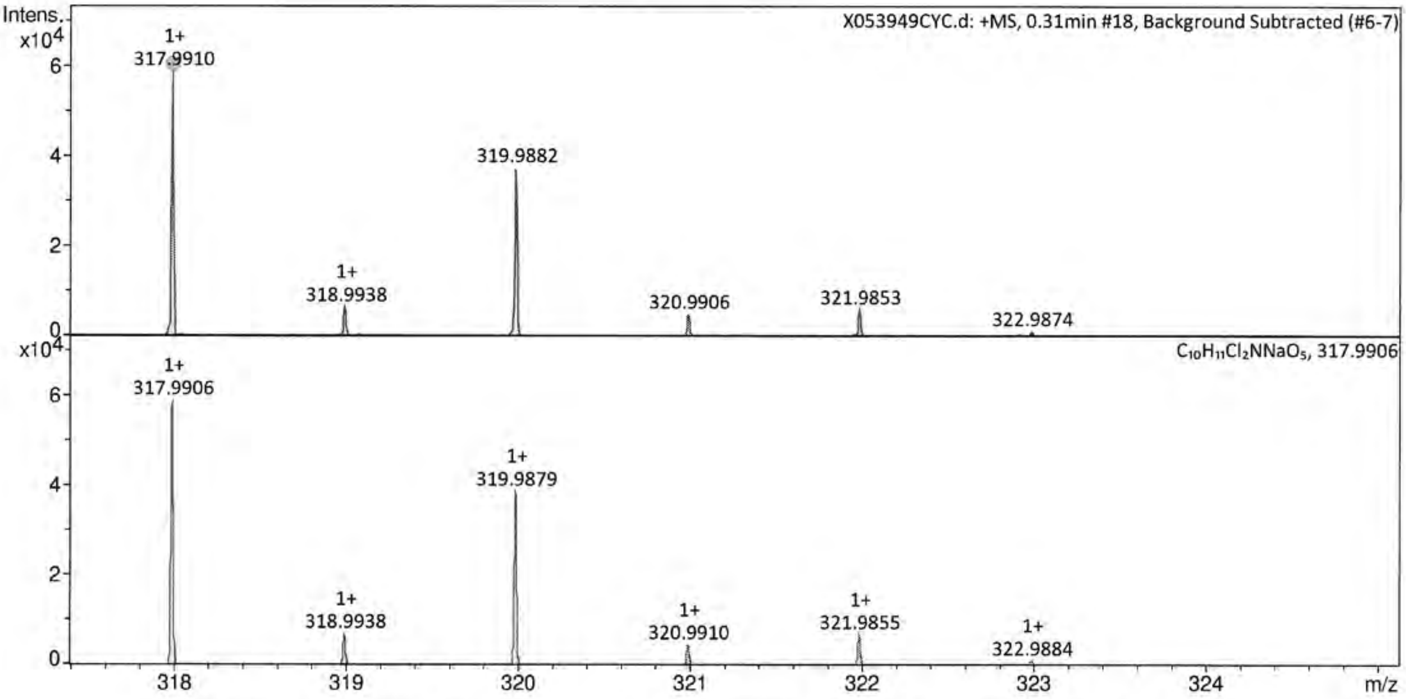

Meas. m/z	z	#	Ion Formula	m/z	err [ppm]	mSigma	rdb	e⁻ Conf
171.020812	1+	1	C8H8ClO2	171.020734	-0.5	7.3	5.0	even
183.020856	1+	1	C9H8ClO2	183.020734	-0.7	6.7	6.0	even
205.989788	1+	1	C8H8Cl2O2	205.989586	-1.0	3.4	4.5	odd
250.016015	1+	1	C10H12Cl2O3	250.015801	-0.9	4.9	4.5	odd
260.032228	1+	1	C10H11ClNO5	260.032027	-0.8	1.4	6.0	even
314.040197	1+	1	C11H14ClNNaO6	314.040186	-0.0	17.4	5.0	even
317.990984	1+	1	C10H11Cl2NNaO5	317.990649	-1.1	11.8	5.0	even

Spectrométrie de Masse

Demande d'analyse HRMS

High-Resolution Mass Spectrometry analysis request form

Demandeur (Nom+Prénom) : Sava Alexandru<br>(*Submitter's name*) : AS	Tél :<br>(*Phone*)	Mail :<br>(*E-mail*)
Nom de l'échantillon : AS 383<br>(*Sample name*) **5m**	Confirmation :<br>☑ Structure connue<br>(Known structure)	Identification :<br>☐ Structure inconnue<br>(Unknown structure)

Formule brute : $C_{10}H_{11}BrClNO_5$
(*Molecular formula*)

Masse moléculaire : 340,55
(*Molecular weight*)

Solvant conseillé : CH_3OH
(*Recommended solvent*)

Concentration ou masse :
(*Concentration or weight*)

1 mg / 1mL

Structure :(*Chemical structure*)

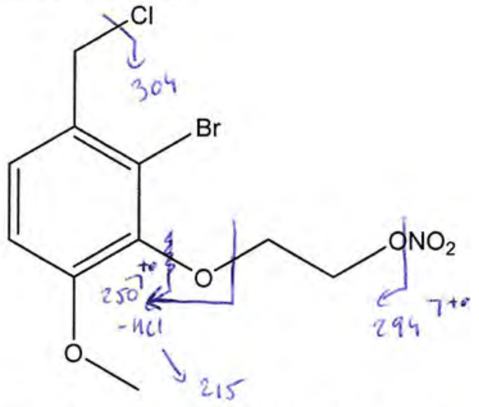

Chemical Formula: $C_{10}H_{11}BrClNO_5$
Molecular Weight: 340.55

Remarques (conservation, solvants déconseillés, toxicité…) :
(*Notes: storage, sample handling precautions, toxicity …*)

Responsable : Sylvain Routier<br>(*Supervisor*)	Date : 10.12.2019<br>(*Date*)	Bon pour accord :<br>(*Signed as agreed*)

Partie réservée au service
(For MS facility use only)

Position sur le rack :<br>(*Position on the rack*) GC3	Nom du fichier :<br>(*File name*) X053946CYC	Date :<br>(*Date*) 10/12/2019

Remarques :
(*Comments and suggestions*)

Plate-forme de Spectrométrie de Masse Haute Résolution – Fédération de Recherche Physique et Chimie du Vivant (FR2708 : CBM/ICOA)

Cyril Colas – Plate-forme HRMS
Institut de Chimie Organique et Analytique
Université d'Orléans – UFR Sciences
Rue de Chartres – BP 6759 – F-45067 ORLEANS Cedex 2
cyril.colas@univ-orleans.fr – +33(0)2 38 49 46 61

Fédération de Recherche Physique et Chimie du Vivant (FR2708 : CBM/ICOA)
Plate-forme de Spectrométrie de Masse Haute Résolution

HRAM

Analysis Info

Sample Name	**AS383**	Acquisition Date	11/12/2019 17:51:08
Analysis Name	X053946CYC.d	Instrument / Ser#	maXis 255552.00086
		Method	Positif.m

Acquisition Parameter

Source Type	ESI	Ion Polarity	Positive	Set Nebulizer	0.6 Bar
Scan Begin	50 m/z	Set Capillary	4500 V	Set Dry Heater	200 °C
Scan End	2500 m/z	Set Collision Cell RF	1800.0 Vpp	Set Dry Gas	7.0 l/min

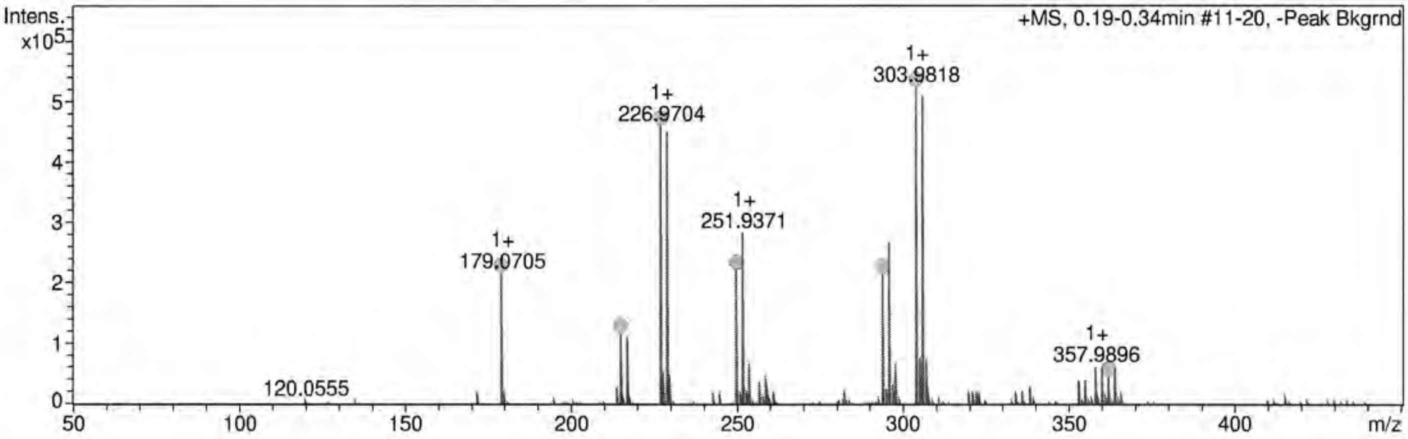

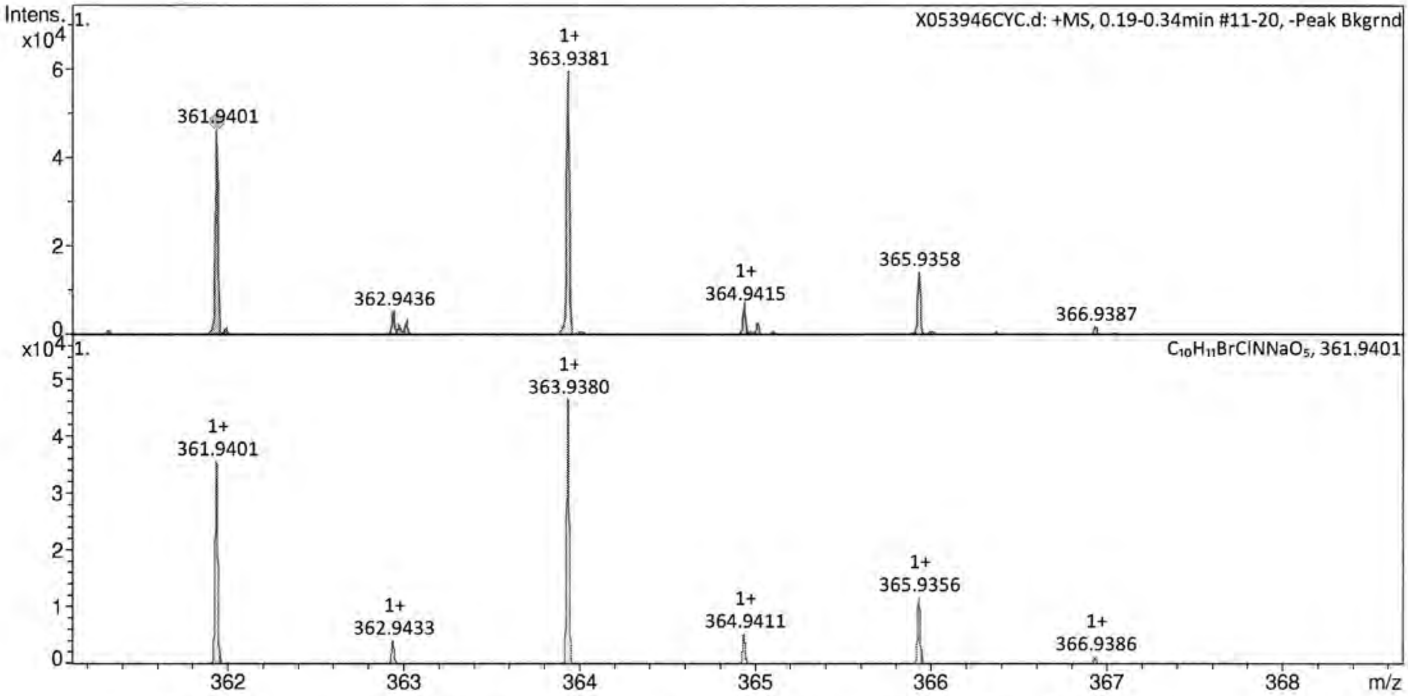

Meas. m/z	z	#	Ion Formula	m/z	err [ppm]	mSigma	rdb	e⁻ Conf
179.070462	1+	1	C10H11O3	179.070271	-1.1	1.7	6.0	even
214.970325	1+	1	C8H8BrO2	214.970218	-0.5	18.4	5.0	even
226.970423	1+	1	C9H8BrO2	226.970218	-0.9	10.6	6.0	even
249.939272	1+	1	C8H8BrClO2	249.939071	-0.8	7.4	4.5	odd
293.965514	1+	1	C10H12BrClO3	293.965285	-0.8	18.8	4.5	odd
303.981839	1+	1	C10H11BrNO5	303.981511	-1.1	24.1	6.0	even
361.940123	1+	1	C10H11BrClNNaO5	361.940133	0.0	8.9	5.0	even

Institut de Chimie Organique et Analytique
UMR 7311 - Université d'Orléans - CNRS
BP 6759 - rue de Chartres - F-45067 ORLEANS Cedex 2
cyril.colas@univ-orleans.fr
+33 (0)2 38 49 46 61

Spectrométrie de Masse

Demande d'analyse HRMS

High-Resolution Mass Spectrometry analysis request form

Demandeur (Nom+Prénom) : Sava Alexandru<br>(*Submitter's name*) : AS	Tél :<br>(*Phone*)	Mail :<br>(*E-mail*)
Nom de l'échantillon : AS 315<br>(*Sample name*) **8a**	Confirmation :<br>☑ Structure connue<br>(Known structure)	Identification :<br>☐ Structure inconnue<br>(Unknown structure)

Formule brute : $C_{29}H_{25}ClN_4O_7S$<br>(*Molecular formula*)	Structure :<br>(*Chemical structure*)
Masse moléculaire : 609,05<br>(*Molecular weight*)	
Solvant conseillé : Acetone<br>(*Recommended solvent*)	
Concentration ou masse :<br>(*Concentration or weight*)<br>1 mg / 1mL	

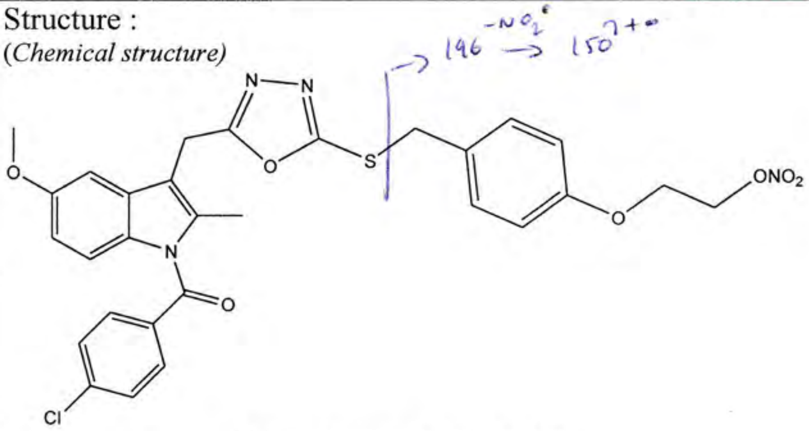

Chemical Formula: $C_{29}H_{25}ClN_4O_7S$
Molecular Weight: 609.05

Remarques (conservation, solvants déconseillés, toxicité…) :
(*Notes: storage, sample handling precautions, toxicity …*)

Responsable : Sylvain Routier<br>(*Supervisor*)	Date : 26.03.2018<br>(*Date*)	Bon pour accord :<br>(*Signed as agreed*)

Partie réservée au service
(*For MS facility use only*)

Position sur le rack :<br>(*Position on the rack*) GE10	Nom du fichier :<br>(*File name*) X048806CYC	Date :<br>(*Date*) 13/03/2019

Remarques :
(*Comments and suggestions*) m/z 333^{2+}, 342^{2+} = $[M+Ca+H_2O]^{2+}$, $[M+Ca+2H_2O]^{2+}$

Plate-forme de Spectrométrie de Masse Haute Résolution – Fédération de Recherche Physique et Chimie du Vivant (FR2708 : CBM/ICOA)

Cyril Colas – Plate-forme HRMS
Institut de Chimie Organique et Analytique
Université d'Orléans – UFR Sciences
Rue de Chartres – BP 6759 – F-45067 ORLEANS Cedex 2
cyril.colas@univ-orleans.fr – +33(0)2 38 49 46 61

Analysis Info

Sample Name	**AS315**	Acquisition Date	13/03/2019 19:46:45
Analysis Name	X048806CYC.d	Instrument / Ser#	maXis 255552.00086
		Method	positif-6.m

Acquisition Parameter

Source Type	ESI	Ion Polarity	Positive	Set Nebulizer	0.6 Bar
Scan Begin	50 m/z	Set Capillary	4500 V	Set Dry Heater	200 °C
Scan End	2500 m/z	Set Collision Cell RF	1800.0 Vpp	Set Dry Gas	7.0 l/min

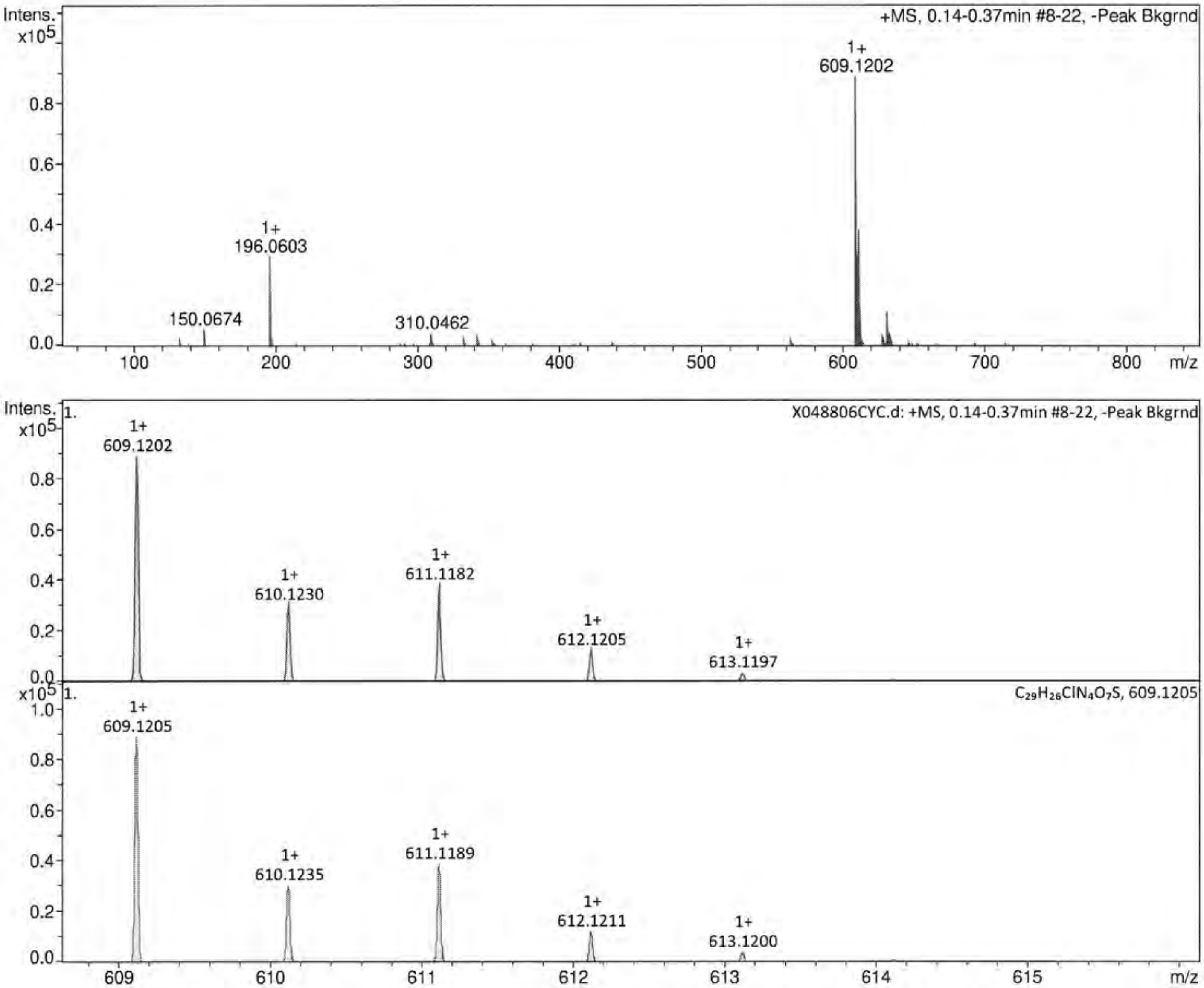

Meas. m/z	z	#	Ion Formula	m/z	err [ppm]	mSigma	rdb	e⁻ Conf
150.067376	1+	1	C9H10O2	150.067531	1.0	53.9	5.5	odd
196.060261	1+	1	C9H10NO4	196.060434	0.9	1.0	6.0	even
333.042946	2+	1	C29H27CaClN4O8S	333.042653	-0.9	31.1	19.0	even
	2+	2	C28H31CaClO12S	333.041984	-2.9	36.5	14.0	even
342.048216	2+	1	C29H29CaClN4O9S	342.047936	-0.8	22.4	18.0	even
	2+	2	C28H33CaClO13S	342.047267	-2.8	23.8	13.0	even
609.120169	1+	1	C29H26ClN4O7S	609.120524	0.6	2.8	19.0	even
	1+	2	C28H30ClO11S	609.119187	-1.6	10.6	14.0	even

Spectrométrie de Masse

Demande d'analyse HRMS

High-Resolution Mass Spectrometry analysis request form

Demandeur (Nom+Prénom) : Sava Alexandru (*Submitter's name*) : AS	Tél : (*Phone*)	Mail : (*E-mail*)
Nom de l'échantillon : AS 397 (*Sample name*) **8b**	Confirmation : ☑ Structure connue (Known structure)	Identification : ☐ Structure inconnue (Unknown structure)

Formule brute : $C_{29}H_{24}ClFN_4O_7S$
(*Molecular formula*)

Masse moléculaire : 627.04
(*Molecular weight*)

Solvant conseillé : Acetone
(*Recommended solvent*)

Concentration ou masse :
(*Concentration or weight*)

1 mg / 1mL

Structure :
(*Chemical structure*)

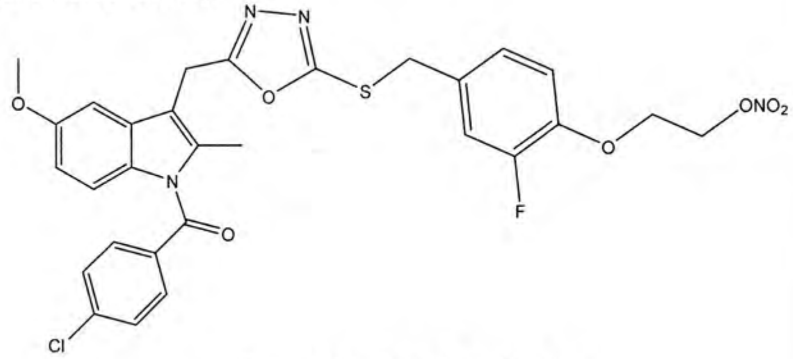

Chemical Formula: $C_{29}H_{24}ClFN_4O_7S$
Molecular Weight: 627.04

Remarques (conservation, solvants déconseillés, toxicité…) :
(*Notes: storage, sample handling precautions, toxicity …*)

Responsable : Sylvain Routier (*Supervisor*)	Date : 10.12.2019 (*Date*)	Bon pour accord : (*Signed as agreed*)

Partie réservée au service
(For MS facility use only)

Position sur le rack : (*Position on the rack*) GE12	Nom du fichier : (*File name*) X053979CYC	Date : (*Date*) 10/12/2019

Remarques :
(*Comments and suggestions*)

Plate-forme de Spectrométrie de Masse Haute Résolution – Fédération de Recherche Physique et Chimie du Vivant (FR2708 : CBM/ICOA)

Cyril Colas – Plate-forme HRMS
Institut de Chimie Organique et Analytique
Université d'Orléans – UFR Sciences
Rue de Chartres – BP 6759 – F-45067 ORLEANS Cedex 2
cyril.colas@univ-orleans.fr – +33(0)2 38 49 46 61

Fédération de Recherche Physique et Chimie du Vivant (FR2708 : CBM/ICOA)
Plate-forme de Spectrométrie de Masse Haute Résolution

HRAM

Analysis Info

Sample Name	**AS397**	Acquisition Date	11/12/2019 20:04:33
Analysis Name	X053979CYC.d	Instrument / Ser#	maXis 255552.00086
		Method	Positif.m

Acquisition Parameter

Source Type	ESI	Ion Polarity	Positive	Set Nebulizer	0.6 Bar
Scan Begin	50 m/z	Set Capillary	4500 V	Set Dry Heater	200 °C
Scan End	2500 m/z	Set Collision Cell RF	1800.0 Vpp	Set Dry Gas	7.0 l/min

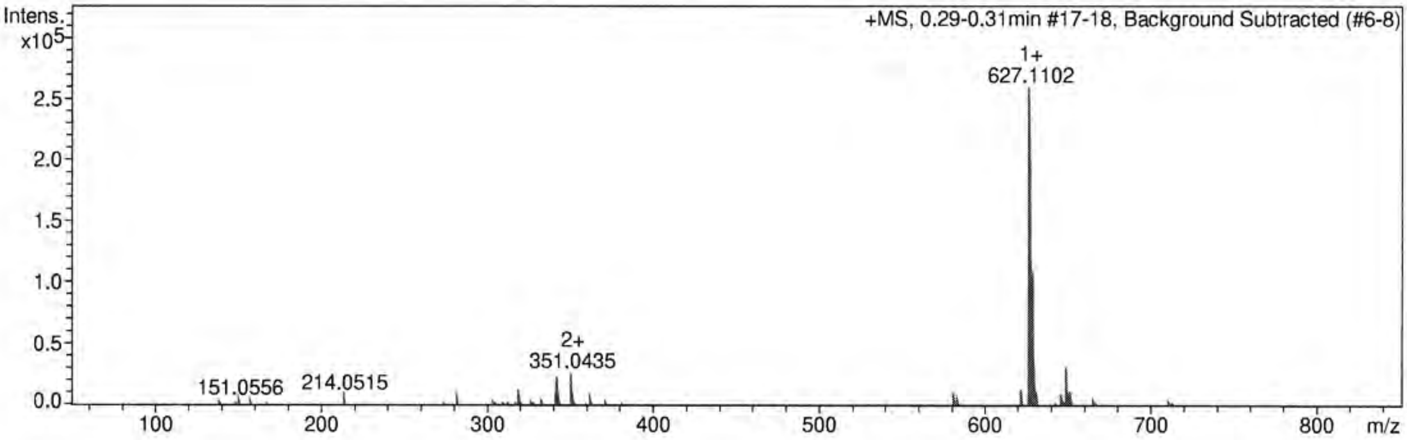

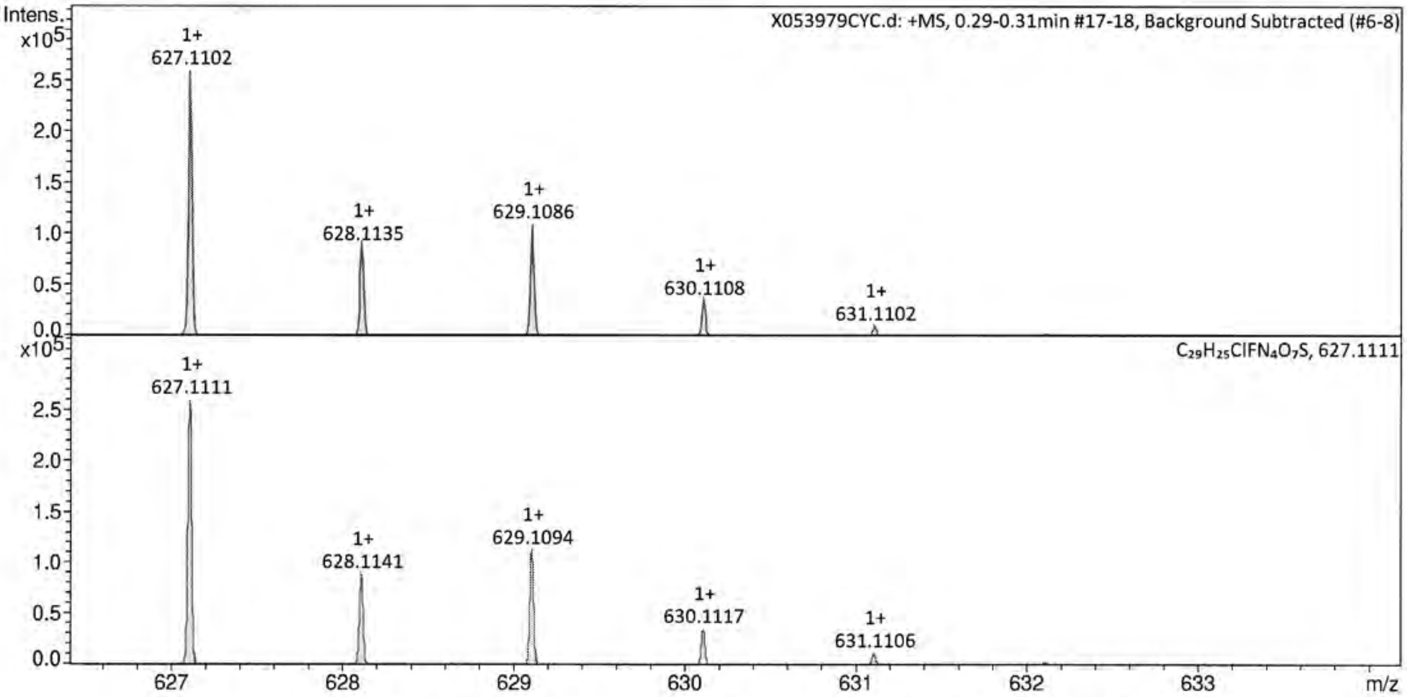

Meas. m/z	z	#	Ion Formula	m/z	err [ppm]	mSigma	rdb	e⁻ Conf
342.038352	2+	1	C29H26CaClFN4O8S	342.037942	-1.2	6.7	19.0	even
	2+	2	C17H30CaClFN6O15S	342.038868	1.5	47.0	6.0	even
351.043510	2+	1	C29H28CaClFN4O9S	351.043225	-0.8	20.6	18.0	even
581.117555	1+	1	C29H25ClFN3O5S	581.118199	1.1	12.3	18.5	odd
627.110186	1+	1	C29H25ClFN4O7S	627.111102	1.5	7.6	19.0	even
	1+	2	C28H29ClFO11S	627.109765	-0.7	11.1	14.0	even
649.092195	1+	1	C29H24ClFN4NaO7S	649.093047	1.3	21.3	19.0	even
	1+	2	C28H28ClFNaO11S	649.091709	-0.7	25.3	14.0	even

Institut de Chimie Organique et Analytique
UMR 7311 - Université d'Orléans - CNRS
BP 6759 - rue de Chartres - F-45067 ORLEANS Cedex 2
cyril.colas@univ-orleans.fr
+33 (0)2 38 49 46 61
printed: 12/12/2019 19:45:03

Meas. m/z	z	#	Ion Formula	m/z	err [ppm]	mSigma	rdb	e⁻ Conf
631.101895	1+	1	C29H25ClN4NaO7S	631.102469	0.9	21.7	19.0	even
	1+	2	C28H29ClNaO11S	631.101131	-1.2	21.9	14.0	even

Spectrométrie de Masse

Demande d'analyse HRMS

High-Resolution Mass Spectrometry analysis request form

Demandeur (Nom+Prénom) : Sava Alexandru (*Submitter's name*) : AS	Tél : (*Phone*)	Mail : (*E-mail*)
Nom de l'échantillon : AS 391 (*Sample name*) **8c**	Confirmation : ☑ Structure connue (Known structure)	Identification : ☐ Structure inconnue (Unknown structure)

Formule brute : $C_{29}H_{24}Cl_2N_4O_7S$ (*Molecular formula*)	Structure : (*Chemical structure*)
Masse moléculaire : 643.49 (*Molecular weight*)	
Solvant conseillé : Acetone (*Recommended solvent*)	
Concentration ou masse : (*Concentration or weight*) 1 mg / 1mL	

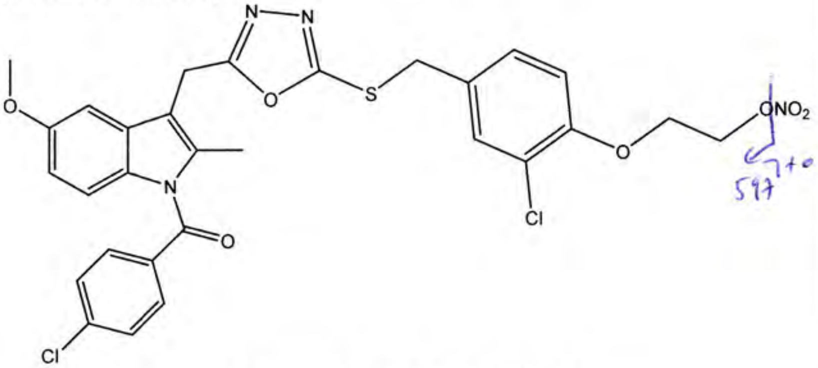

Chemical Formula: $C_{29}H_{24}Cl_2N_4O_7S$
Molecular Weight: 643.49

Remarques (conservation, solvants déconseillés, toxicité…) :
(*Notes: storage, sample handling precautions, toxicity …*)

Responsable : Sylvain Routier (*Supervisor*)	Date : 10.12.2019 (*Date*)	Bon pour accord : (*Signed as agreed*)

Partie réservée au service
(For MS facility use only)

Position sur le rack : (*Position on the rack*) GC11	Nom du fichier : (*File name*) X053954CYC	Date : (*Date*) 10/12/2019

Remarques :
(*Comments and suggestions*)

Cyril Colas – Plate-forme HRMS
Institut de Chimie Organique et Analytique
Université d'Orléans – UFR Sciences
Rue de Chartres – BP 6759 – F-45067 ORLEANS Cedex 2
cyril.colas@univ-orleans.fr – +33(0)2 38 49 46 61

Plate-forme de Spectrométrie de Masse Haute Résolution – Fédération de Recherche Physique et Chimie du Vivant (FR2708 : CBM/ICOA)

Fédération de Recherche Physique et Chimie du Vivant (FR2708 : CBM/ICOA)
Plate-forme de Spectrométrie de Masse Haute Résolution

HRAM

**Analysis Info**		Acquisition Date	11/12/2019 18:03:15
Sample Name	**AS391**	Instrument / Ser#	maXis 255552.00086
Analysis Name	X053954CYC.d	Method	Positif.m

Acquisition Parameter

Source Type	ESI	Ion Polarity	Positive	Set Nebulizer	0.6 Bar
Scan Begin	50 m/z	Set Capillary	4500 V	Set Dry Heater	200 °C
Scan End	2500 m/z	Set Collision Cell RF	1800.0 Vpp	Set Dry Gas	7.0 l/min

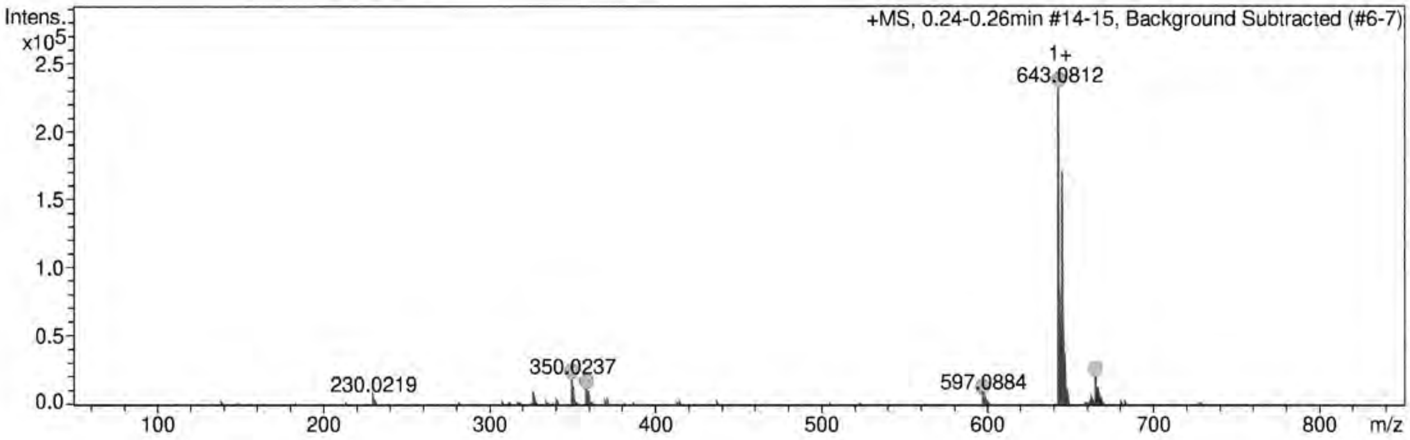

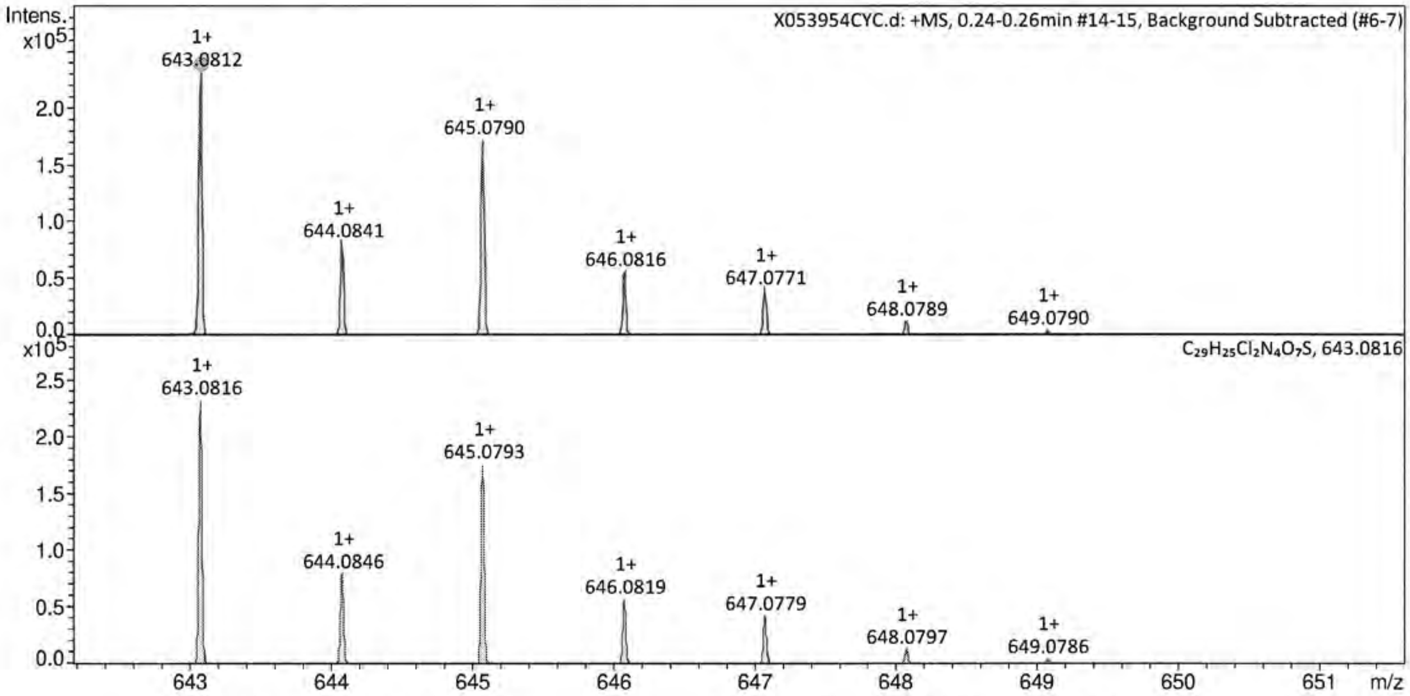

Meas. m/z	z	#	Ion Formula	m/z	err [ppm]	mSigma	rdb	e⁻ Conf
350.023664	2+	1	C29H26CaCl2N4O8S	350.023167	-1.4	7.6	19.0	even
359.029029	2+	1	C29H28CaCl2N4O9S	359.028449	-1.6	48.9	18.0	even
597.088398	1+	1	C29H25Cl2N3O5S	597.088649	0.4	50.8	18.5	odd
643.081171	1+	1	C29H25Cl2N4O7S	643.081552	0.6	8.3	19.0	even
	1+	2	C28H29Cl2O11S	643.080215	-1.5	14.1	14.0	even
665.062716	1+	1	C28H28Cl2NaO11S	665.062159	-0.8	33.1	14.0	even
	1+	2	C29H24Cl2N4NaO7S	665.063496	1.2	33.8	19.0	even

Institut de Chimie Organique et Analytique
UMR 7311 - Université d'Orléans - CNRS
BP 6759 - rue de Chartres - F-45067 ORLEANS Cedex 2
cyril.colas@univ-orleans.fr
+33 (0)2 38 49 46 61

printed: 12/12/2019 18:31:49

Spectrométrie de Masse

Demande d'analyse HRMS

High-Resolution Mass Spectrometry analysis request form

Demandeur (Nom+Prénom) : Sava Alexandru<br>(*Submitter's name*) : AS	Tél :<br>(*Phone*)	Mail :<br>(*E-mail*)
Nom de l'échantillon : AS 390<br>(*Sample name*) **8e**	Confirmation :<br>☑ Structure connue<br>(Known structure)	Identification :<br>☐ Structure inconnue<br>(Unknown structure)

Formule brute : $C_{29}H_{24}ClN_5O_9S$
(*Molecular formula*)

Masse moléculaire : 654.05
(*Molecular weight*)

Solvant conseillé : Acetone
(*Recommended solvent*)

Concentration ou masse :
(*Concentration or weight*)
1 mg / 1mL

Structure :
(*Chemical structure*)

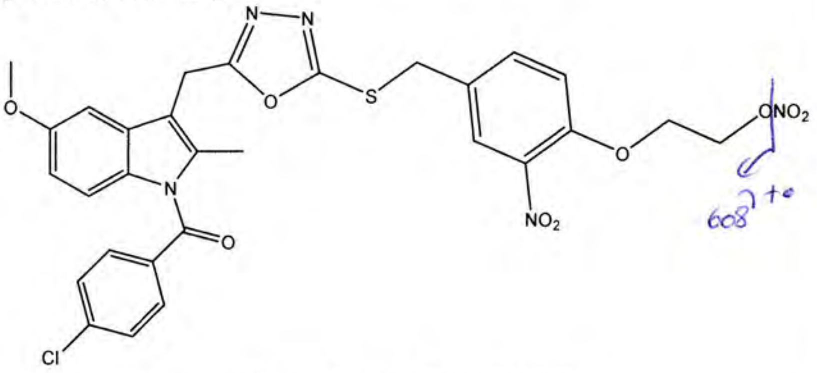

Chemical Formula: $C_{29}H_{24}ClN_5O_9S$
Molecular Weight: 654.05

Remarques (conservation, solvants déconseillés, toxicité…) :
(*Notes: storage, sample handling precautions, toxicity …*)

Responsable : Sylvain Routier<br>(*Supervisor*)	Date : 10.12.2019<br>(*Date*)	Bon pour accord :<br>(*Signed as agreed*)

Partie réservée au service
(For MS facility use only)

Position sur le rack :<br>(*Position on the rack*) GC10	Nom du fichier :<br>(*File name*) X053953CYC	Date :<br>(*Date*) 10/12/2019

Remarques :
(*Comments and suggestions*) m/z 346^{2+}, 355^{2+} = $[M+Ca]^{2+}$, $[M+Ca+H_2O]^{2+}$

Cyril Colas – Plate-forme HRMS
Institut de Chimie Organique et Analytique
Université d'Orléans – UFR Sciences
Rue de Chartres – BP 6759 – F-45067 ORLEANS Cedex 2
cyril.colas@univ-orleans.fr – +33(0)2 38 49 46 61

Plate-forme de Spectrométrie de Masse Haute Résolution – Fédération de Recherche Physique et Chimie du Vivant (FR2708 : CBM/ICOA)

Fédération de Recherche Physique et Chimie du Vivant (FR2708 : CBM/ICOA)
Plate-forme de Spectrométrie de Masse Haute Résolution

HRAM

Analysis Info

Sample Name	**AS390**	Acquisition Date	11/12/2019 18:01:40
Analysis Name	X053953CYC.d	Instrument / Ser#	maXis 255552.00086
		Method	Positif.m

Acquisition Parameter

Source Type	ESI	Ion Polarity	Positive	Set Nebulizer	0.6 Bar
Scan Begin	50 m/z	Set Capillary	4500 V	Set Dry Heater	200 °C
Scan End	2500 m/z	Set Collision Cell RF	1800.0 Vpp	Set Dry Gas	7.0 l/min

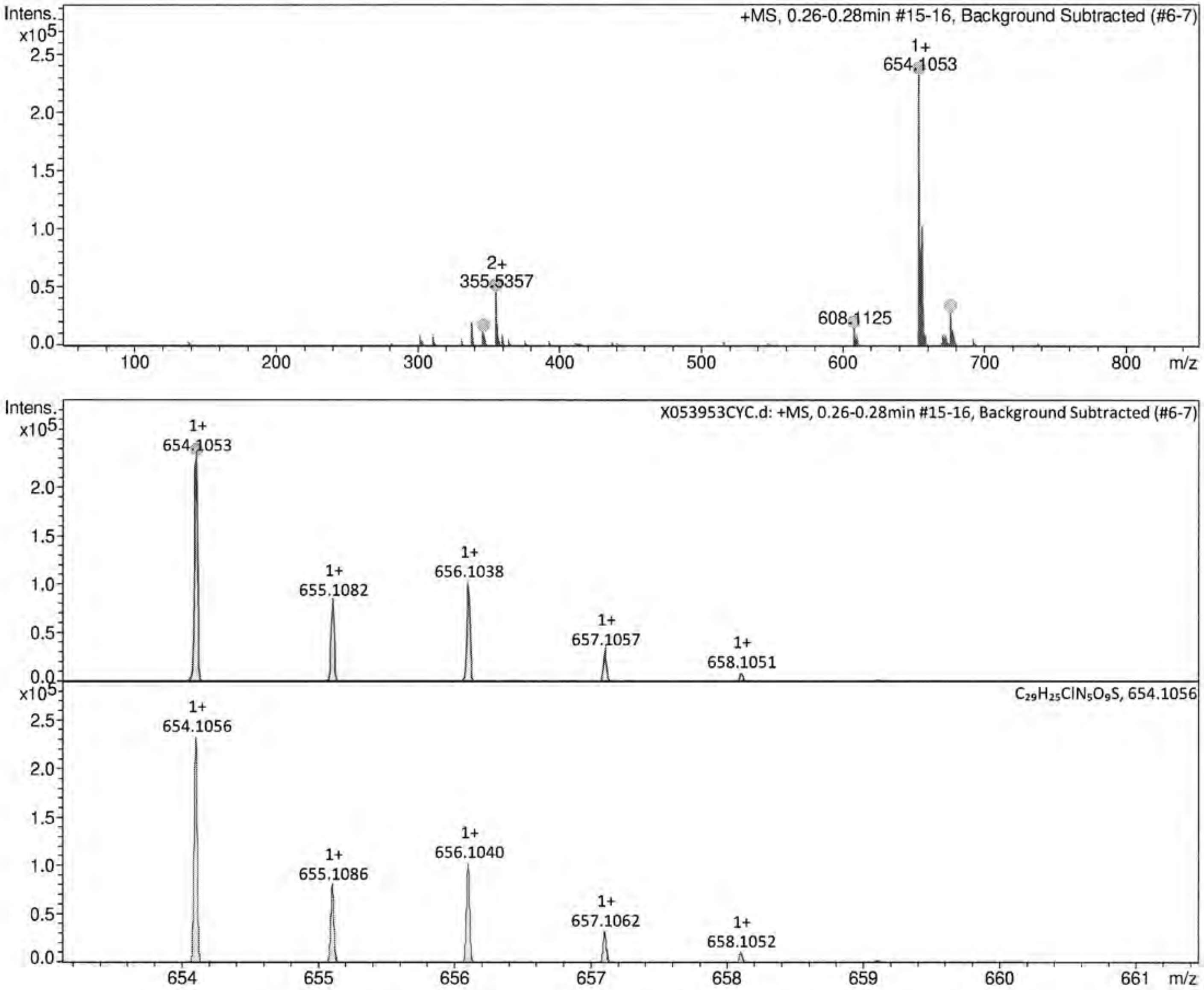

Meas. m/z	z	#	Ion Formula	m/z	err [ppm]	mSigma	rdb	e⁻ Conf
346.530830	2+	1	C29H24CaClN5O9S	346.529910	-2.7	31.7	21.0	even
355.535663	2+	1	C29H26CaClN5O10S	355.535192	-1.3	13.8	20.0	even
608.112510	1+	1	C29H25ClN4O7S	608.112699	0.3	13.9	19.5	odd
	1+	2	C28H29ClO11S	608.111362	-1.9	21.2	14.5	odd
654.105294	1+	1	C29H25ClN5O9S	654.105603	0.5	4.9	20.0	even
	1+	2	C28H29ClNO13S	654.104265	-1.6	14.3	15.0	even
676.087160	1+	1	C29H24ClN5NaO9S	676.087547	0.6	13.8	20.0	even
	1+	2	C28H28ClNNaO13S	676.086209	-1.4	18.1	15.0	even

Institut de Chimie Organique et Analytique
UMR 7311 - Université d'Orléans - CNRS
BP 6759 - rue de Chartres - F-45067 ORLEANS Cedex 2
cyril.colas@univ-orleans.fr
+33 (0)2 38 49 46 61

Spectrométrie de Masse

Demande d'analyse HRMS

High-Resolution Mass Spectrometry analysis request form

Demandeur (Nom+Prénom) : Sava Alexandru<br>(*Submitter's name*) : AS	Tél :<br>(*Phone*)	Mail :<br>(*E-mail*)

Nom de l'échantillon : AS 331<br>(*Sample name*) **8f**	Confirmation :<br>☑ Structure connue<br>(Known structure)	Identification :<br>☐ Structure inconnue<br>(Unknown structure)

Formule brute : $C_{30}H_{27}ClN_4O_8S$
(*Molecular formula*)

Masse moléculaire : 639,08
(*Molecular weight*)

Solvant conseillé : Acetone
(*Recommended solvent*)

Concentration ou masse :
(*Concentration or weight*)
1 mg / 1mL

Structure :
(*Chemical structure*)

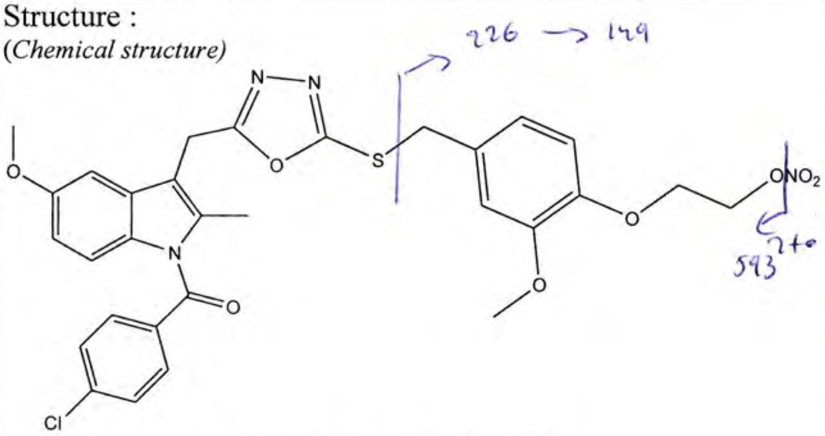

Chemical Formula: $C_{30}H_{27}ClN_4O_8S$
Molecular Weight: 639.08

Remarques (conservation, solvants déconseillés, toxicité…) :
(*Notes: storage, sample handling precautions, toxicity …*)

Responsable : Sylvain Routier<br>(*Supervisor*)	Date : 06.03.2018<br>(*Date*)	Bon pour accord :<br>(*Signed as agreed*)

Partie réservée au service
(For MS facility use only)

Position sur le rack :<br>(*Position on the rack*) GE11	Nom du fichier :<br>(*File name*) X048807CYC	Date :<br>(*Date*) 13/03/2019

Remarques :
(*Comments and suggestions*) m/z 339^{2+}, 348^{2+} = $[M+Ca]^{2+}$, $[M+Ca+H_2O]^{2+}$

Plate-forme de Spectrométrie de Masse Haute Résolution – Fédération de Recherche Physique et Chimie du Vivant (FR2708 : CBM/ICOA)

Cyril Colas – Plate-forme HRMS
Institut de Chimie Organique et Analytique
Université d'Orléans – UFR Sciences
Rue de Chartres – BP 6759 – F-45067 ORLEANS Cedex 2
cyril.colas@univ-orleans.fr – +33(0)2 38 49 46 61

Analysis Info

Sample Name	**AS331**	Acquisition Date	13/03/2019 19:48:23
Analysis Name	X048807CYC.d	Instrument / Ser#	maXis 255552.00086
		Method	positif-6.m

Acquisition Parameter

Source Type	ESI	Ion Polarity	Positive	Set Nebulizer	0.6 Bar
Scan Begin	50 m/z	Set Capillary	4500 V	Set Dry Heater	200 °C
Scan End	2500 m/z	Set Collision Cell RF	1800.0 Vpp	Set Dry Gas	7.0 l/min

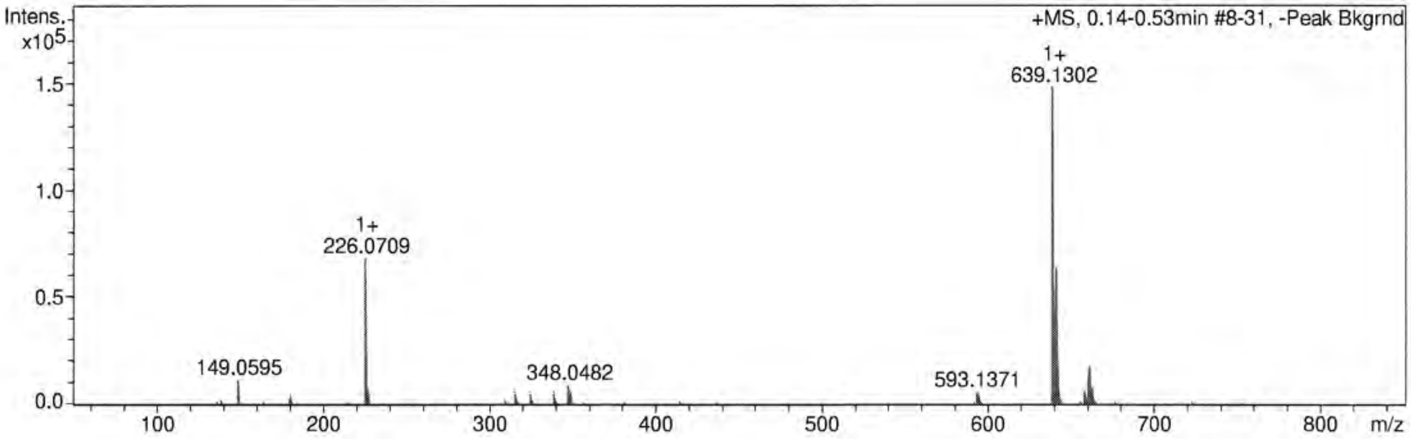

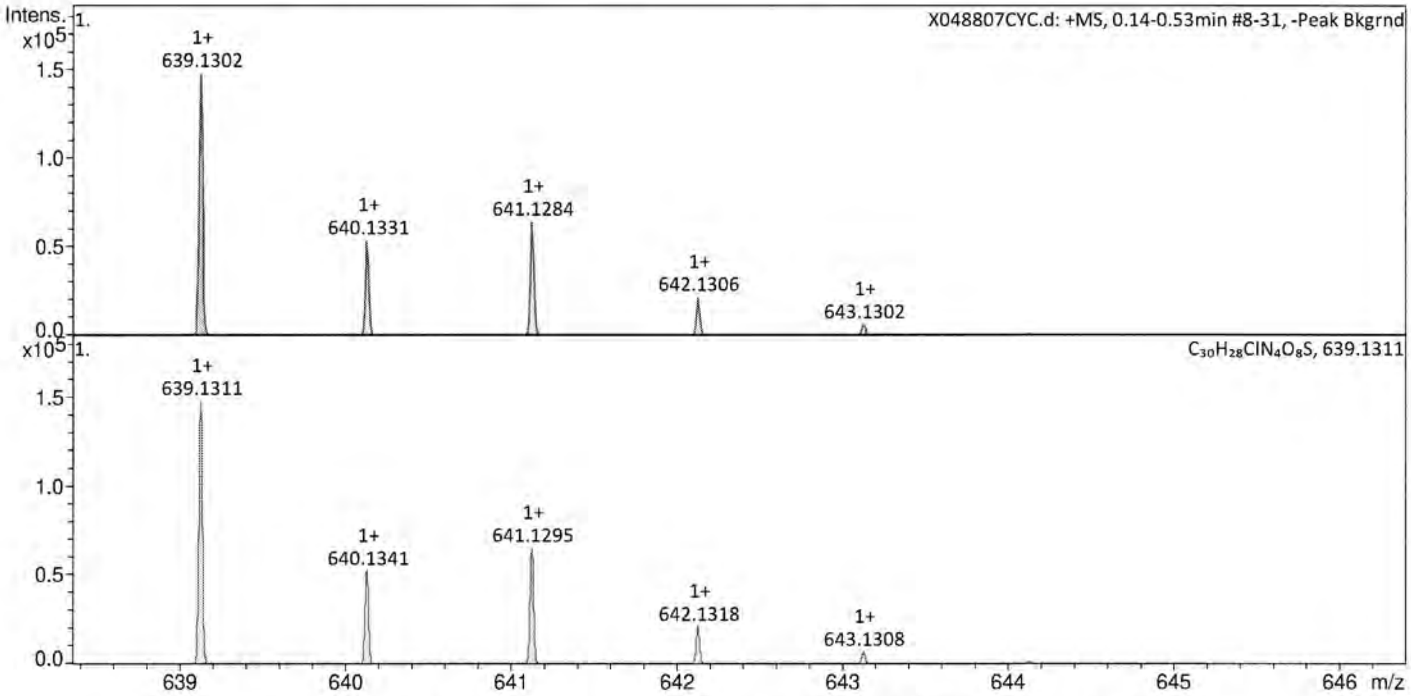

Meas. m/z	z	#	Ion Formula	m/z	err [ppm]	mSigma	rdb	e⁻ Conf
149.059480	1+	1	C9H9O2	149.059706	1.5	5.3	6.0	even
226.070885	1+	1	C10H12NO5	226.070999	0.5	4.6	6.0	even
339.042764	2+	1	C30H27CaClN4O8S	339.042653	-0.3	17.1	20.0	even
348.048173	2+	1	C30H29CaClN4O9S	348.047936	-0.7	16.9	19.0	even
593.137109	1+	1	C30H28ClN3O6S	593.138186	1.8	23.5	18.5	odd
639.130233	1+	1	C30H28ClN4O8S	639.131089	1.3	6.0	19.0	even
	1+	2	C29H32ClO12S	639.129752	-0.8	15.1	14.0	even
661.111962	1+	1	C30H27ClN4NaO8S	661.113033	1.6	5.8	19.0	even

Meas. m/z	z	#	Ion Formula	m/z	err [ppm]	mSigma	rdb	e⁻ Conf
	1+	2	C29H31ClNaO12S	661.111696	-0.4	8.5	14.0	even

Spectrométrie de Masse

Demande d'analyse HRMS

High-Resolution Mass Spectrometry analysis request form

Demandeur (Nom+Prénom) : Sava Alexandru<br>(*Submitter's name*) : AS	Tél :<br>(*Phone*)	Mail :<br>(*E-mail*)
Nom de l'échantillon : AS 392<br>(*Sample name*) **8k**	Confirmation :<br>☑ Structure connue<br>(Known structure)	Identification :<br>☐ Structure inconnue<br>(Unknown structure)

Formule brute : $C_{29}H_{24}ClN_5O_9S$<br>(*Molecular formula*)	Structure :<br>(*Chemical structure*)
Masse moléculaire : 654.05<br>(*Molecular weight*)	
Solvant conseillé : Acetone<br>(*Recommended solvent*)	
Concentration ou masse :<br>(*Concentration or weight*)<br>1 mg / 1mL	

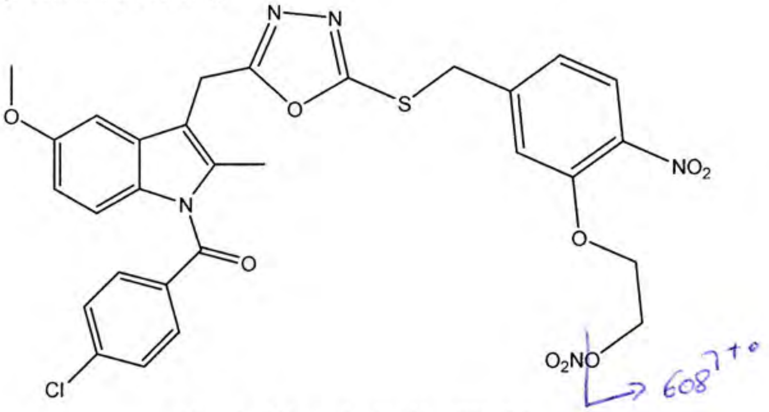

Chemical Formula: $C_{29}H_{24}ClN_5O_9S$
Molecular Weight: 654.05

Remarques (conservation, solvants déconseillés, toxicité…) :
(*Notes: storage, sample handling precautions, toxicity …*)

Responsable : Sylvain Routier<br>(*Supervisor*)	Date : 10.12.2019<br>(*Date*)	Bon pour accord :<br>(*Signed as agreed*)

Partie réservée au service
(*For MS facility use only*)

Position sur le rack :<br>(*Position on the rack*) GC12	Nom du fichier :<br>(*File name*) X053955CYC	Date :<br>(*Date*) 10/12/2019

Remarques :
(*Comments and suggestions*)

Cyril Colas – Plate-forme HRMS
Institut de Chimie Organique et Analytique
Université d'Orléans – UFR Sciences
Rue de Chartres – BP 6759 – F-45067 ORLEANS Cedex 2
cyril.colas@univ-orleans.fr – +33(0)2 38 49 46 61

Plate-forme de Spectrométrie de Masse Haute Résolution – Fédération de Recherche Physique et Chimie du Vivant (FR2708 : CBM/ICOA)

Analysis Info

Sample Name	**AS392**	Acquisition Date	11/12/2019 18:04:51
Analysis Name	X053955CYC.d	Instrument / Ser#	maXis 255552.00086
		Method	Positif.m

Acquisition Parameter

Source Type	ESI	Ion Polarity	Positive	Set Nebulizer	0.6 Bar
Scan Begin	50 m/z	Set Capillary	4500 V	Set Dry Heater	200 °C
Scan End	2500 m/z	Set Collision Cell RF	1800.0 Vpp	Set Dry Gas	7.0 l/min

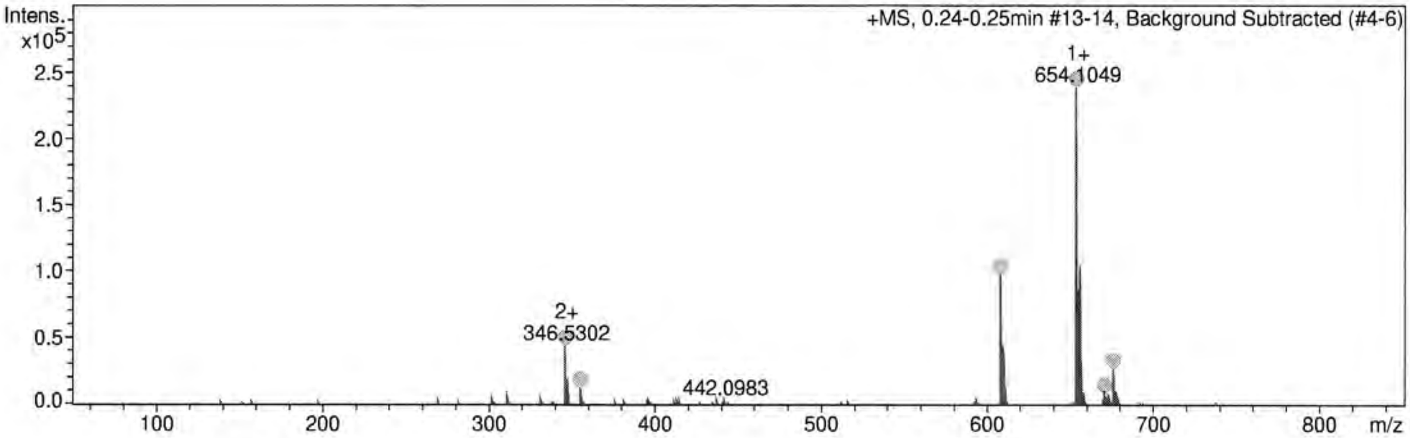

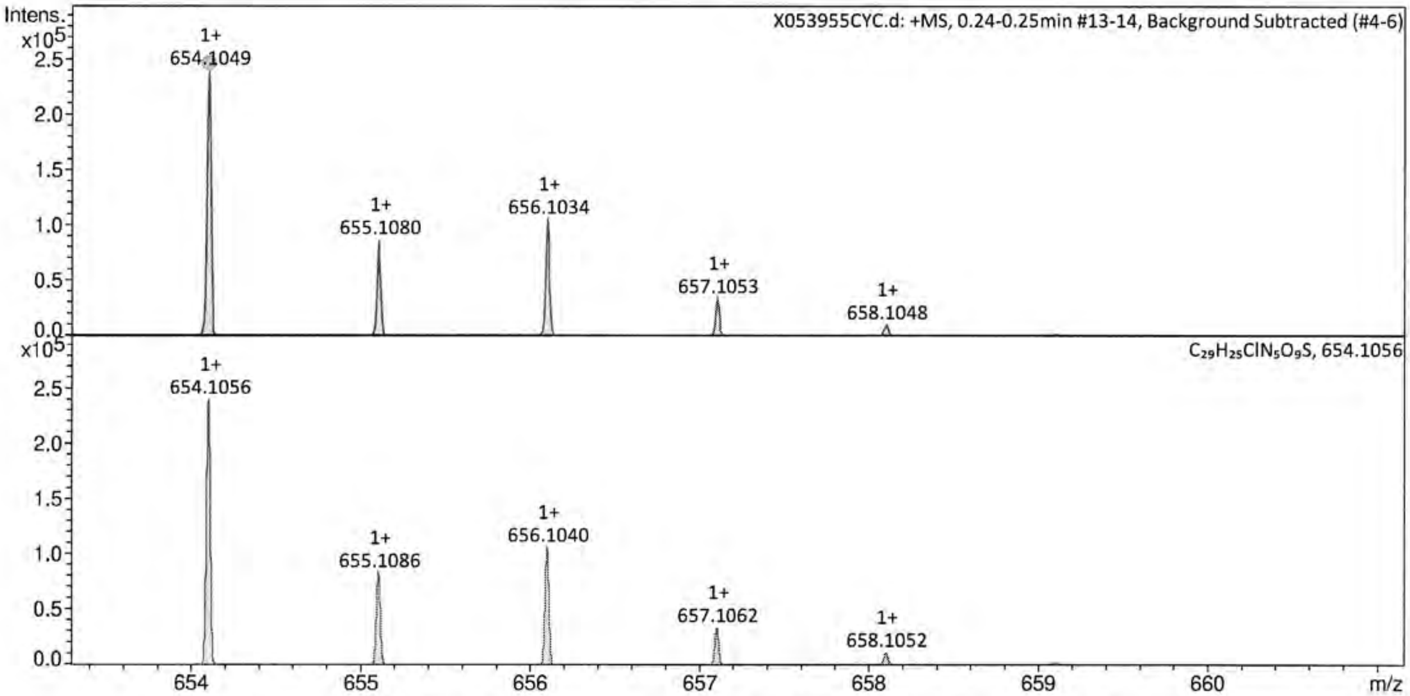

Meas. m/z	z	#	Ion Formula	m/z	err [ppm]	mSigma	rdb	e⁻ Conf
346.530226	2+	1	C29H24CaClN5O9S	346.529910	-0.9	10.3	21.0	even
355.535162	2+	1	C29H26CaClN5O10S	355.535192	0.1	13.9	20.0	even
	2+	2	C28H30CaClNO14S	355.534524	-1.8	18.6	15.0	even
608.112509	1+	1	C29H25ClN4O7S	608.112699	0.3	6.2	19.5	odd
	1+	2	C28H29ClO11S	608.111362	-1.9	16.1	14.5	odd
654.104904	1+	1	C29H25ClN5O9S	654.105603	1.1	6.9	20.0	even
	1+	2	C28H29ClNO13S	654.104265	-1.0	16.7	15.0	even
671.131445	1+	1	C29H28ClN6O9S	671.132152	1.1	34.8	19.0	even

Institut de Chimie Organique et Analytique
UMR 7311 - Université d'Orléans - CNRS
BP 6759 - rue de Chartres - F-45067 ORLEANS Cedex 2
cyril.colas@univ-orleans.fr
+33 (0)2 38 49 46 61

Meas. m/z	z	#	Ion Formula	m/z	err [ppm]	mSigma	rdb	e⁻ Conf
	1+	2	C28H32ClN2O13S	671.130814	-0.9	35.4	14.0	even
676.086834	1+	1	C29H24ClN5NaO9S	676.087547	1.1	9.9	20.0	even
	1+	2	C28H28ClNNaO13S	676.086209	-0.9	14.4	15.0	even

Spectrométrie de Masse

Demande d'analyse HRMS

High-Resolution Mass Spectrometry analysis request form

Demandeur (Nom+Prénom) : Sava Alexandru<br>(*Submitter's name*) : AS	Tél :<br>(*Phone*)	Mail :<br>(*E-mail*)

Nom de l'échantillon : AS 394<br>(*Sample name*)<br>**8l**	Confirmation :<br>☑ Structure connue<br>(Known structure)<br>Identification :<br>☐ Structure inconnue<br>(Unknown structure)
Formule brute : $C_{30}H_{26}Cl_2N_4O_8S$<br>(*Molecular formula*)	Structure :<br>(*Chemical structure*)
Masse moléculaire : 673.52<br>(*Molecular weight*)	
Solvant conseillé : Acetone<br>(*Recommended solvent*)	
Concentration ou masse :<br>(*Concentration or weight*)<br>1 mg / 1mL	

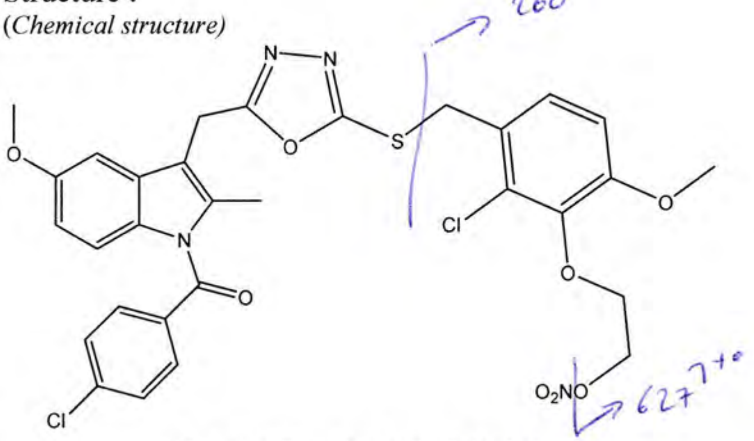

Chemical Formula: $C_{30}H_{26}Cl_2N_4O_8S$

Molecular Weight: 673.52

Remarques (conservation, solvants déconseillés, toxicité…) :
(*Notes: storage, sample handling precautions, toxicity …*)

Responsable : Sylvain Routier<br>(*Supervisor*)	Date : 10.12.2019<br>(*Date*)	Bon pour accord :<br>(*Signed as agreed*)

Partie réservée au service
(For MS facility use only)

Position sur le rack :<br>(*Position on the rack*) GD2	Nom du fichier :<br>(*File name*) X053957CYC	Date :<br>(*Date*) 10/12/2019

Remarques :
(*Comments and suggestions*)

Cyril Colas – Plate-forme HRMS
Institut de Chimie Organique et Analytique
Université d'Orléans – UFR Sciences
Rue de Chartres – BP 6759 – F-45067 ORLEANS Cedex 2
cyril.colas@univ-orleans.fr – +33(0)2 38 49 46 61

Plate-forme de Spectrométrie de Masse Haute Résolution – Fédération de Recherche Physique et Chimie du Vivant (FR2708 : CBM/ICOA)

Fédération de Recherche Physique et Chimie du Vivant (FR2708 : CBM/ICOA)
Plate-forme de Spectrométrie de Masse Haute Résolution

HRAM

Analysis Info

Sample Name	**AS394**	Acquisition Date	11/12/2019 18:07:51
Analysis Name	X053957CYC.d	Instrument / Ser#	maXis 255552.00086
		Method	Positif.m

Acquisition Parameter

Source Type	ESI	Ion Polarity	Positive	Set Nebulizer	0.6 Bar
Scan Begin	50 m/z	Set Capillary	4500 V	Set Dry Heater	200 °C
Scan End	2500 m/z	Set Collision Cell RF	1800.0 Vpp	Set Dry Gas	7.0 l/min

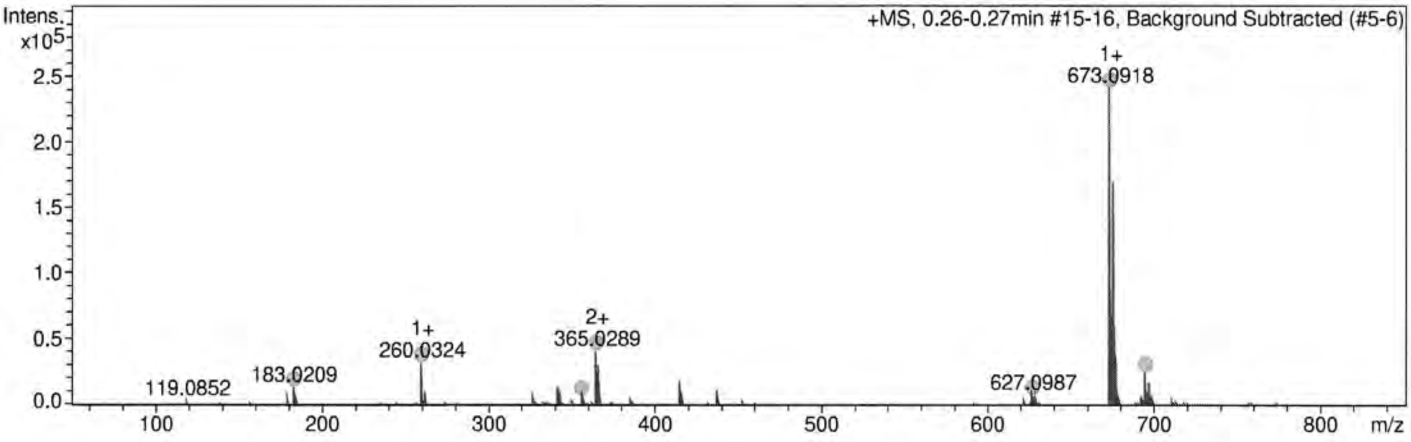

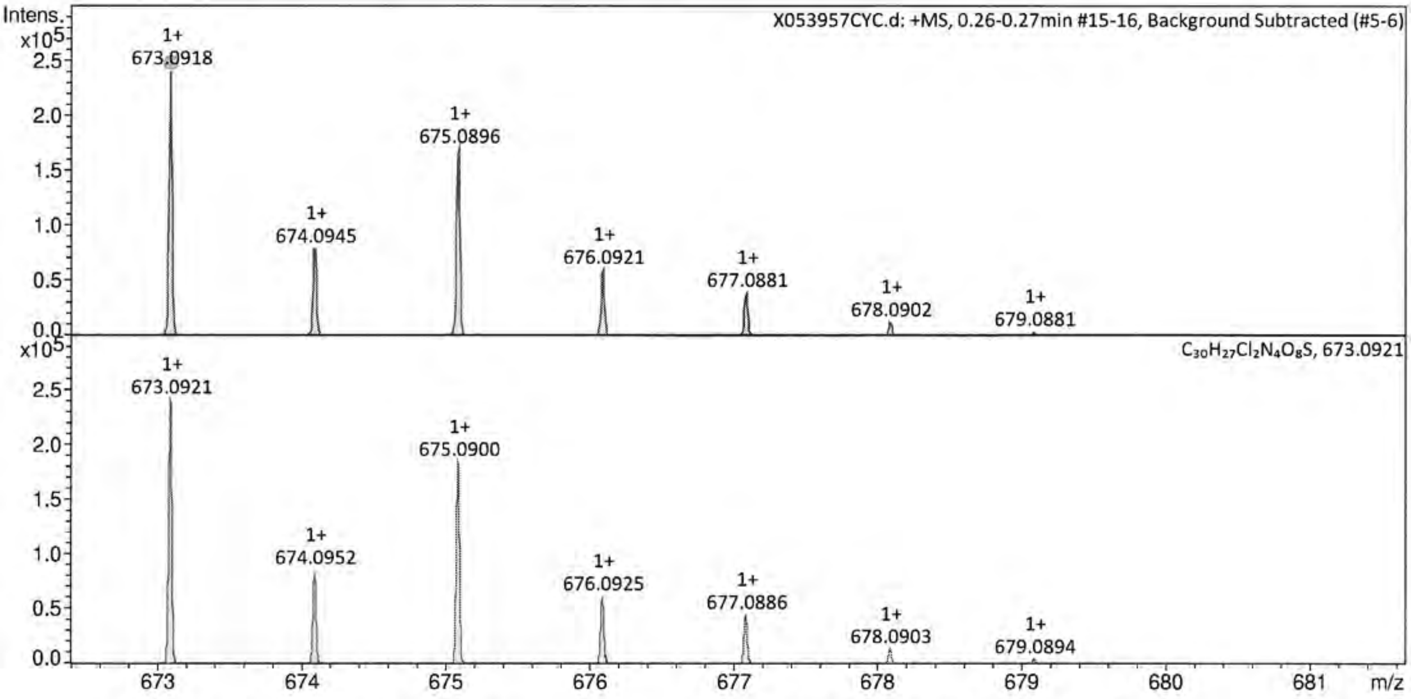

Meas. m/z	z	#	Ion Formula	m/z	err [ppm]	mSigma	rdb	e^- Conf
183.020901	1+	1	C9H8ClO2	183.020734	-0.9	6.2	6.0	even
260.032370	1+	1	C10H11ClNO5	260.032027	-1.3	24.8	6.0	even
356.024005	2+	1	C30H26CaCl2N4O8S	356.023167	-2.4	22.5	20.0	even
365.028941	2+	1	C30H28CaCl2N4O9S	365.028449	-1.3	11.3	19.0	even
627.098713	1+	1	C30H27Cl2N3O6S	627.099213	0.8	51.2	18.5	odd
673.091815	1+	1	C29H31Cl2O12S	673.090779	-1.5	24.8	14.0	even
	1+	2	C30H27Cl2N4O8S	673.092117	0.4	26.4	19.0	even
695.073430	1+	1	C30H26Cl2N4NaO8S	695.074061	0.9	23.5	19.0	even

Institut de Chimie Organique et Analytique
UMR 7311 - Université d'Orléans - CNRS
BP 6759 - rue de Chartres - F-45067 ORLEANS Cedex 2
cyril.colas@univ-orleans.fr
+33 (0)2 38 49 46 61

Meas. m/z	z	#	Ion Formula	m/z	err [ppm]	mSigma	rdb	e⁻ Conf
	1+	2	C29H30Cl2NaO12S	695.072723	-1.0	23.8	14.0	even

Institut de Chimie Organique et Analytique
UMR 7311 - Université d'Orléans - CNRS
BP 6759 - rue de Chartres - F-45067 ORLEANS Cedex 2
cyril.colas@univ-orleans.fr
+33 (0)2 38 49 46 61

Spectrométrie de Masse

Demande d'analyse HRMS

High-Resolution Mass Spectrometry analysis request form

<table>
<tr><td>Demandeur (Nom+Prénom) :
Sava Alexandru
(Submitter's name) : AS</td><td>Tél :
(Phone)</td><td>Mail :
(E-mail)</td></tr>
<tr><td>Nom de l'échantillon : AS 393
(Sample name) 8m</td><td colspan="2">Confirmation : ☑ Structure connue (Known structure)   Identification : ☐ Structure inconnue (Unknown structure)</td></tr>
<tr><td>Formule brute : $C_{30}H_{26}BrClN_4O_8S$
(Molecular formula)</td><td colspan="2" rowspan="4">Structure :
(Chemical structure)
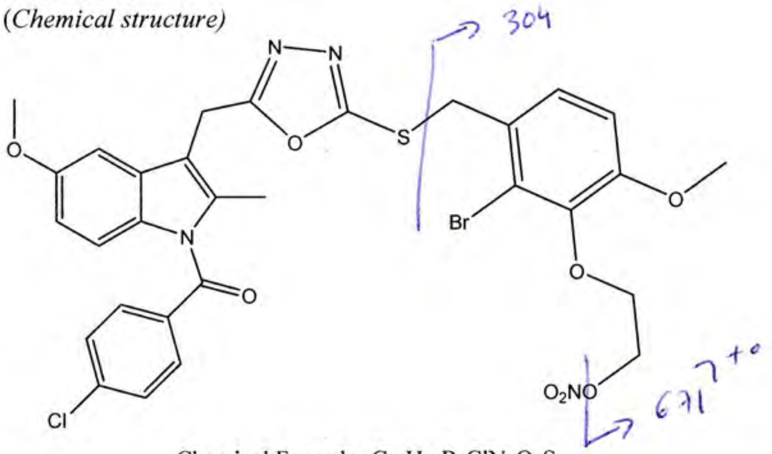
Chemical Formula: $C_{30}H_{26}BrClN_4O_8S$
Molecular Weight: 717.97</td></tr>
<tr><td>Masse moléculaire : 717.97
(Molecular weight)</td></tr>
<tr><td>Solvant conseillé : Acetone
(Recommended solvent)</td></tr>
<tr><td>Concentration ou masse :
(Concentration or weight)
1 mg / 1mL</td></tr>
<tr><td colspan="3">Remarques (conservation, solvants déconseillés, toxicité…) :
(Notes: storage, sample handling precautions, toxicity …)</td></tr>
<tr><td>Responsable : Sylvain Routier
(Supervisor)</td><td>Date : 10.12.2019
(Date)</td><td>Bon pour accord :
(Signed as agreed)</td></tr>
</table>

<table>
<tr><td colspan="3">Partie réservée au service
(For MS facility use only)</td></tr>
<tr><td>Position sur le rack :
(Position on the rack) GD1</td><td>Nom du fichier :
(File name) X053956CYC</td><td>Date :
(Date) 10/12/2019</td></tr>
<tr><td colspan="3">Remarques :
(Comments and suggestions)</td></tr>
</table>

Plate-forme de Spectrométrie de Masse Haute Résolution – Fédération de Recherche Physique et Chimie du Vivant (FR2708 : CBM/ICOA)

Cyril Colas – Plate-forme HRMS
Institut de Chimie Organique et Analytique
Université d'Orléans – UFR Sciences
Rue de Chartres – BP 6759 – F-45067 ORLEANS Cedex 2
cyril.colas@univ-orleans.fr – +33(0)2 38 49 46 61

Analysis Info

Sample Name	**AS393**	Acquisition Date	11/12/2019 18:06:20
Analysis Name	X053956CYC.d	Instrument / Ser#	maXis 255552.00086
		Method	Positif.m

Acquisition Parameter

Source Type	ESI	Ion Polarity	Positive	Set Nebulizer	0.6 Bar
Scan Begin	50 m/z	Set Capillary	4500 V	Set Dry Heater	200 °C
Scan End	2500 m/z	Set Collision Cell RF	1800.0 Vpp	Set Dry Gas	7.0 l/min

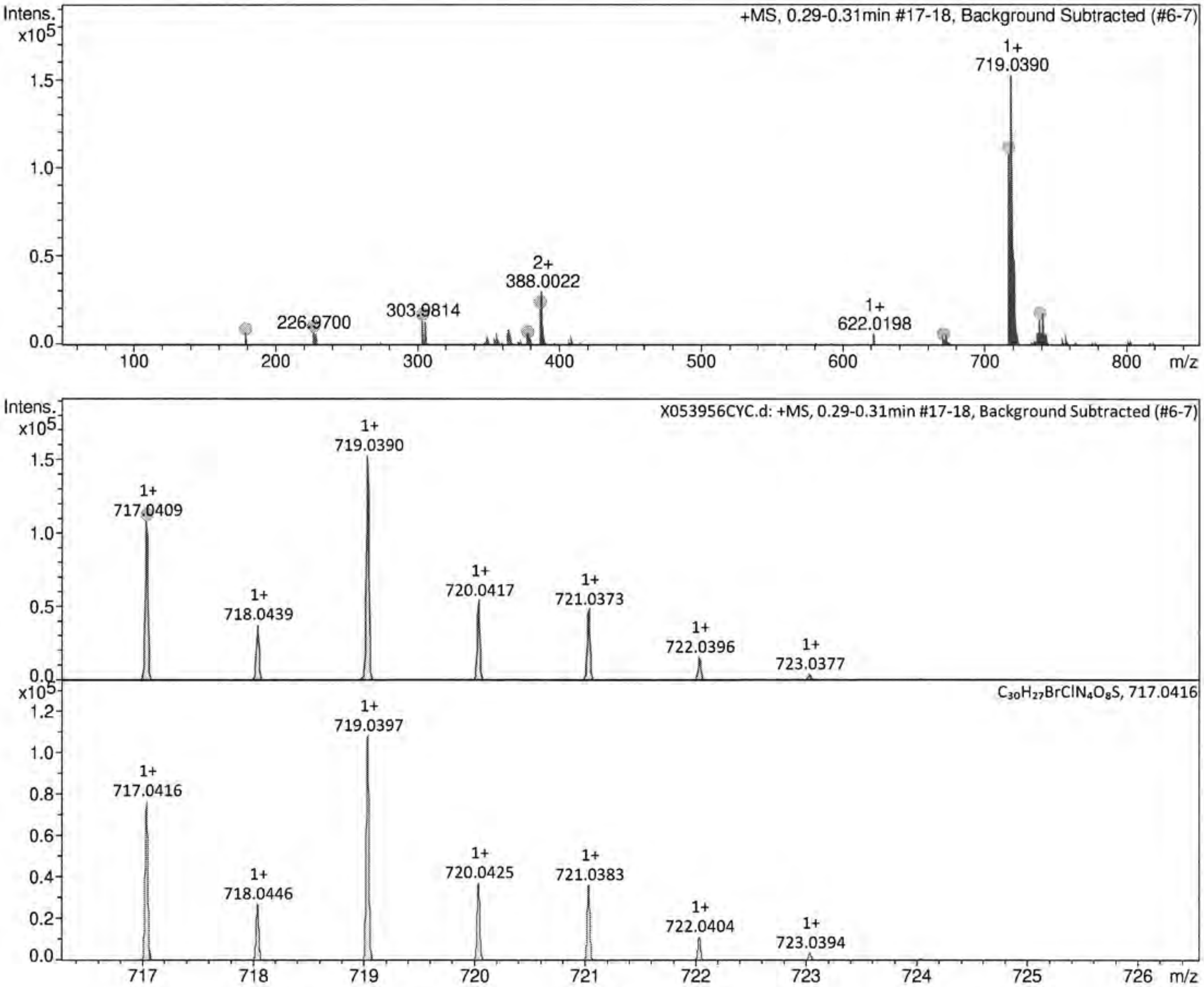

Meas. m/z	z	#	Ion Formula	m/z	err [ppm]	mSigma	rdb	e⁻ Conf
179.070251	1+	1	C10H11O3	179.070271	0.1	7.0	6.0	even
226.970029	1+	1	C9H8BrO2	226.970218	0.8	26.4	6.0	even
303.981410	1+	1	C10H11BrNO5	303.981511	0.3	18.8	6.0	even
377.997802	2+	1	C30H26BrCaClN4O8S	377.997909	0.3	53.8	20.0	even
	2+	2	C29H30BrCaClO12S	377.997240	-1.5	59.9	15.0	even
387.003215	2+	1	C30H28BrCaClN4O9S	387.003192	-0.1	16.6	19.0	even
	2+	2	C29H32BrCaClO13S	387.002523	-1.8	17.5	14.0	even
671.047665	1+	1	C30H27BrClN3O6S	671.048698	1.5	70.0	18.5	odd

Meas. m/z	z	#	Ion Formula	m/z	err [ppm]	mSigma	rdb	e⁻ Conf
717.040884	1+	1	C30H27BrClN4O8S	717.041601	1.0	8.4	19.0	even
	1+	2	C29H31BrClO12S	717.040264	-0.9	15.2	14.0	even
739.022168	1+	1	C30H26BrClN4NaO8S	739.023545	1.9	42.7	19.0	even
	1+	2	C29H30BrClNaO12S	739.022208	0.1	45.2	14.0	even

Spectrométrie de Masse

Demande d'analyse HRMS

High-Resolution Mass Spectrometry analysis request form

Demandeur (Nom+Prénom) : Sava Alexandru (*Submitter's name*) : AS	Tél : (*Phone*)	Mail : (*E-mail*)
Nom de l'échantillon : AS 395 (*Sample name*) **8n**	Confirmation : ☑ Structure connue (Known structure)	Identification : ☐ Structure inconnue (Unknown structure)

Formule brute : $C_{29}H_{25}ClN_4O_7S$
(*Molecular formula*)

Masse moléculaire : 609.05
(*Molecular weight*)

Solvant conseillé : Acetone
(*Recommended solvent*)

Concentration ou masse :
(*Concentration or weight*)
1 mg / 1mL

Structure :
(*Chemical structure)*

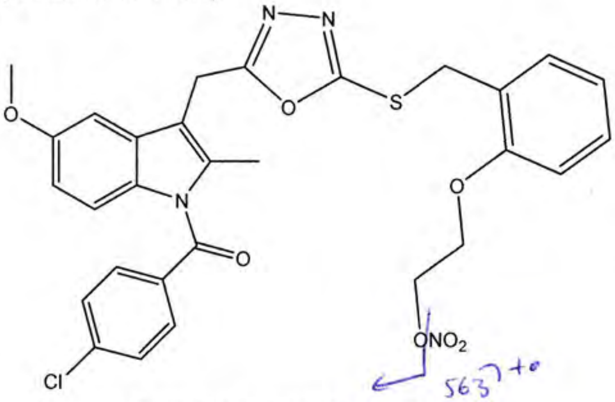

Chemical Formula: $C_{29}H_{25}ClN_4O_7S$
Molecular Weight: 609.05

Remarques (conservation, solvants déconseillés, toxicité…) :
(*Notes: storage, sample handling precautions, toxicity …*)

Responsable : Sylvain Routier (*Supervisor*)	Date : 10.12.2019 (*Date*)	Bon pour accord : (*Signed as agreed*)

Partie réservée au service
(*For MS facility use only)*

Position sur le rack : (*Position on the rack*) GE11	Nom du fichier : (*File name*) X053978CYC	Date : (*Date*) 10/12/2019

Remarques :
(*Comments and suggestions*)

Plate-forme de Spectrométrie de Masse Haute Résolution – Fédération de Recherche Physique et Chimie du Vivant (FR2708 : CBM/ICOA)

Cyril Colas – Plate-forme HRMS
Institut de Chimie Organique et Analytique
Université d'Orléans – UFR Sciences
Rue de Chartres – BP 6759 – F-45067 ORLEANS Cedex 2
cyril.colas@univ-orleans.fr – +33(0)2 38 49 46 61

Fédération de Recherche Physique et Chimie du Vivant (FR2708 : CBM/ICOA)
Plate-forme de Spectrométrie de Masse Haute Résolution

HRAM

Analysis Info

Sample Name	**AS395**	Acquisition Date	11/12/2019 20:03:05
Analysis Name	X053978CYC.d	Instrument / Ser#	maXis 255552.00086
		Method	Positif.m

Acquisition Parameter

Source Type	ESI	Ion Polarity	Positive	Set Nebulizer	0.6 Bar
Scan Begin	50 m/z	Set Capillary	4500 V	Set Dry Heater	200 °C
Scan End	2500 m/z	Set Collision Cell RF	1800.0 Vpp	Set Dry Gas	7.0 l/min

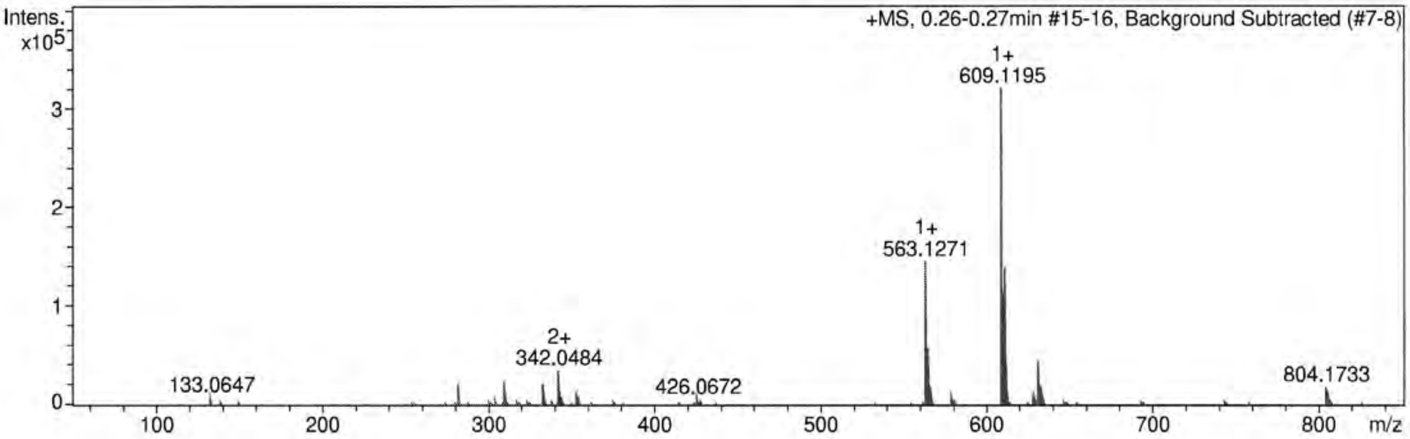

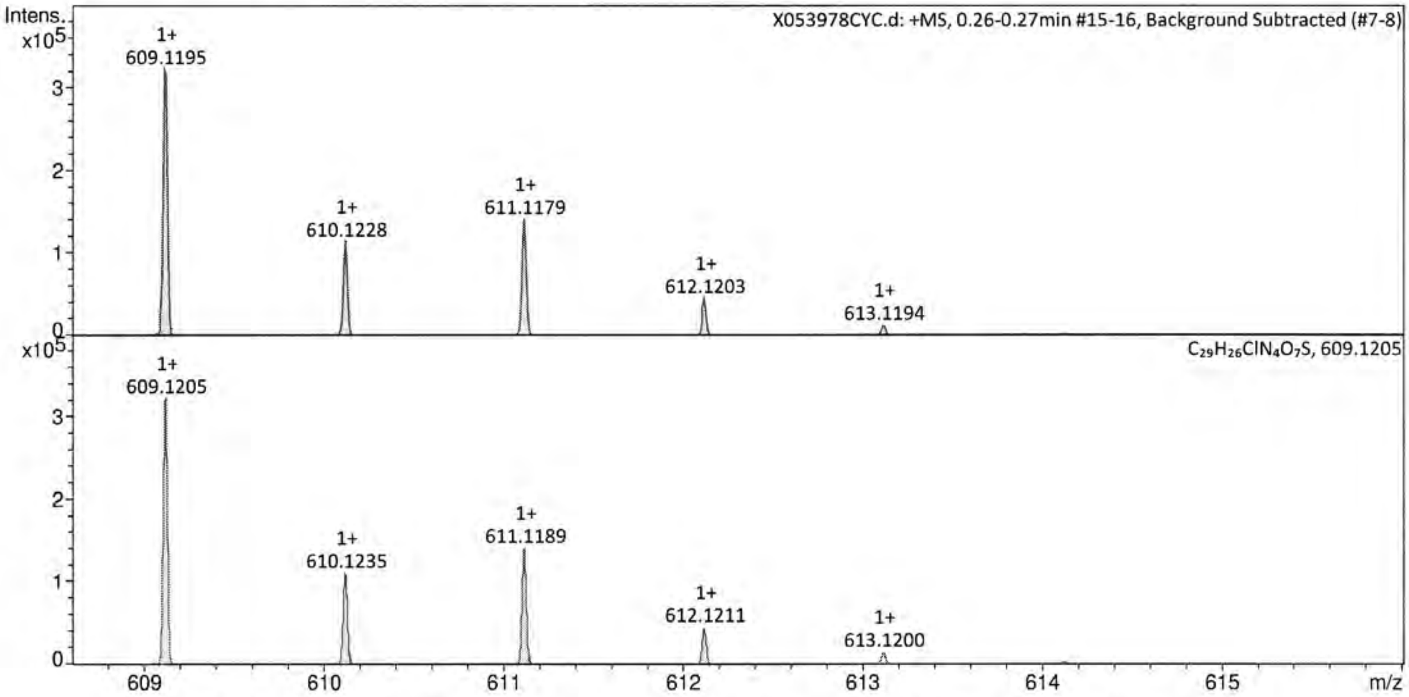

Meas. m/z	z	#	Ion Formula	m/z	err [ppm]	mSigma	rdb	e⁻ Conf
333.043015	2+	1	C29H27CaClN4O8S	333.042653	-1.1	12.9	19.0	even
342.048403	2+	1	C29H29CaClN4O9S	342.047936	-1.4	47.8	18.0	even
563.127138	1+	1	C29H26ClN3O5S	563.127621	0.9	16.5	18.5	odd
609.119494	1+	1	C29H26ClN4O7S	609.120524	1.7	4.7	19.0	even
	1+	2	C28H30ClO11S	609.119187	-0.5	14.3	14.0	even
631.101636	1+	1	C29H25ClN4NaO7S	631.102469	1.3	7.6	19.0	even
	1+	2	C28H29ClNaO11S	631.101131	-0.8	17.0	14.0	even

Institut de Chimie Organique et Analytique
UMR 7311 - Université d'Orléans - CNRS
BP 6759 - rue de Chartres - F-45067 ORLEANS Cedex 2
cyril.colas@univ-orleans.fr
+33 (0)2 38 49 46 61
printed: 12/12/2019 19:43:48
